# A Nucleosome-Guided Map of Transcription Factor Binding Sites in Yeast

**DOI:** 10.1371/journal.pcbi.0030215

**Published:** 2007-11-09

**Authors:** Leelavati Narlikar, Raluca Gordân, Alexander J Hartemink

**Affiliations:** Department of Computer Science, Duke University, Durham, North Carolina, United States of America; The University of Tokyo, Japan

## Abstract

Finding functional DNA binding sites of transcription factors (TFs) throughout the genome is a crucial step in understanding transcriptional regulation. Unfortunately, these binding sites are typically short and degenerate, posing a significant statistical challenge: many more matches to known TF motifs occur in the genome than are actually functional. However, information about chromatin structure may help to identify the functional sites. In particular, it has been shown that active regulatory regions are usually depleted of nucleosomes, thereby enabling TFs to bind DNA in those regions. Here, we describe a novel motif discovery algorithm that employs an informative prior over DNA sequence positions based on a discriminative view of nucleosome occupancy. When a Gibbs sampling algorithm is applied to yeast sequence-sets identified by ChIP-chip, the correct motif is found in 52% more cases with our informative prior than with the commonly used uniform prior. This is the first demonstration that nucleosome occupancy information can be used to improve motif discovery. The improvement is dramatic, even though we are using only a statistical model to predict nucleosome occupancy; we expect our results to improve further as high-resolution genome-wide experimental nucleosome occupancy data becomes increasingly available.

## Author Summary

Identifying transcription factor (TF) binding sites across the genome is an important problem in molecular biology. Large-scale discovery of TF binding sites is usually carried out by searching for short DNA patterns that appear often within promoter regions of genes that are known to be co-bound by a TF. In such problems, promoters have traditionally been treated as strings of nucleotide bases in which TF binding sites are assumed to be equally likely to occur at any position. In vivo, however, TFs localize to DNA binding sites as part of a complicated thermodynamic process of cooperativity and competition, both with one another and, importantly, with DNA packaging proteins called nucleosomes. In particular, TFs are more likely to bind DNA at sites that are not occupied by nucleosomes. In this paper, we show that it is possible to incorporate knowledge of the nucleosome landscape across the genome to aid binding site discovery; indeed, our algorithm incorporating nucleosome occupancy information is significantly more accurate than conventional methods. We use our algorithm to generate a condition-dependent, nucleosome-guided map of binding sites for 55 TFs in yeast.

## Introduction

Finding functional DNA binding sites of transcription factors (TFs) throughout the genome is a necessary step in understanding transcriptional regulation. However, despite an explosion of TF binding data from high-throughput technologies like ChIP-chip ([1,2], and many more), DIP-chip [3], PBM [4], and gene expression arrays ([5,6], and many more), finding functional occurrences of binding sites of TFs remains a difficult problem because the binding sites of most TFs are short, degenerate sequences that occur frequently in the genome by chance. In particular, matches to known TF motifs in the genome often do not appear to be bound by the respective TFs in vivo. One popular explanation for this is that when the DNA is in the form of chromatin, not all parts of the DNA are equally accessible to TFs. In this state, DNA is wrapped around histone octamers, forming nucleosomes. The positioning of these nucleosomes along the DNA is believed to provide a mechanism for differential access to TFs at potential binding sites. Indeed, it has been shown that functional binding sites of TFs at regulatory regions are typically depleted of nucleosomes in vivo [7–12].

If we knew the precise positions of nucleosomes throughout the genome under various conditions, we could increase the specificity of motif finders by restricting the search for functional binding sites to nucleosome-free areas. Here, we describe a method for incorporating nucleosome positioning information into motif discovery algorithms by constructing informative priors biased toward less-occupied promoter positions. Our method should improve motif discovery most when it has access to high-resolution nucleosome occupancy data gathered under various in vivo conditions. Unfortunately, this data is not currently available for any organism at a whole-genome scale, let alone under a variety of conditions. Nevertheless, because our method is probabilistic, even noisy evidence regarding nucleosome positioning can be effectively exploited. For example, Segal et al. [12] recently published a computational model—based on high-quality experimental nucleosome binding data—that predicts the probability of each nucleotide position in the yeast genome being bound by a nucleosome; these predictions are intrinsic to the DNA sequence and thus independent of condition, but were purported to explain around half of nucleosome positions observed in vivo. In addition, Lee et al. [9] have used ChIP-chip to profile the average nucleosome occupancy of each yeast intergenic region. We show that informative positional priors, whether learned from computational occupancy predictions or low-resolution average occupancy data, significantly outperform not only the commonly used uniform positional prior, but also state-of-the-art motif discovery programs.

## Results

### Nucleosome Occupancy-Based Positional Priors

We formulate a probabilistic motif discovery framework for identifying TF motifs in sets of DNA sequences, such as those arising from ChIP-chip experiments. The goal is to find a TF motif of length *W* in a set ***X*** of sequences that are presumed to be bound by the TF. We proceed in three steps. First, we compute a score for each *W*-mer present at each position of each sequence in ***X*** that reflects the probability the TF binds the *W*-mer at that position. Second, from these scores we compute an informative “positional prior,” a non-uniform probability distribution over the positions of each sequence in ***X***. Third, we incorporated this positional prior into a search algorithm that simultaneously learns the position of a binding site in each sequence in ***X***, along with the parameters of the motif recognized by the TF. Although our method can be used with any motif model, we use a position-specific scoring matrix, or PSSM [13].

**Figure 1:**
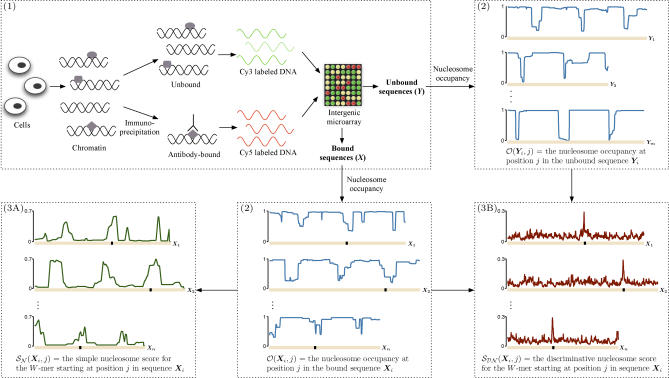
Steps in the Derivation of the Nucleosome Scores S℘_N℘_ and S℘_D℘N℘_ (1) Obtain the sets of bound and unbound sequences ({***X***_1_,***X***_2_,…,***X***_n_) and {***Y***_1_,***Y***_2_,…,***Y***_*m*_}, respectively) from a ChIP-chip experiment for a particular TF ((inidcated by a diamond). (2) Determine the nucleosome occupancy O℘ for all the bound and the unbound sequences. (3A) Compute the simple nucleosome score S℘_N℘_(***X*** *_i_*, *j*) for each *W*-mer starting at position *j* in the bound sequence ***X*** *_i_* by averaging the accessibility (1 − O℘) over all positions in the *W*-mer. (3B) Compute the discriminative nucleosome score S℘*_D℘_*_N℘_(***X*** *_i_*, *j*) for each *W*-mer starting at position *j* in sequence ***X*** *_i_*, using the accessibility (1 − O℘) over all occurrences of this *W*-mer in both the bound and the unbound sequences (see Materials and Methods for details). All the sequences and scores depicted in this figure correspond to the TF Reb1 profiled in YPD and use occupancy predictions from the computational model of Segal et al. The black boxes on the bound DNA sequences indicate matches to the Reb1 motif.

Regarding the first step, we examine two different choices of score: S℘_N℘_ and S℘_D℘N℘_ (Figure 1). The score S℘_N℘_ for a particular position is computed from the nucleosome occupancy of the *W*-mer beginning at that position. In contrast, the score S℘_D℘N℘_ for a particular position is computed from a discriminative perspective, incorporating information about the nucleosome occupancy of all occurrences of the *W*-mer in all intergenic regions, including those in the set of unbound sequences ***Y***. This builds on the observation made by Segal et al. [12] that nucleosome occupancy is lower at sites that are bound in vivo than sites that are not bound in vivo. In the second step of our method, from these two choices of score S℘_N℘_ and S℘_D℘N℘_, we build two positional priors N℘ and D℘N℘, respectively (for further details, see Materials and Methods). In the third and final step, we incorporate these two priors into a Gibbs sampling–based search method called PRIORITY [14], and we call the two variations PRIORITY-N℘ and PRIORITY-D℘N℘, respectively. To quantify the extent to which the two new informative priors N℘ and D℘N℘ improve motif discovery, we compare their performance with the performance of a uniform prior U℘. We similarly incorporate this prior into PRIORITY, and call this variation PRIORITY-U℘.

We apply all three algorithms to sequence-sets arising from ChIP-chip experiments published by Harbison et al. [2]. In assessing accuracy, we consider only the 80 TFs for which a consensus binding motif is known. These 80 TFs were profiled under various environmental conditions, resulting in a total of 156 sequence-sets where we can reasonably assess the accuracy of a motif discovery algorithm. We consider an algorithm to be successful when applied to a sequence-set if the top-scoring motif matches the literature consensus for the corresponding TF, where a match is defined as a distance of less than 0.25 (using a slight variant of the inter-motif distance measure described by Harbison et al.; see Protocol S1).

**Figure 2:**
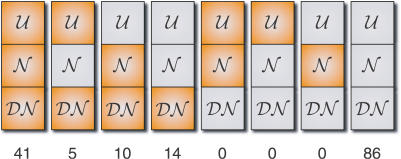
Performance of the Three Positional Priors A dark orange (light grey) square in each column indicates the situation where the respective prior succeeds (fails) in finding the true motif. There are 2^3^ = 8 possible combinations of successes or failures for the three priors. These are represented by the eight columns, which are ordered based on the success or failure of PRIORITY-D℘N℘. The number of sequence-sets (out of the total 156 sequence-sets) falling into each category is indicated below the respective column.

Figure 2 summarizes the results of the three algorithms PRIORITY-U℘, PRIORITY-N℘, and PRIORITY-D℘N℘ using this criterion on the 156 sequence-sets. Overall, PRIORITY-D℘N℘ finds the correct motif in 70 sequence-sets, resulting in an improvement of 52% over the baseline PRIORITY-U℘ which finds 46. The last four columns in Figure 2 reveal that there is no case where PRIORITY-D℘N℘ fails but PRIORITY-U℘ or PRIORITY-N℘ succeeds. In other words, the D℘N℘ prior was never harmful to motif discovery. The N℘ prior finds the true motif 51 times, not nearly as often as D℘N℘, and only marginally more often than U℘. We now discuss these results in greater detail.

### Nucleosome Occupancy Predictions Used Directly Only Marginally Improve Motif Discovery

**Figure 3:**
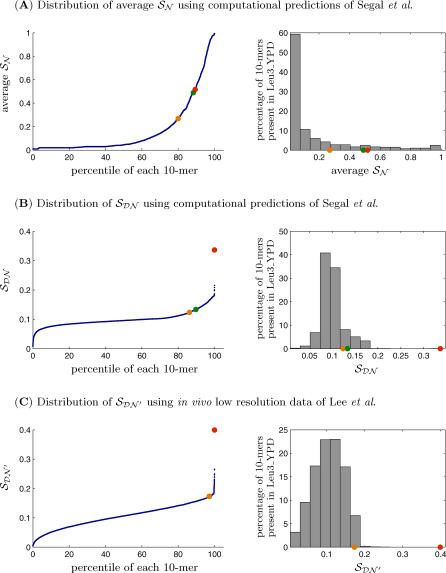
Distribution of S℘_N℘_, S℘*_D℘_*_N℘_, and S℘*_D℘_*_N℘ *′*_ in the Sequence-Set for Leu3 Profiled in YPD The distribution of scores over each unique 10-mer occurring in the Leu3_YPD sequence-set shown as a percentile plot (on the left) and as a histogram (on the right) computed according to: (A) S℘_N℘_ (averaged over each 10-mer) using predictions from computational model of Segal et al., (B) S℘*_D℘_*_N℘_ using predictions from computational model of Segal et al., and (C) S℘*_D℘_*_N℘ *′*_ using low-resolution nucleosome data from Lee et al. The three colored dots marked on each figure indicate the positions of the only three 10-mers matching the Leu3 motif CCGGNNCCGG present in Leu3_YPD. The red dot corresponds to CCGGTACCGG (see text). The mass to the right of the dots in each graph reveals the fraction of 10-mers scoring higher.

We expect the simple nucleosome prior N℘ to perform well when functional binding sites of the profiled TF are generally less occupied by nucleosomes than other locations within the same DNA sequence. One instance where this is known to occur is in sequences bound by Leu3, since the experimental data of Liu et al. [15] show that loci bound by Leu3 in vivo are typically depleted of nucleosomes. As expected, PRIORITY-N℘ finds the true motif of Leu3 in both of the environments where it was profiled by Harbison et al. When Leu3 is profiled in SM, PRIORITY-U℘ also succeeds, but when profiled in YPD, PRIORITY-U℘ fails. We take a closer look at this case to understand better why prior N℘ is more effective in identifying the true motif of Leu3. To do so, we calculate the average S℘_N℘_ score for each 10-mer present in the Leu3_YPD sequence-set (Figure 3A). Leu3 is known to recognize the 10-mer CCGGNNCCGG, with a slight preference for CCGGTACCGG [15,16], and indeed we find that fewer than 10% of 10-mers score higher than CCGGTACCGG, revealing that the prior N℘ is assigning a higher prior probability to positions containing the true motif.

Although PRIORITY-N℘ is more successful than PRIORITY-U℘ overall (51 successes versus 46), the second column in Figure 2 reveals that in five sequence-sets, PRIORITY-U℘ performs better than PRIORITY-N℘. The score S℘_N℘_ used to compute the prior N℘ reflects the accessibility of the *W*-mer at a particular position. While it is true that regions bound by the profiled TF should be accessible, it does not follow that every accessible region is bound by the profiled TF. Some accessible regions could be binding sites of other TFs or other functional DNA elements. Indeed, in four of the five cases where PRIORITY-U℘ does better, PRIORITY-N℘ finds a motif rich in A's and T's; it has been previously shown that many yeast promoters contain poly(dA-dT) sequences that stimulate transcription [17]. Furthermore, due to their intrinsic DNA structure, poly(dA-dT) sequences are often free of nucleosomes, and they are believed to increase TF accessibility by delocalizing nucleosomes in vivo [17–19]. Since PRIORITY-N℘ is expected to find highly accessible DNA sequences that occur often in a given set of bound promoters, it is not surprising that it sometimes finds poly(dA-dT) sequences. However, we notice that such sequences occur often and are accessible not only in the bound set ***X***, but also in the rest of the genome, so they are not specific to the profiled TF.

### Nucleosome Occupancy Predictions Used in a Discriminative Manner Significantly Improve Motif Discovery

**Figure 4:**
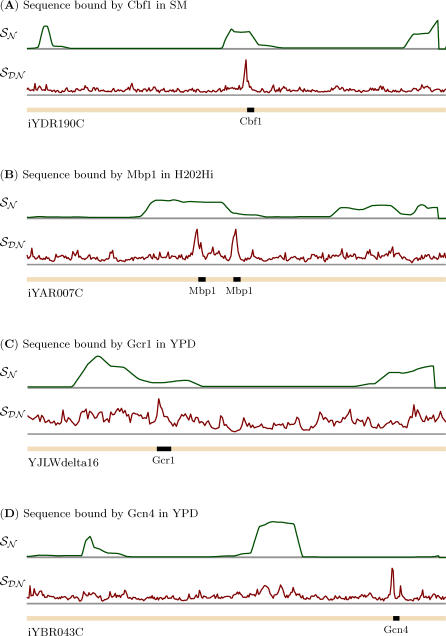
Nucleosome Occupancy and the Values of S℘*_D℘_*_N℘_ over Four Intergenic Sequences (A) iYDR190C in Cbf1_SM, (B) iYAR007C in Mbp1_H2O2Hi, (C) YJLWdelta16 in Gcr1_YPD, and (D) iYBR043C in Gcn4_YPD. The boxes indicate binding sites annotated by Harbison et al. [2]. S℘*_D℘_*_N℘_ at the locations of each of these binding sites has a high value relative to the rest of the sequence regardless of the S℘_N℘_ score at those sites. In particular, in spite of the low accessibility at the binding sites of Gcr1 (in YJLWdelta16) and Gcn4 (in iYBR043C), S℘*_D℘_*_N℘_ correctly indicates a high prior probability at those regions.

The computation of the score S℘_D℘N℘_ used to compute the prior D℘N℘ addresses the issue of nucleosome-free regions that are not specific to the profiled TF. A ChIP-chip experiment gives rise to sequences that are bound by the profiled TF as well as those that are not bound. Using both these sets of sequences, each *W-*mer in the bound set can be scored according to how many times it occurs in each set, as well as how accessible it is in each set. This discriminates between sites that are highly accessible only in the bound set and sites that are highly accessible throughout the genome. The former are more likely to be true binding sites of the profiled TF. Figure 4 shows a range of examples where S℘_D℘N℘_ is able to correctly upweight the prior probability of the location of the true binding site.

When we perform the same word-analysis for S℘_D℘N℘_ in Leu3_YPD as we did for S℘_N℘_, we see that S℘_D℘N℘_ is even better at predicting the true binding site than S℘_N℘_ (Figure 3B). In fact, no 10-mer has an S℘_D℘N℘_ score higher than CCGGTACCGG, the known consensus Leu3 binding site.

In 14 sequence-sets, motif discovery benefits from nucleosome occupancy information only when this information is used in a discriminative manner (column 4 in Figure 2). We perform an analysis for S℘_D℘N℘_ in these sequence-sets similar to the one we did earlier for S℘_N℘_ in Leu3_YPD. For simplicity, we restrict our attention to the nine sequence-sets which have a known literature consensus of length less than ten bases (see Figure S1). In seven of the nine cases, fewer than 5% of the S℘_D℘N℘_ scores are better than that of the true motif; the average over all nine being only 8%. The corresponding average for S℘_N℘_ is 39%; in three of the nine cases, more than 50% of the scores are better than that of the true motif (even with a uniform prior, the number should be only 50% in expectation, implying that in these cases, S℘_N℘_ is worse than uniform). Thus, it is not surprising that when PRIORITY-U℘ fails in these cases, PRIORITY-N℘ also fails.

**Figure 5:**
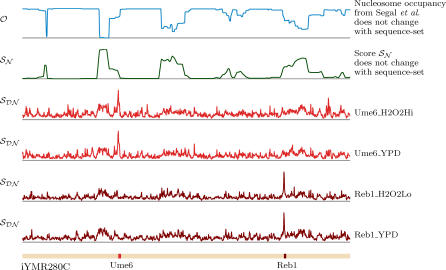
S℘*_D℘_*_N℘_ over a Single Sequence Belonging to Multiple Sequence-Sets The intergenic region iYMR280C belongs to four sequence-sets: Ume6_H2O2Hi, Ume6_YPD, Reb1_H2O2Lo, and Reb1_YPD. The boxes indicate binding sites annotated by Harbison et al. [2]. S℘*_D℘_*_N℘_ for each sequence-set is different although S℘_N℘_ does not change. S℘*_D℘_*_N℘_ indicates correctly the location of the binding site of the respective TF.

Note that the prior N℘ over a particular intergenic sequence does not change regardless of which TF binds it. However, since S℘_D℘N℘_ is computed using both bound and unbound sequences, the prior D℘N℘ can be different over the same sequence depending on the TF that binds it. Figure 5 shows the different S℘_D℘N℘_ scores computed over the intergenic sequence iYMR280C which belongs to four sequence-sets: Reb1_H2O2Lo, Reb1_YPD, Ume6_H2O2Hi, and Ume6_YPD. Figure 5 demonstrates the specificity toward binding sites of only the profiled TF when the nucleosome prior is computed from a discriminative perspective.

### PRIORITY-D℘N℘ Outperforms State-of-the-Art Motif Finders, Including Those Using Conservation

We compiled results from six state-of-the-art motif discovery programs as reported by Harbison et al. on the same 156 sequence-sets: AlignACE [20] finds 16, MEME [21] finds 35, MDscan [22] finds 54, MEME_c [2] finds 49, a method by Kellis et al. [23] finds 50, and CONVERGE [2] finds 56 correct motifs. Each of these methods makes use of different sources of information for motif discovery. AlignACE and MEME use different search techniques (Gibbs sampling and Expectation Maximization [24]), but use no additional information and thus are directly comparable to PRIORITY-U℘. MDscan uses *p*-values resulting from the ChIP-chip experiments, while the last three programs make use of sequence conservation across various species of yeast. PRIORITY-D℘N℘, with 70 correct motifs, outperforms all these methods. Table S1 shows the performance of each program in detail.

### PRIORITY-D℘N℘ Identifies True TF–DNA Interactions for TFs Involved in Multiple Transcriptional Complexes

**Figure 6:**
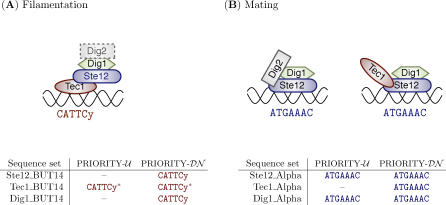
Transcriptional Complexes Involving Ste12, Tec1, and Dig1 (A) During filamentation, Ste12 forms a complex with Dig1 and Tec1. Tec1 binds DNA, with a sequence specificity for CATTCy. PRIORITY-D℘N℘ finds this motif in all three sequence-sets pulled down by Ste12, Tec1, and Dig1 after the cells are treated with butanol. However, PRIORITY-U℘ misses the functional Tec1 motif in Ste12_BUT14 and Dig1_BUT14. The asterisk indicates that the learned motif is a weak match. (B) During mating, Ste12 forms two complexes: one with Dig1 and Dig2, and another with Dig1 and Tec1. In either case, it is Ste12 that binds DNA, with a sequence specificity for ATGAAAC. Again, PRIORITY-D℘N℘ finds this motif in all three sequence-sets pulled down by Ste12, Tec1, and Dig1 after the cells are treated with the alpha factor pheromone. Here, PRIORITY-U℘ fails to find the Ste12 motif in Tec1_Alpha. (Figures of the complexes are adapted from Chou et al. [25].)

PRIORITY-D℘N℘ is able to capture true protein–DNA interactions even in the case of TFs that form multiple complexes, such as Ste12. It has been shown experimentally that Ste12 is part of two distinct complexes, Ste12/Dig1/Dig2 and Tec1/Ste12/Dig1, which control two distinct transcriptional programs: filamentation and mating [25]. Chou et al. [25] show that the promoters of most filamentation genes are bound by the Tec1/Ste12/Dig1 complex, with Tec1 binding DNA directly (Figure 6A). The promoters of most mating genes, however, are bound by either the Ste12/Dig1/Dig2 or the Tec1/Ste12/Dig1 complex, with Ste12 binding DNA directly in both cases (Figure 6B). Dig1 is not currently known to have a DNA binding site, and a literature search did not reveal any evidence of Dig1 binding DNA directly.

In the experiments of Harbison et al. [2], Dig1, Ste12, and Tec1 were all profiled after treatment with alpha factor for 30 min (Alpha) and after treatment with butanol for 14 h (BUT14). In all six sequence-sets corresponding to the three TFs in Alpha and BUT14, both the Tec1 binding site (CATTCy) and the Ste12 binding site (ATGAAAC) occur often and are statistically significantly enriched. However, taking into account the experimental results of Chou et al., and the fact that butanol treatment induces the expression of filamentation genes, one would expect that in BUT14, the Tec1 binding site is the real site of interaction between DNA and the transcriptional complex Tec1/Ste12/Dig1 (Figure 6A). Indeed, when we run our algorithm PRIORITY-D℘N℘ on the sequence-sets Ste12_BUT14, Tec1_BUT14, and Dig1_BUT14, the learned motif in all three cases is the Tec1 motif (CATTCy), as shown in Figure 6A.

On the other hand, treatment with the alpha factor pheromone induces the expression of mating genes, and therefore in Alpha one would expect both Dig1 and Tec1 to bind DNA indirectly through Ste12 (Figure 6B). Indeed, the Ste12 motif (ATGAAAC) was reported by PRIORITY-D℘N℘ for all three sequence-sets, Ste12_Alpha, Tec1_Alpha, and Dig1_Alpha. In both Ste12_BUT14 and Tec1_Alpha sequence-sets, PRIORITY-U℘ fails to find a motif matching either the Ste12 or the Tec1 motif. Interestingly, the average predicted nucleosome occupancy of Ste12 and Tec1 binding sites in Ste12_BUT14 is 0.91 and 0.84, respectively, and in Tec1_Alpha is 0.81 and 0.90, respectively. In other words, Tec1 binding sites are less occupied by nucleosomes in Ste12_BUT14, while Ste12 binding sites are less occupied in Tec1_Alpha. This fact is exploited successfully by PRIORITY-D℘N℘.

### Novel Motif Predictions Using PRIORITY-D℘N℘

For every input sequence-set, PRIORITY-D℘N℘ returns the top-scoring motif along with its score (see Protocol S1 for the computation of the score). To assess whether a motif score is significant, we run PRIORITY-D℘N℘ on 50 randomly generated sequence-sets of the same cardinality. The observed scores from these random sequence-sets of a particular cardinality are well-fit by a normal distribution. Thus, each motif learned by PRIORITY-D℘N℘ on a particular ChIP-chip sequence-set can be assigned an empirical *p*-value calculated from this distribution. Figure S2 shows the motifs learned from the 156 sequence-sets of TFs with literature consensus DNA binding sites, along with their *p*-values.

We can plot precision-recall and receiver operating characteristic curves based on the *p*-values of these known motifs (Figure S3). For a given *p*-value cutoff, we notice that in many false positive instances, PRIORITY-D℘N℘ finds a high-scoring motif that resembles TGTGTGTG or CACACACA. Poly(GT/CA) tracts are known to be common in yeast [26], so for the remainder of this part of the analysis we disregard sequence-sets for which PRIORITY-D℘N℘ learns a motif of this form. For the others, we can use the precision-recall curve to estimate the false discovery rate (FDR) of our novel predictions.

A consensus DNA binding motif was not known for 67 of the TFs profiled by Harbison et al. at the time the ChIP-chip experiments were performed. These 67 TFs were profiled under various environmental conditions, yielding a total of 82 sequence-sets. We run PRIORITY-D℘N℘ on these sequence-sets and obtain the top-scoring motif, along with its score. As before, we compute the *p*-values of each of the learned motifs (Figure S4). At a *p*-value of 5.0 × 10^−6^, we estimate the FDR to be less than 15%. Of the 82 new motifs, 14 have a *p*-value lower than 5.0 × 10^−6^ when we exclude motifs resembling TGTGTGTG; our FDR estimate would suggest that 12 of these are likely to be correct. Two motifs are for Dig1_Alpha and Dig1_BUT90. As expected, the motif learned from Dig1_Alpha resembles the Ste12 motif, while the motif learned from Dig1_BUT90 resembles the Tec1 motif (see Figure 6). Another significant motif is that of Rfx1_YPD and the binding site of Rfx1 now listed in TRANSFAC 11.1 matches the learned motif.

We construct a condition-dependent, nucleosome-guided map of TF binding sites derived from these 14 motifs, along with the 72 matching the literature consensus (including the Tec1 motif learned in Ste12_BUT90 and the Ste12 motif learned in Tec1_Alpha). The 86 sequence-sets correspond to 55 TFs profiled in one or more of ten environmental conditions. In their ChIP-chip experiments, Harbison et al. report a total of 2,387 promoter sequences to be bound by one of these TFs. Our map contains a total of 2,347 high-confidence TF binding sites within these sequences.

### Use of Low Resolution In Vivo Nucleosome Occupancy Data also Significantly Improves Motif Discovery

Lee et al. [9] report results from ChIP-chip experiments where the densities of histones H3 and H4 are profiled over the whole genome. This in vivo nucleosome occupancy data is at a resolution of approximately one kilobase, so we cannot use it to obtain distinct scores over individual nucleotide positions. However, we can still use it to weight entire intergenic regions in a discriminative manner. We first use a logit transformation to map the reported intensity over each intergenic region into a probability (see Materials and Methods). We then assume that each position within a sequence has an occupancy probability equal to the occupancy probability of the whole sequence, and compute a new version of the S℘_D℘N℘_ score, which we call S℘_D℘N℘*′*_.

Figure 3C shows the distribution of the S℘_D℘N℘*′*_ scores of all 10-mers present in Leu3_YPD. As in the case of the S℘_D℘N℘_ score, S℘_D℘N℘*′*_ assigns the 10-mer CCGGTACCGG the highest rank, which is encouraging. Indeed, the corresponding prior, which we call D℘N℘*′*, performs admirably overall as well: PRIORITY-D℘N℘*′* learns a total of 66 motifs correctly. A more detailed look shows that it does worse than PRIORITY-D℘N℘ in seven sequence-sets, but better in three. Since this nucleosome occupancy data is obtained in YPD, one might expect the benefits to be primarily in sequence-sets obtained from TFs profiled in YPD. However, of the three sequence-sets where D℘N℘*′* does better, two are not in YPD. Perhaps the nucleosome landscape does not change much across various environmental conditions for these TFs; this has been shown to be true in the case of certain TFs, like the heat shock protein Hsf1 [11]. Or perhaps these represent sequence-sets where the computational model on which D℘N℘ is based is not as accurate as the low-resolution in vivo data.

### The Prior D℘N℘ Reduces to a Simple, but Effective Discriminative Prior When No Nucleosome Occupancy Data Is Available

What happens when nucleosome occupancy data is not available? In this case, a special version of the D℘N℘ prior can be computed in which the occupancy is assumed to be uniform over all sequences (note that this is different from D℘N℘*′* where the occupancy is assumed to be uniform over the positions within each individual sequence, but may change across sequences). The information in this simple discriminative prior derives not from any nucleosome data whatsoever, but only from the sequence content of the bound and the unbound sets. The Gibbs sampler incorporating this prior correctly identifies 60 true motifs, demonstrating the utility of a discriminative perspective. Although not as effective as PRIORITY-D℘N℘ or PRIORITY-D℘N℘′, the improvement of 30% of this prior over U℘ is nevertheless significant. Detailed results obtained using this prior are available in Table S1.

## Discussion

Although it has been known for a while that nucleosomes modulate the binding activity of TFs by providing differential access to DNA binding sites [7–12], we believe we are the first to use nucleosome occupancy information to more accurately predict de novo binding sites of TFs. To be clear, we do not assume that nucleosomes bind DNA first and that TFs bind whatever remains accessible (nor the other way around). Rather, we imagine that nucleosomes and TFs are together in competition for positions on the genome and their binding configurations are sampled from a thermodynamic statistical ensemble. All other things being equal, places where nucleosomes bind strongly may be places where TFs are less likely to successfully compete, and, conversely, places where TFs bind strongly may be places where nucleosomes are less likely to successfully compete. In this manner, a high probability of nucleosome occupancy suggests that a TF binding site is less likely. We show that while nucleosome occupancy used as a simple positional prior only marginally improves the performance of a motif discovery algorithm, when it is used to compute a discriminative prior—taking into account accessibility over the whole genome—the accuracy of motif discovery improves dramatically.

In situations where no nucleosome occupancy information is available, the prior D℘N℘ simplifies to a new kind of informative prior that can exploit discriminative information from the bound and unbound sequences in a purely generative setting. The prior performs admirably, finding 30% more true motifs than the uniform prior. The use of unbound sequences has previously been shown to improve both enumerative and probabilistic motif discovery approaches. Enumerative discriminative approaches compute the significance of the enrichment of every *W*-mer in the bound versus the unbound set using hypergeometric [27] or binomial distributions [28,29]. These methods are fast, but they usually work better when the TF binding sites have limited sequence variability [30]. Probabilistic approaches [31–35] attempt to learn the parameters of a discriminative motif that appears often in the bound set but less often in the unbound set. Since these discriminative sequence models try to distinguish between bound and unbound sets, they must traverse an enormous search space and become hampered by many local optima. In addition, at every step of the search algorithm, they have to evaluate the parameters of the model on each sequence in both sets. In contrast, while our prior D℘N℘ is calculated in a discriminative manner, the motif discovery problem itself remains formulated in a generative setting. Consequently, PRIORITY-D℘N℘ only needs to sample over the bound set, causing the overall time and space complexities of the search to be much less than those of other discriminative approaches (even for the largest sequence-set Cbf1_SM with 194 sequences, PRIORITY-D℘N℘ takes fewer than four minutes on a desktop machine with a 2.4 GHz Intel Core2 CPU). Our discriminative approach can be viewed as a combination of both enumerative and probabilistic learning: the prior is primarily computed using “word counts” over bound and unbound sets, while the actual motif discovery is carried out using Gibbs sampling to optimize a posterior distribution. Our final motif retains the discriminative information through the prior contribution to the posterior objective function. Also, our discriminative approach is general enough to handle not only nucleosome occupancy information, but other kinds of biological data such as conservation, local DNA structure, etc.

Throughout the paper, we have used PSSMs to model motifs. Although the PSSM is a popular choice for a motif model, recent biological [36] and computational [37,38] findings indicate that more expressive (and hence, more complex) models might be more appropriate. Since our method assigns a prior on the locations within each sequence and not on any specific form of the motif model, it is not tied to the PSSM model, but can be used with any motif model. In addition, although we have focused on ChIP-chip data here, both our priors N℘ and D℘N℘ can be computed for data resulting from other large-scale experimental methodologies such as gene expression, PBM, and DIP-chip microarrays.

In closing, we stress that incorporating informative priors over sequence positions is of great benefit to motif discovery algorithms. Low signal-to-noise ratio, especially in higher organisms, makes it difficult to successfully use algorithms based only on statistical overrepresentation. Narlikar et al. [14] have shown that using informative priors based on structural classes of TFs improves motif discovery, and this paper shows that other kinds of informative priors improve motif discovery as well. Although PRIORITY-U℘ performs better than AlignACE and MEME, it falls short of the other four programs described earlier which use additional information like *p*-values or sequence conservation, illustrating the general utility of additional information in motif discovery. Additionally, although PRIORITY-D℘N℘ does better overall than these conservation-based methods, certain motifs are found by one or more of these methods but not by PRIORITY-D℘N℘ (Table S1). This suggests that combining conservation and nucleosome occupancy might further improve the performance of motif finders.

## Materials and Methods

### TF ChIP-chip data.

We compiled ChIP-chip data published by Harbison et al. [2], who profiled the intergenic binding locations of 203 yeast TFs under various environmental conditions: always YPD (rich medium) and sometimes one or more of Acid (acidic medium), Alpha (alpha factor pheromone treatment), BUT14 (butanol treatment for 14 h), BUT90 (butanol treatment for 90 min), GAL (galactose medium), H202Hi (highly hyperoxic), H202Lo (mildly hyperoxic), HEAT (elevated temperature), Pi− (phosphate deprived medium), RAFF (raffinose medium), RAPA (nutrient deprived), SM (amino acid starvation), or THI− (vitamin deprived) over 6,140 intergenic regions. For each TF, we define its sequence-set ***X*** for a particular condition to be those intergenic sequences reported to be bound with *p*-value < 0.001 in that condition. We denote the set of all other sequences, those that are bound by that TF with a higher *p*-value, as the unbound set ***Y***. Each sequence-set ***X*** is represented as TF_ condition. We restrict our attention to sequence-sets of size at least 10, which yields 238 sequence-sets, encompassing 147 TFs. Of these sequence-sets, 156 correspond to the 80 TFs with a consensus binding motif in the literature (as summarized by Harbison et al. at the time their paper was published, or as earlier reported by Dorrington and Cooper [39] or Jia et al. [40]), and these 156 are used throughout the paper to compare the performance of various motif-finding algorithms. The remaining 82 sequence-sets, corresponding to 67 TFs with unknown binding motifs, are used to make novel motif predictions.

### PRIORITY: Sequence model and optimization.

Assume the profiled TF is reported to bind a sequence-set ***X*** containing *n* DNA sequences ***X***_1_ to ***X*** *_n_*. Although in reality each bound sequence might have multiple binding sites, we model only one binding site in each sequence for simplicity. Because the experimental data might be erroneous, we also model the possibility that some sequences have no binding site. This is analogous to the zero or one occurrence per sequence (ZOOPS) model in MEME [21]. Let ***Z*** be a vector of length *n* denoting the starting location of the binding site in each sequence: *Z_i_* = *j* if there is a binding site starting at location *j* in ***X*** *_i_* and we adopt the convention that *Z_i_* = 0 if there is no binding site in ***X*** *_*i*_*. We assume that the TF motif can be modeled as a PSSM of length *W* parameterized by ***φ*** while the rest of the sequence follows some background model parameterized by ***φ***_0_. We present results here for *W* set to 8.

We wish to find *φ* and ***Z*** that maximize the joint posterior distribution of all the unknowns given the data. Assuming two independent priors *P*(***φ***) and *P*(***Z***) over ***φ*** and ***Z***, respectively, our objective function is:

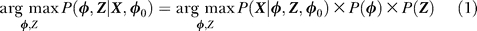



We use Gibbs sampling to sample repeatedly from the posterior over ***φ*** and ***Z*** so that we are likely to visit those values of ***φ*** and ***Z*** with the highest posterior probability (see Protocol S1). We run the Gibbs sampler, which we call PRIORITY [14], for a predetermined number of iterations after apparent convergence to the joint posterior and output the highest-scoring PSSM at the end. Although PRIORITY generates a posterior sample which is useful for other analyses in the style of MCMC, here we use only the single best motif ***ϕ*** to evaluate the algorithm and compare it with other popular methods. The source code of PRIORITY and the data used in the paper can be downloaded from http://www.cs.duke.edu/~amink.

### Computation of positional priors.

The prior on the positions *P*(***Z***) in Equation 1 is assumed to be uniform in conventional motif discovery algorithms. We call such a prior U℘. Here, we discuss two informative positional priors based on nucleosome occupancy information. We assume we have this information as O℘(*S*, *j*): the probability of the *j*th position in sequence *S* being occupied by a nucleosome.

*Simple nucleosome prior* **N℘**. We use O℘(*S*, *j*) to compute a simple nucleosome score S℘_N℘_(***X*** *_i_*, *j*) for each *W*-mer starting at position *j* in the bound sequence ***X*** *_i_*:

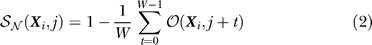



We use this score to compute a positional prior N℘ which can be used in motif discovery. Note that the values S℘_N℘_(***X*** *_i_*, *j*) themselves do not define a probability distribution over *j*. S℘_N℘_(***X*** *_i_*, *j*) is only the probability that the *W*-mer at location *j* in ***X*** *_i_* is a binding site of the profiled TF. As mentioned earlier, we model each sequence ***X*** *_i_* as containing at most one such binding site. If ***X*** *_i_* has no such binding site, none of the positions of ***X*** *_i_* can be the starting location of such a binding site, so it must be that:

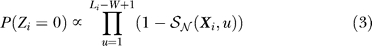
where *L_i_* is the length of sequence ***X*** *_i_*. On the other hand, if ***X*** *_i_* has one such binding site at position *j*, not only must a binding site start at location *j* but also no such binding site should start at any of the other locations in ***X*** *_i_*. Formally, we write:

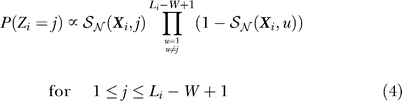



We then normalize *P*(*Z_i_*) using the same proportionality constant in Equations 3 and 4, so that under the assumptions of our model we have:

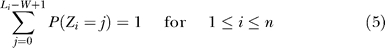



*Discriminative nucleosome prior* **D℘N℘**. In addition to DNA sequences ***X***, which are bound by the profiled TF, genome-wide ChIP-chip experiments also produce DNA sequences not bound by the TF. Assume we get *m* such sequences ***Y*** *_1_* to ***Y*** *_m_*. We compute a discriminative nucleosome score S℘*_D℘_*_N℘_(***X*** *_i_*, *j*) by taking into account the occupancies O℘ over both sets ***X*** and ***Y***. For each *W*-mer in ***X***, we ask the following question: “Of all the accessible occurrences of this *W*-mer, what fraction occur in the bound set?” The motivation behind this is to ensure a high score for *W*-mers that are accessible only in the bound set but not for *W*-mers that are accessible in general throughout the genome. To answer this question, we subject each accessible *W*-mer to a Bernoulli trial. Since we only know the probability that a certain location is accessible, we count the number of accessible *W*-mers in expectation, weighing each occurrence of the *W*-mer according to how accessible it is. Using the S℘_N℘_ scores derived from O℘ over both sets ***X*** and ***Y***, we calculate S℘*_D℘_*_N℘_(***X*** *_i_*, *j*) as:

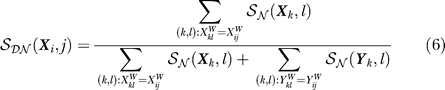
where 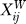 is the *W*-mer starting at location *j* in sequence ***X*** *_i_*.

As in the case of S℘_N℘_(***X*** *_i_*, *j*), S℘*_D℘_*_N℘_(***X*** *_i_*, *j*) is only the probability that the *W*-mer 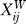 is a binding site of the profiled TF. To convert these values into a positional prior, we substitute S℘*_D℘_*_N℘_ for S℘_N℘_ in Equations 3 and 4. After normalizing the resulting *P*(*Z_i_*) as in Equation 5, we get the positional prior D℘N℘.

### Nucleosome occupancy data.

*Predictions from computational model.* We applied the computational model learned by Segal et al. [12] over the whole yeast genome (March 2006 version). We used the resulting nucleosome occupancy predictions directly as O℘(*S*, *j*) for each position *j* in an intergenic sequence *S*.

*Low-resolution in vivo data*. We used the whole-genome ChIP-chip results for Myc-tagged H4 and H3 published by Lee et al. [9]. We used the median H4 intensity ratios (the authors obtained nearly identical results for H3 and H4) which range from −1.757 (least occupied) to 1.112 (most occupied) and converted them to probabilities using a logit transformation to get occupancy O℘:

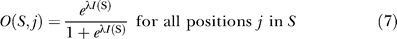
where *I*(*S*) is the log ratio of intensities (H4-Myc ChIP versus input genomic DNA), and *λ* is the logit parameter. We tried three different values of *λ* (1, 4, and 10) and noted results did not change significantly. Here, we report the best results, obtained with *λ* = 10. We call the variant of S℘_D℘N℘_ computed with the low-resolution data S℘_D℘N℘′_, and the prior derived from it D℘N℘*′*. Note that the S℘_N℘_ derived from this data is the same over all positions within a sequence, and thus not very informative. We therefore present results of only the D℘N℘*′* prior here.

## Supporting Information

Figure S1 Distribution of S℘_N℘_ and S℘_D℘N℘_ Scores in Nine Sequence-Sets (A I) Represents the nine sequence-sets out of the 156 considered, where PRIORITY-D℘N℘ succeeds while both PRIORITY-U℘ and PRIORITY-N℘ fail. The scores in this figure are calculated over *W*-mers where *W* is set to the true motif length. Known binding sites are indicated with red dots on the curve. In almost each sequence-set, the true binding sites fall in a higher percentile when scored using S℘_D℘N℘_ than S℘_N℘_. If we call *W*-mers that score higher than the true binding sites “distractors” for motif discovery, we notice that in most cases, the S℘_D℘N℘_ score of the binding site is higher than the S℘_N℘_ score, relative to the respective S℘_D℘N℘_ and S℘_N℘_ scores of the distractors. Thus, in terms of both the number of words scoring higher than the binding site (toward the right of the *x*-axis) and the relative value of the binding site score with respect to scores of distractors (toward the top of the *y*-axis), S℘_D℘N℘_ is better. [More text included with the figure.]
(3.1 MB PDF)

Figure S2 Motifs Learned by PRIORITY-D℘N℘ on 156 Sequence-Sets with Known Motifs The motifs are ranked according to their *p*-values. The *p*-values are computed from the normal distribution of scores learned on random sequence-sets with the same cardinality.
(1.6 MB PDF)

Figure S3 Use of *p*-Values to Detect Significant Motifs We compute *p*-values for each motif learned from the 156 sequence-sets with known motifs (see Figure S2). After removing nine motifs resembling the poly(GT) tracts, we are left with 70 that match the literature (which we call true positives) and 77 that do not match the literature (which we call false positives). To find out how well the *p*-value differentiates between the true and the false positives, we plot the (A) precision-recall curve and (B) receiver operating characteristic curve. We can thus find a *p*-value cutoff that yields a low FDR and use it to predict novel motifs with high confidence. As an example, both figures show an operating point of *p*-value 5.0 × 10^−6^, where the FDR is less than 15%. This is the operating point mentioned in the text.
(59 KB PDF)

Figure S4 Novel Motifs Learned by PRIORITY-D℘N℘ on 82 Sequence-Sets The motifs are ranked according to their *p*-values. The *p*-values are computed from the normal distribution of scores learned on random sequence-sets with the same cardinality.
(89 KB PDF)

Protocol S1 Supplementary Methods (91 KB PDF)

Table S1 Comparison of PRIORITY Using Various Positional Priors with State-of-the-Art Motif Discovery Programs (93 KB PDF)

## Abbreviations

FDR: false discovery rate
PSSM: position-specific scoring matrix
TF: transcription factor
