# Supplementary material for: A Nucleosome-Guided Map of Transcription Factor Binding Sites in Yeast
Source: PLoS Comput Biol. 2007 Nov 9;3(11):e215. doi: 10.1371/journal.pcbi.0030215 (PMC2065891; doi:10.1371/journal.pcbi.0030215)
Supplement: Supplementary file 1 [file pcbi.0030215.sd001.pdf]

Supplementary methods

Sequence model and objective function

Assume the profiled TF binds a sequence-set $\boldsymbol{X}$ containing n DNA sequences $\boldsymbol{X}_1$ to $\boldsymbol{X}_n$. We model the possibility of each sequence $\boldsymbol{X}_i$ having exactly one or no binding site. Let $\boldsymbol{Z}$ be a vector of length n denoting the starting location of the binding site in each sequence: $Z_i = j$ if there is a binding site starting at location j in $\boldsymbol{X}_i$ and we adopt the convention that $Z_i = 0$ if there is no binding site in $\boldsymbol{X}_i$. We assume that the TF motif can be modeled as a PSSM of length W parameterized by $\boldsymbol{\phi}$ while the rest of the sequence follows some background model parameterized by $\boldsymbol{\phi}_0$. The PSSM can be described by a matrix $\boldsymbol{\phi}$ where $\phi_{a,b}$ is the probability of finding base b at location a within the binding site for $1 \leq b \leq 4$ and $1 \leq a \leq W$.

Thus if the sequence $\boldsymbol{X}_i$ is of length l_i, and $\boldsymbol{X}_i$ contains a binding site at location Z_i, we can compute the probability of the sequence given the model parameters as:

$$P(\boldsymbol{X}_i \mid \boldsymbol{\phi}, Z_i > 0, \boldsymbol{\phi}_0) = P(X_{i,1}, \ldots X_{i,Z_i-1} \mid \boldsymbol{\phi}_0) \times \left(\prod_{a=1}^{W} \phi_{a, X_{i,Z_i+a-1}} \right) \times P(X_{i,Z_i+W}, \ldots X_{i,m_i} \mid \boldsymbol{\phi}_0)$$

and if it instead does not contain a binding site as:

$$P(\boldsymbol{X}_i \mid \boldsymbol{\phi}, Z_i = 0, \boldsymbol{\phi}_0) = P(X_{i,1}, X_{i,2} \ldots X_{i,m_i} \mid \boldsymbol{\phi}_0)$$

We wish to find $\boldsymbol{\phi}$ and $\boldsymbol{Z}$ that maximize the joint posterior distribution of all the unknowns given the data. Assuming priors $P(\boldsymbol{\phi})$ and $P(\boldsymbol{Z})$ over $\boldsymbol{\phi}$ and $\boldsymbol{Z}$ respectively, our objective function is:

$$\arg\max_{\boldsymbol{\phi},\boldsymbol{Z}} P(\boldsymbol{\phi}, \boldsymbol{Z} \mid \boldsymbol{X}, \boldsymbol{\phi}_0) = \arg\max_{\boldsymbol{\phi},\boldsymbol{Z}} P(\boldsymbol{X} \mid \boldsymbol{\phi}, \boldsymbol{Z}, \boldsymbol{\phi}_0) P(\boldsymbol{\phi}) P(\boldsymbol{Z}) \quad (1)$$

Optimization strategy and scoring scheme

As others before us have done, we use Gibbs sampling to sample repeatedly from the posterior over $\boldsymbol{\phi}$ and $\boldsymbol{Z}$ with the hope that we are likely to visit those values of $\boldsymbol{\phi}$ and $\boldsymbol{Z}$ with the highest posterior probability. Gibbs sampling is a Markov chain Monte Carlo (MCMC) method that approximates sampling from a joint posterior distribution by sampling iteratively from individual

conditional distributions [1]. Applying the collapsed Gibbs sampling strategy developed by Liu [2] for faster convergence, we can integrate out $\boldsymbol{\phi}$ and sample only the Z_i. This results in the following expression for sampling Z_i from its conditional distribution assuming the prior on $\boldsymbol{Z}$ to be independent of the PSSM parameters $\boldsymbol{\phi}$:

$$P(Z_i \mid \boldsymbol{Z}_{[-i]}, \boldsymbol{X}, \boldsymbol{\phi}_0) = \frac{P(\boldsymbol{Z} \mid \boldsymbol{X}, \boldsymbol{\phi}_0)}{P(\boldsymbol{Z}_{[-i]} \mid \boldsymbol{X}, \boldsymbol{\phi}_0)} = \frac{P(\boldsymbol{Z}) \int_{\boldsymbol{\phi}} P(\boldsymbol{X} \mid \boldsymbol{\phi}, \boldsymbol{Z}, \boldsymbol{\phi}_0) P(\boldsymbol{\phi}) \mathrm{d}\boldsymbol{\phi}}{P(\boldsymbol{Z}_{[-i]}) \int_{\boldsymbol{\phi}} P(\boldsymbol{X} \mid \boldsymbol{\phi}, \boldsymbol{Z}_{[-i]}, \boldsymbol{\phi}_0) P(\boldsymbol{\phi}) \mathrm{d}\boldsymbol{\phi}}$$

where $\boldsymbol{Z}_{[-i]}$ is the vector $\boldsymbol{Z}$ without Z_i. Proceeding analogously to the derivation of Liu [2], we compute the integrals using a Dirichlet prior on $\boldsymbol{\phi}$. We further simplify the sampling expression by dividing it by $P(Z_i = 0, \boldsymbol{X}_i \mid \boldsymbol{\phi}_0)$ which is a constant at a particular sampling step. This results in the following sampling distribution for a particular location j within sequence $\boldsymbol{X}_i$, similar to the predictive update formula as described in Liu *et al.* [3]:

$$P(Z_i = j \mid \boldsymbol{Z}_{[-i]}, \boldsymbol{X}, \boldsymbol{\phi}_0) = \frac{P(Z_i = j) \times \left(\prod_{a=1}^{W} \phi_{a, X_{i,j+a-1}} \right)}{P(Z_i = 0) \times P(X_{i,j}, \ldots, X_{i,j+W-1} \mid \boldsymbol{\phi}_0)} \tag{2}$$

for $1 \leq j \leq l_i - W + 1$, and

$$P(Z_i = j \mid \boldsymbol{X}, \boldsymbol{\phi}_0) = 1 \tag{3}$$

for $j = 0$, where $\boldsymbol{\phi}$ is calculated from the counts of the sites contributing to the current alignment $\boldsymbol{Z}_{[-i]}$, plus the pseudocounts as determined by the Dirichlet prior. More details are provided in Narlikar *et al.* [4].

The joint posterior distribution after each iteration can be calculated as:

$$P(\boldsymbol{\phi}, \boldsymbol{Z} \mid \boldsymbol{X}, \boldsymbol{\phi}_0) \propto P(\boldsymbol{X} \mid \boldsymbol{\phi}, \boldsymbol{Z}, \boldsymbol{\phi}_0) \times P(\boldsymbol{\phi}) \times P(\boldsymbol{Z}) \tag{4}$$

To simplify the computation, we divide the above expression by $P(\boldsymbol{X} \mid \boldsymbol{Z} = \mathbf{0}, \boldsymbol{\phi}_0)$ which is a constant, and use the logarithm of the resulting value as a score for the motif.

To maximize the objective function and hence the score, we run the Gibbs sampler 10 times from random initializations for a predetermined number of iterations each (10000 in the results presented here) after apparent convergence to the joint posterior and output the highest scoring PSSM at the end. We report only a single motif $\boldsymbol{\phi}$ to enable us to evaluate the algorithm and compare it with other popular methods. In principle, however, since we are using an MCMC

sampling method, we could instead perform Bayesian model averaging over many samples from the posterior and report a mean motif (or multiple motifs if there are multiple modes in the distribution).

Inter-motif distance

We calculate the inter-motif distance between the learned motif and the literature consensus based on distance metric constructed by Harbison *et al.* [5]. They define the distance D between two aligned motifs ϕ and ϕ' as:

$$D(\phi, \phi') = \frac{1}{\omega} \sum_{i=1}^{\omega} \frac{1}{\sqrt{2}} \sum_{L \in \{ACGT\}} (\phi_{i,L} - \phi'_{i,L})^2$$

where ω is the aligned motif width, and ϕ and ϕ' are parameters of two PSSMs.

To determine the optimal alignment between two motifs, we use the minimum distance between motifs among all possible alignments (including reverse complements) in which the motifs overlap by at least six bases. If the smaller motif is shorter than six bases, we ensure that all bases of the smaller motif are used in the optimal alignment. We include an additional constraint that the average entropy of the learned motif must be at least 1 in the overlapping region. We noticed that without this entropy constraint, low entropy motifs or motifs with a mismatch at important nucleotide bases were incorrectly labeled as true motifs.

In general, we use a distance cutoff of 0.25 to declare whether a motif learned from a particular sequence-set matches the literature consensus or not. However, in cases where a TF has multiple sequence-sets arising from ChIP-chip experiments performed under different environmental conditions, we also allow a motif to be declared a match if it satisfies all three of the following conditions: (1) its distance to the literature consensus is less than 0.30, (2) its distance to all of the other learned motifs for the same TF in the other environmental conditions is less than 0.15, and (3) the distance of the literature consensus to all of the other learned motifs for the same TF in the other environmental conditions is less than 0.25 (i.e., all the other motifs are matches under the original distance criterion). This second criterion was added after we noticed that in rare instances, motifs of the same TF arising from different environmental conditions would be nearly identical to one another, but one would just miss the 0.25 cutoff while the others would all make it.

Although increasing or decreasing the distance cutoff of 0.25 correspondingly changes the number of motifs called correct, the general trend of the total number of correctly learned motifs across all programs (both PRIORITY-based and other state-of-the-art programs) remains the same, so the relative results are generally insensitive to a range of reasonable choices for this cutoff. We acknowledge that our distance function is imperfect and probably not as accurate as visual inspection in determining whether two motifs match, but we chose an automated method in order to reduce the possibility of introducing subjective bias into our results.

Motifs derived from literature

We used the set of literature consensus sequences which were compiled from Transfac, YPD, or SCPD by Harbison *et al.* prior to the publication of their ChIP-chip data. We further supplemented the set with binding site information reported by Dorrington and Cooper [6] and Jia *et al.* [7] for two TFs Dal82 and Rtg1, respectively.

We converted the consensus sequences into PSSM motifs by using the base at each position in the same manner as Harbison *et al.*:

- At a consensus position, we assigned the particular base a probability of 0.964, with 0.012 being assigned to each of the other three possibilities.
- At a degenerate position with two possible base values, we assigned those bases a probability of 0.488 each, and 0.012 for the other two.
- At a position where we had an 'N', we assigned an equal probability of 0.25 for each base.

The genome

We used the March 2006 genome when computing the nucleosome occupancy predictions using the model from Segal *et al.* [8] The probes used by Harbison *et al.* are based on an older version of the genome (2004). We therefore took into account the changes from the older version to the 2006 version, and translated the probe coordinates accordingly to get the bound sequence-sets from the 2006 genome. We notice the FASTA files of the sequence-sets do not change much; indeed, most remain exactly the same.

Nucleosome-guided map of TF binding sites

Using our 86 high-confidence motifs (14 newly predicted and 72 that match literature consensus), we scan the sequences in each of the corresponding 86 sequence-sets. A site is considered to be a binding site if its probability under the respective PSSM is at least half the maximum possible probability under that PSSM. We compile these predictions into ten tracks corresponding to the ten environmental conditions from which the 86 sequence-sets were derived. In addition, we also produce an eleventh track that integrates the binding site information across all conditions. These eleven tracks are available as GFF files.

References

1. Gelfand A, Smith A (1990) Sampling based approaches to calculating marginal densities. Journal of the American Statistical Association 85: 398–409.

2. Liu J (1994) The collapsed Gibbs sampler with applications to a gene regulation problem. Journal of the American Statistical Association 89: 958–966.

3. Liu J, Neuwald A, Lawrence C (1995) Bayesian models for multiple local sequence alignment and Gibbs sampling strategies. Journal of the American Statistical Association 90: 1156–1170.

4. Narlikar L, Gordân R, Ohler U, Hartemink A (2006) Informative priors based on transcription factor structural class improve *de novo* motif discovery. Bioinformatics 22: e384–e392.

5. Harbison C, Gordon D, Lee T, Rinaldi N, Macisaac K *et al.* (2004) Transcriptional regulatory code of a eukaryotic genome. Nature 431: 99–104.

6. Dorrington R, Cooper T (1993) The DAL82 protein of *Saccharomyces cerevisiae* binds to the DAL upstream induction sequence (UIS). Nucleic Acids Research 21: 3777–3784.

7. Jia Y, Rothermel B, Thornton J, Butow R (1997) A basic helix-loop-helix-leucine zipper transcription complex in yeast functions in a signaling pathway from mitochondria to the nucleus. Molecular and Cellular Biology 17: 1110–1117.

8. Segal E, Fondufe-Mittendorf Y, Chen L, Thastrom A, Field Y *et al.* (2006) A genomic code for nucleosome positioning. Nature 442: 772–778.
