# Supplementary material for: A Nucleosome-Guided Map of Transcription Factor Binding Sites in Yeast
Source: PLoS Comput Biol. 2007 Nov 9;3(11):e215. doi: 10.1371/journal.pcbi.0030215 (PMC2065891; doi:10.1371/journal.pcbi.0030215)
Supplement: Supplementary file 2 [file pcbi.0030215.sg001.pdf]

(**A**) Cin5_H202Hi sequence-set: Cin5 is known to recognize `TTAC[A/G]TAA`

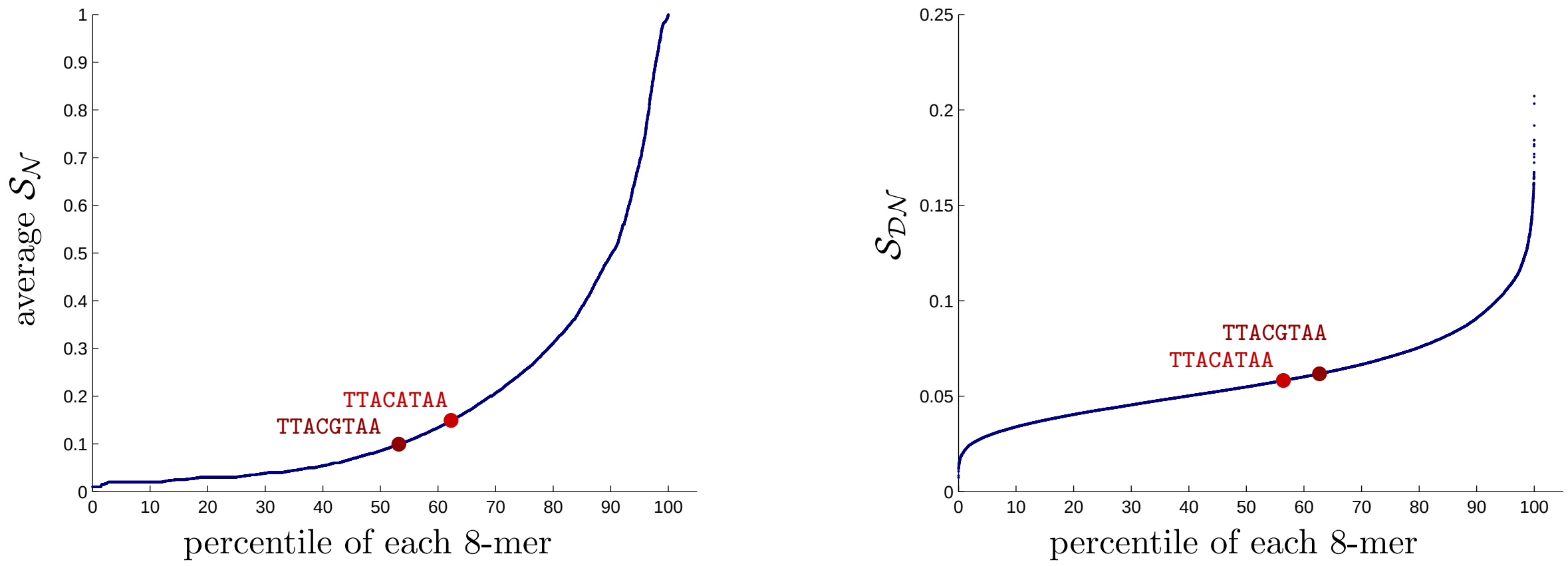

(**B**) Cin5_YPD sequence-set: Cin5 is known to recognize `TTAC[A/G]TAA`

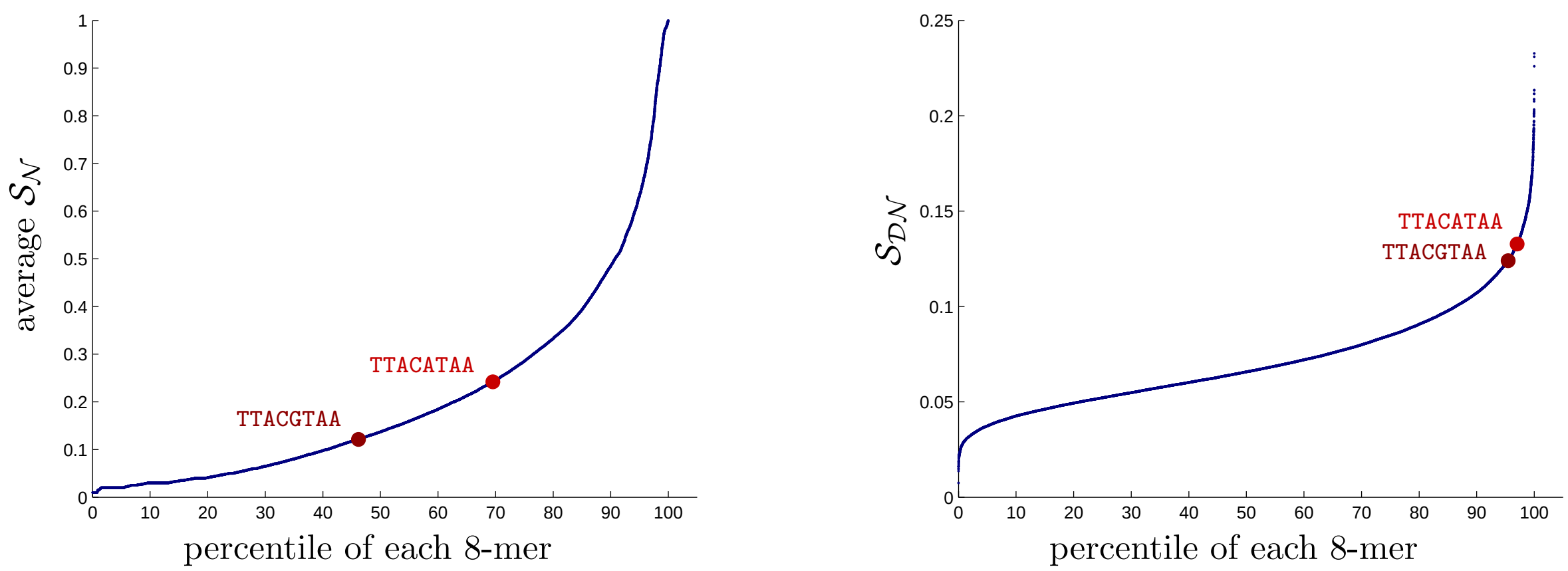

(**C**) Gat1_RAPA sequence-set: Gat1 is known to recognize `GATAA`

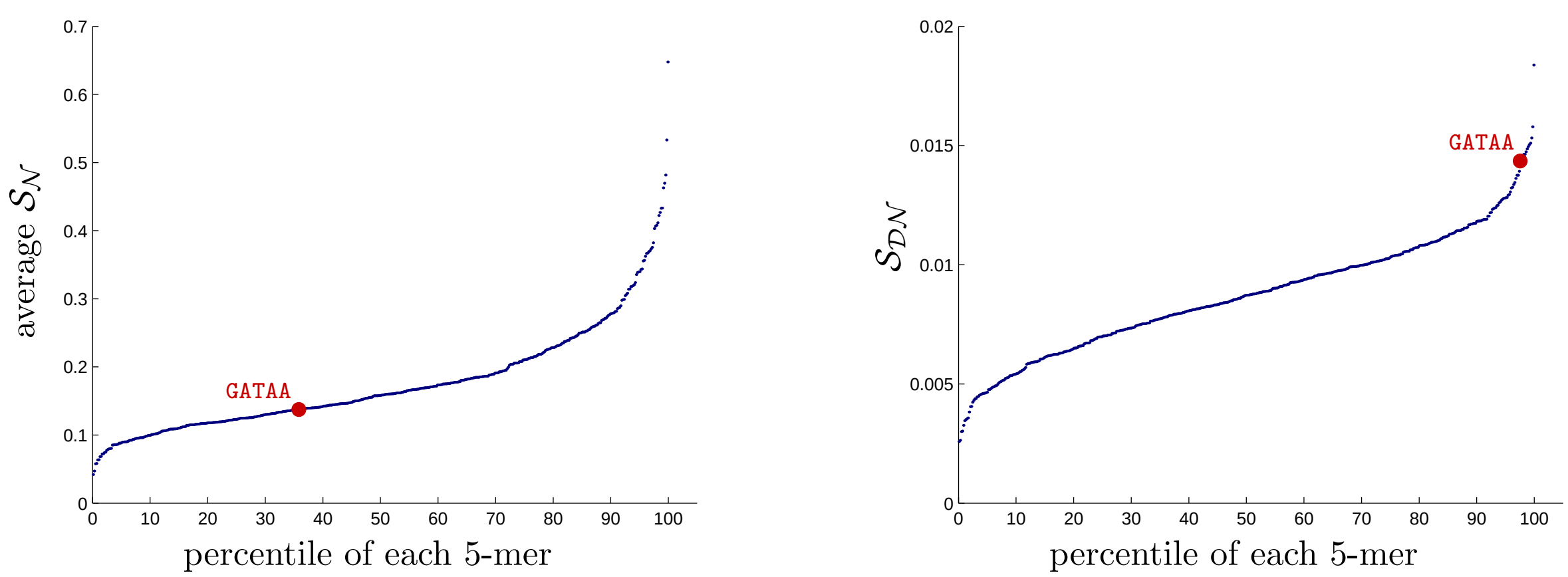

(**D**) Gcr1_YPD sequence-set: Gcr1 is known to recognize GGCTTCC[A/T]C

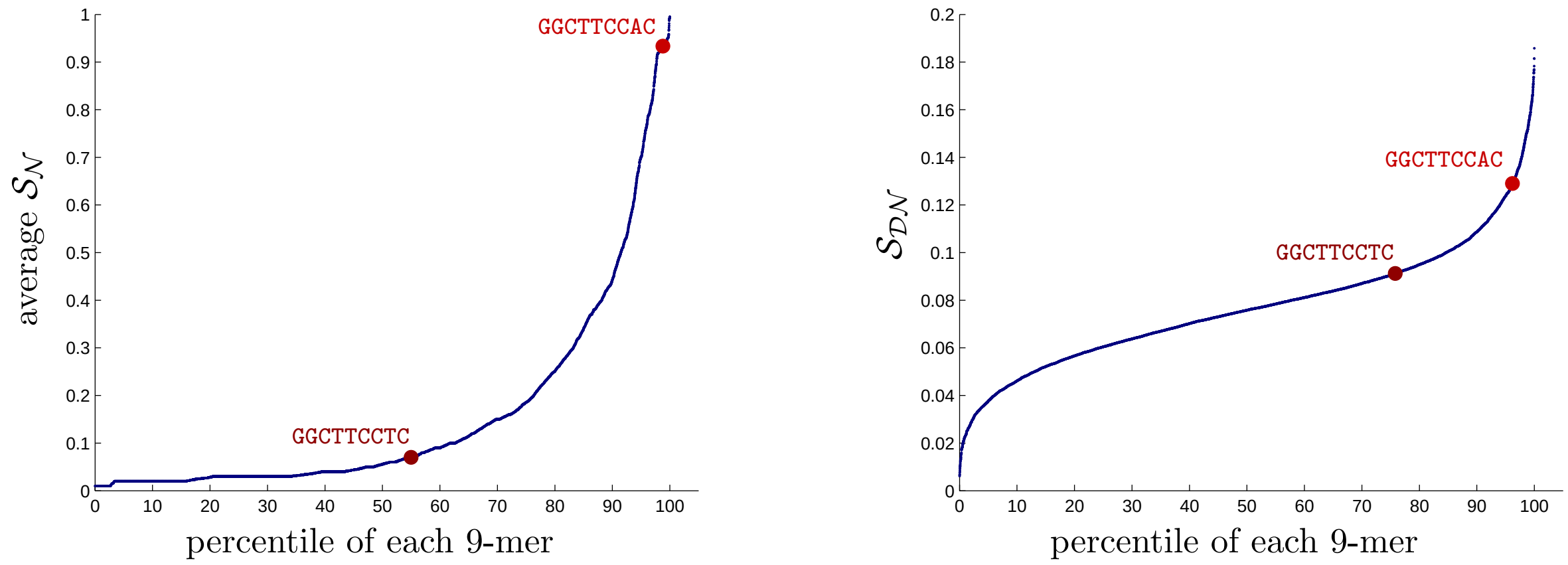

(**E**) Hap2_RAPA sequence-set: Hap2 is known to recognize CCAAT

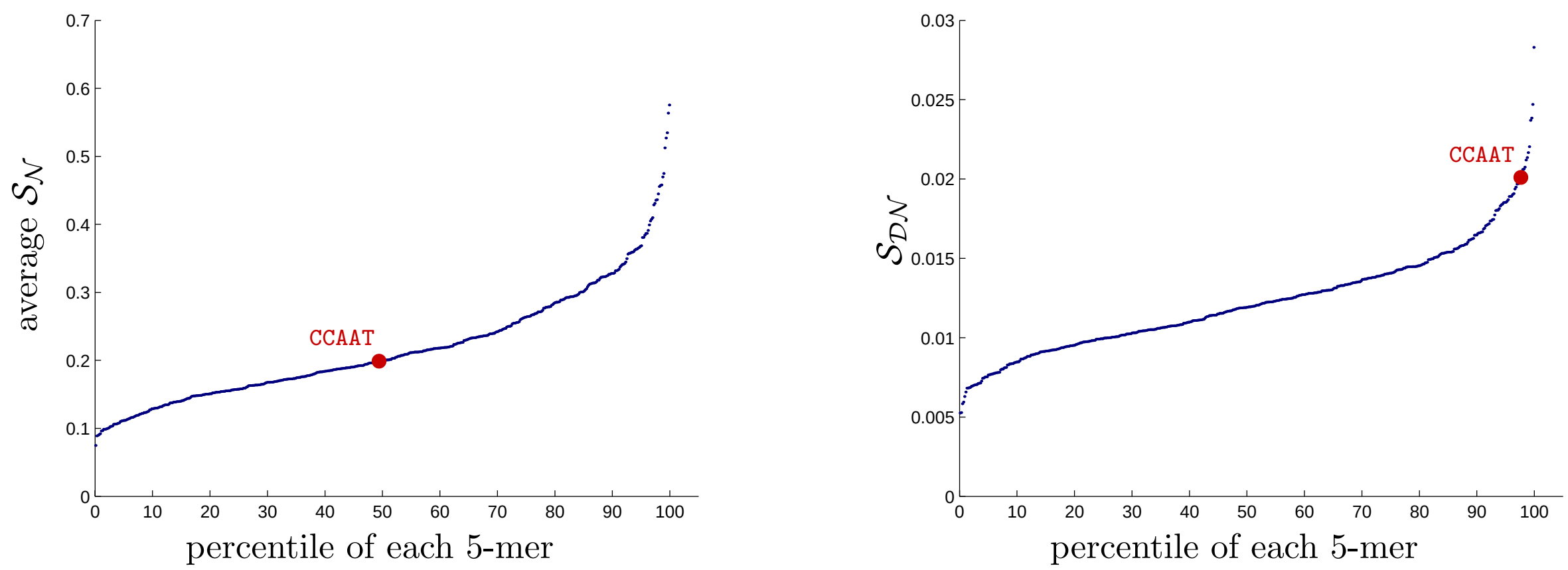

(**F**) Mac1_H202Hi sequence-set: Mac1 is known to recognize GAGCAAA

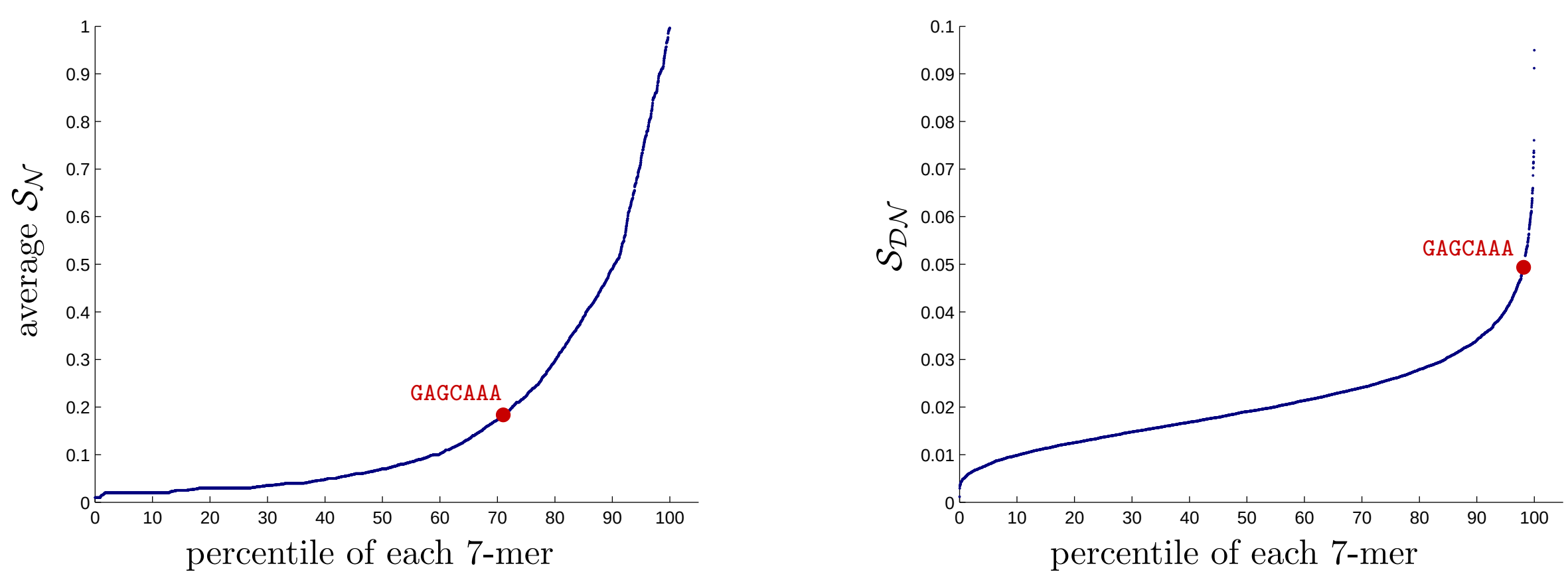

(**G**) Mac1_YPD sequence-set: Mac1 is known to recognize `GAGCAAA`

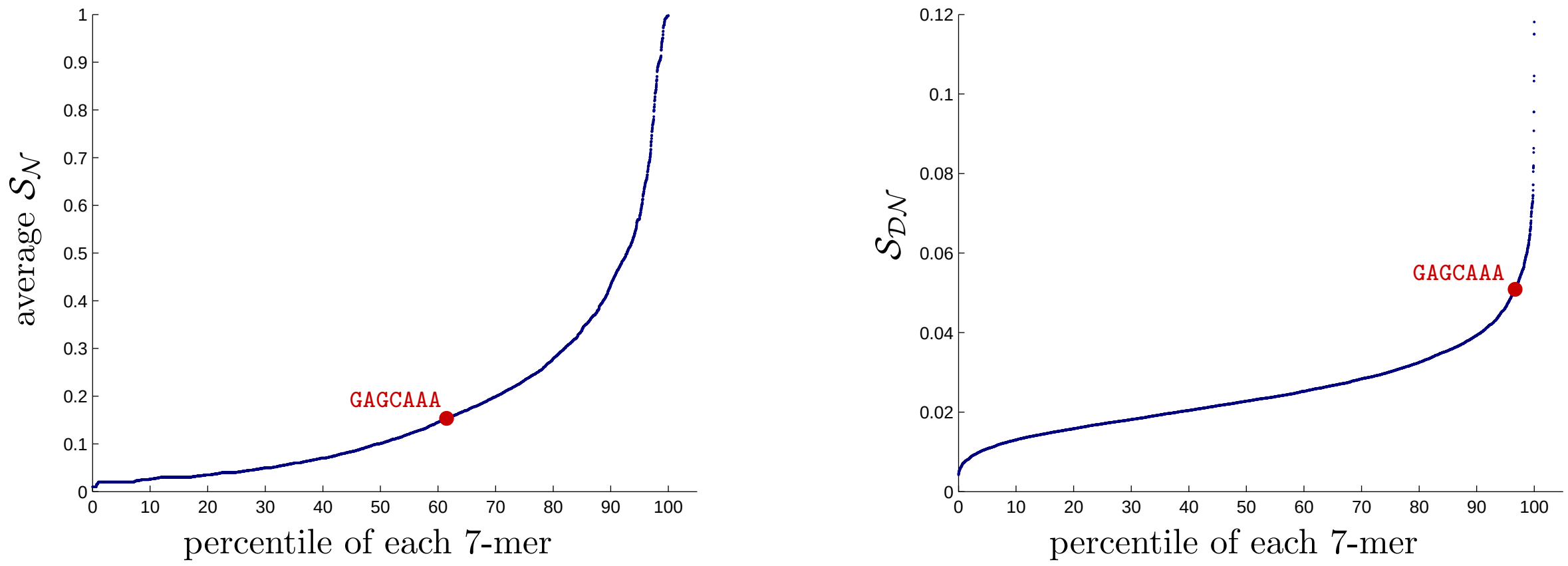

(**H**) Sko1_YPD sequence-set: Sko1 is known to recognize `ACGTCA`

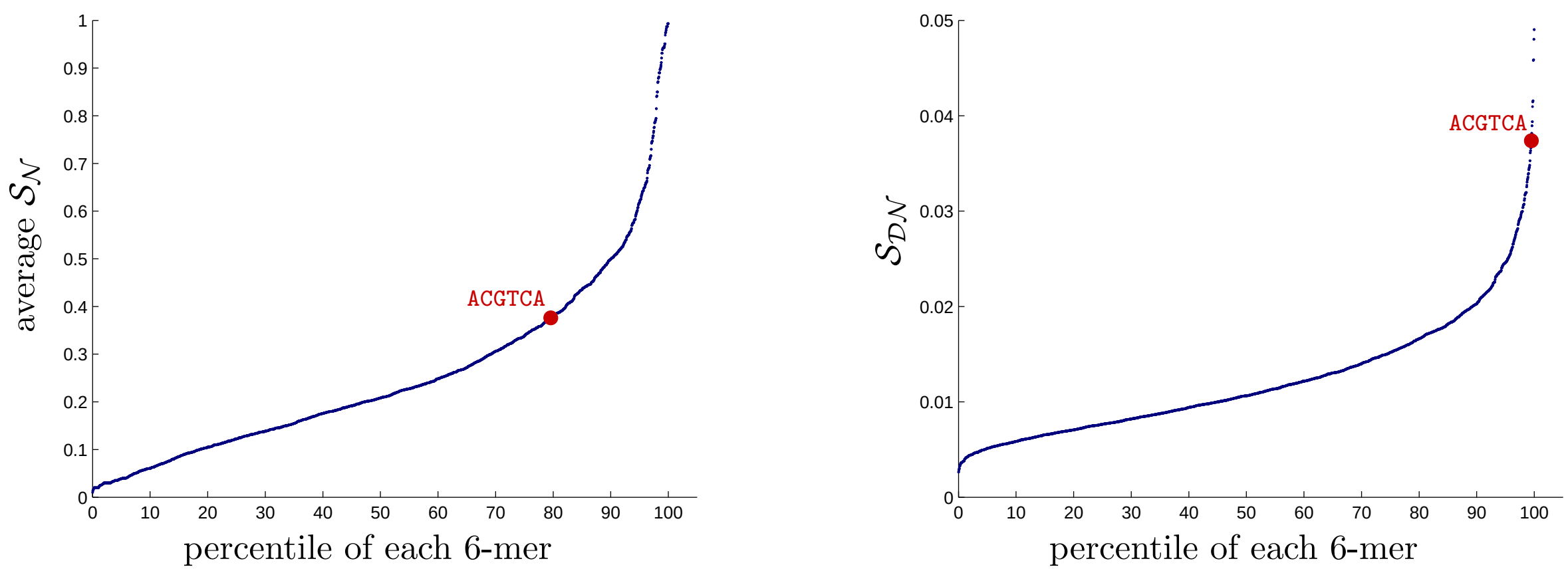

(**I**) Ste12_BUT90 sequence-set: Ste12 is known to recognize `ATGAAAC`

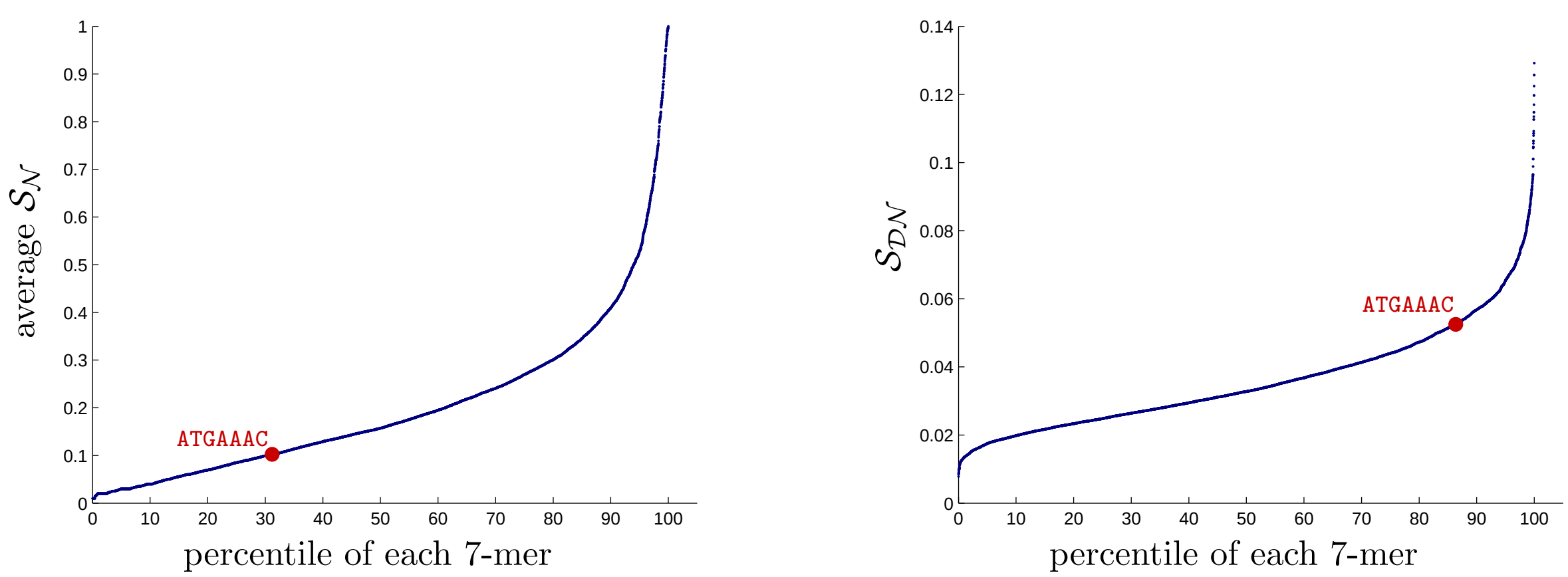

Figure S1. Distribution of average $\mathcal{S}_{\mathcal{N}}$ and $\mathcal{S}_{\mathcal{DN}}$ scores in nine sequence-sets. (**A**)–(**I**) represent the nine sequence-sets out of the 156 considered, where PRIORITY-$\mathcal{DN}$ succeeds while both PRIORITY-$\mathcal{U}$ and PRIORITY-$\mathcal{N}$ fail. The scores in this figure are calculated over W-mers where W is set to the true motif length. Known binding sites are indicated with red dots on the curve. In almost each sequence-set, the true binding sites fall in a higher percentile when scored using $\mathcal{S}_{\mathcal{DN}}$ than $\mathcal{S}_{\mathcal{N}}$. If we call W-mers that score higher than the true binding sites 'distractors' for motif discovery, we notice that in most cases, the $\mathcal{S}_{\mathcal{DN}}$ score of the binding site is higher than the $\mathcal{S}_{\mathcal{N}}$ score, relative to the respective $\mathcal{S}_{\mathcal{DN}}$ and $\mathcal{S}_{\mathcal{N}}$ scores of the distractors. Thus in terms of both the *number* of words scoring higher than the binding site (towards the right of the X-axis) and the *relative* value of the binding site score with respect to scores of distractors (towards the top of the Y-axis), $\mathcal{S}_{\mathcal{DN}}$ is better.

Cases (**D**) and (**I**) are of special interest. In the Gcr1_YPD sequence-set, the binding site `GGCTTCCAC` scores slightly higher in terms of percentile and a lot higher in terms of the relative score of distractors when the score is computed using $\mathcal{S}_{\mathcal{N}}$. Further investigation showed that there is only one copy of each "known" binding site in the whole sequence-set of 24 sequences. Naturally, Gcr1 must bind other 9-mers close to the true motif. We looked at the eight distinct 9-mers (having at least one copy in the sequence-set) with exactly one mismatch with one of the two binding sites. For all but two of these eight, the $\mathcal{S}_{\mathcal{DN}}$ score is better, relatively as well as percentile based. For a motif to be learned, all sites used in the creation of the motif must generally have a high prior probability. We believe this is the reason PRIORITY-$\mathcal{N}$ fails to find the true motif. We also scored the motif learned by PRIORITY-$\mathcal{DN}$ using the $\mathcal{N}$ prior to see if by chance PRIORITY-$\mathcal{N}$ was stuck in some local optimum, but found this was not the case.

In case of Ste12_BUT90, where Ste12 is profiled in cells treated with butanol for 90 minutes, PRIORITY-$\mathcal{DN}$ finds a motif matching `ATGAAAC`. As discussed in the paper, Ste12 forms a complex with Tec1 and Tec1 binds DNA at `CATTCy` during filamentaion (which is induced upon butanol treatment). However, during mating, Ste12 makes DNA contact at `ATGAAAC`. We notice that the Tec1 binding sites are also highly enriched in this sequence-set (not shown). In fact, in the 10 runs of the Gibbs sampler from different random initializations, although the top scoring motif was that of Ste12, the second best motif was that of Tec1. We suspect 90 minutes of butanol treatment puts the cell in an intermediate state in terms of filamentation. (The results discussed in paper are in cells treated with butanol for 14 hours, where the sequences are more strongly enriched for Tec1 binding sites.)
