# Supplementary material for: A Nucleosome-Guided Map of Transcription Factor Binding Sites in Yeast
Source: PLoS Comput Biol. 2007 Nov 9;3(11):e215. doi: 10.1371/journal.pcbi.0030215 (PMC2065891; doi:10.1371/journal.pcbi.0030215)
Supplement: Supplementary file 3 [file pcbi.0030215.sg002.pdf]

No.	Sequence-set	Motif#	*p*-value	Match to literature
1	ABF1_YPD	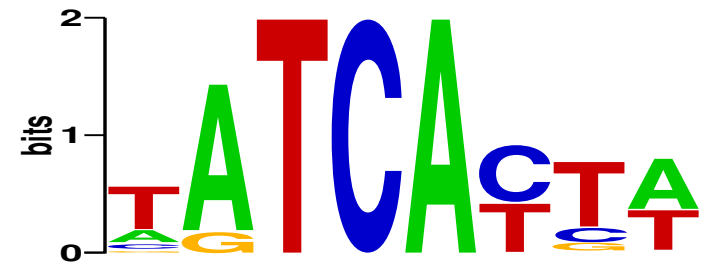	0	Yes
2	ACE2_YPD	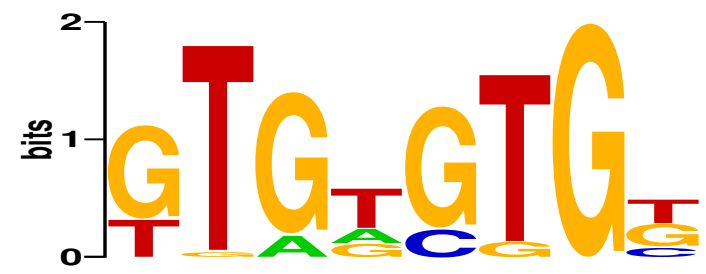	0	No*
3	AFT2_H2O2Hi	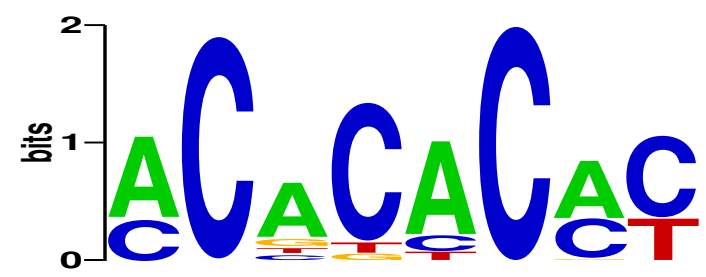	0	Yes*
4	AFT2_H2O2Lo	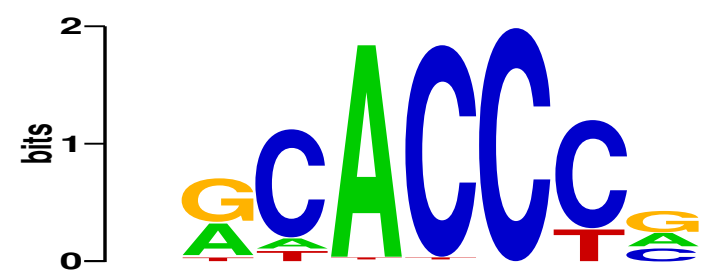	0	Yes
5	BAS1_YPD	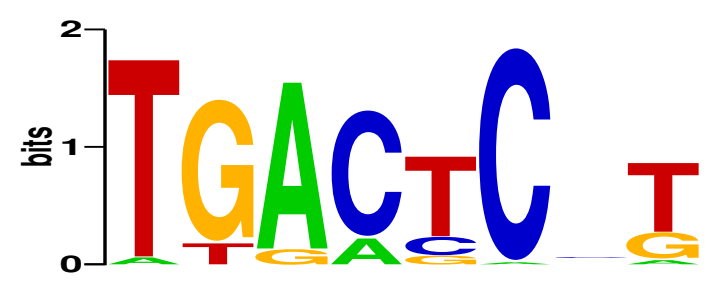	0	Yes
6	CBF1_SM	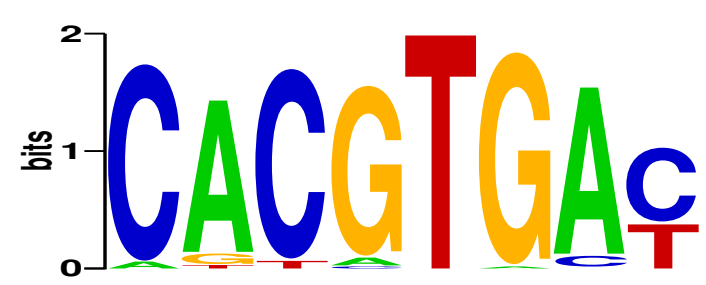	0	Yes
7	CBF1_YPD	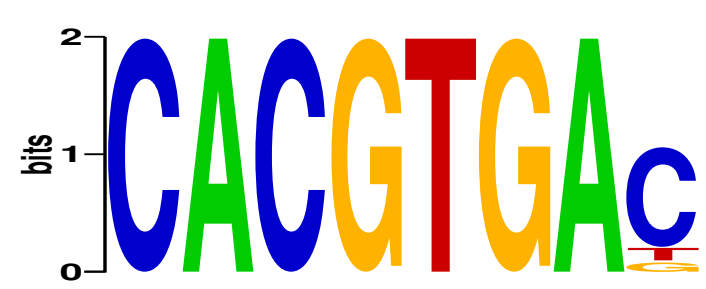	0	Yes
8	CIN5_YPD	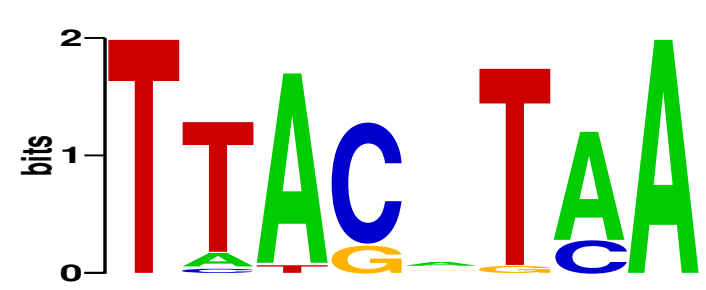	0	Yes
9	DAL81_RAPA	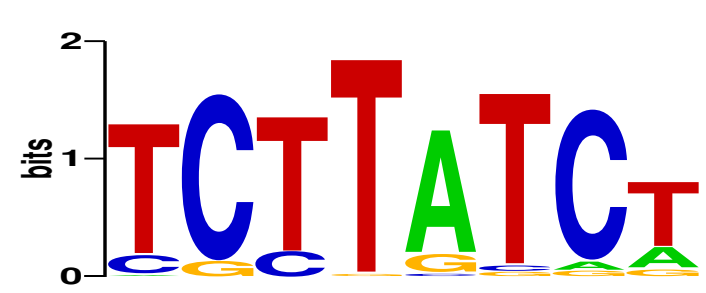	0	No
10	FKH1_YPD	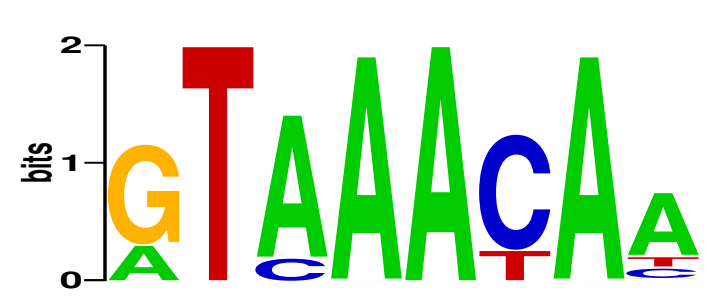	0	Yes

No.	Sequence-set	Motif	p-value	Match to literature
11	FKH2_H2O2Hi	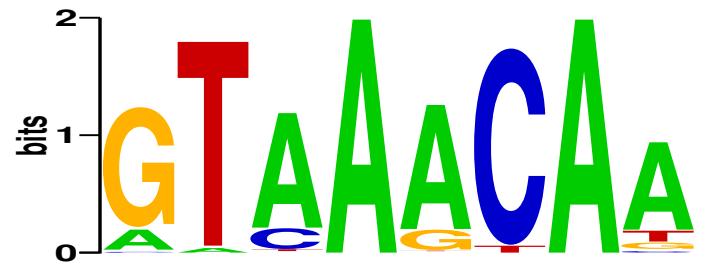	0	Yes
12	FKH2_YPD	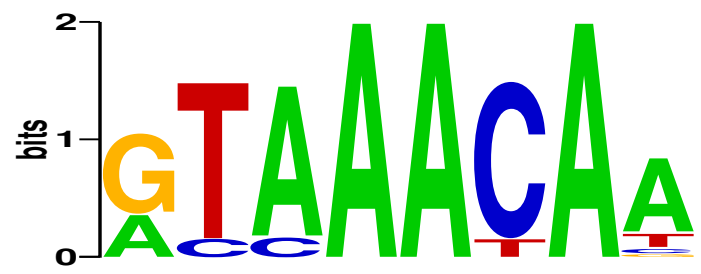	0	Yes
13	GCN4_RAPA	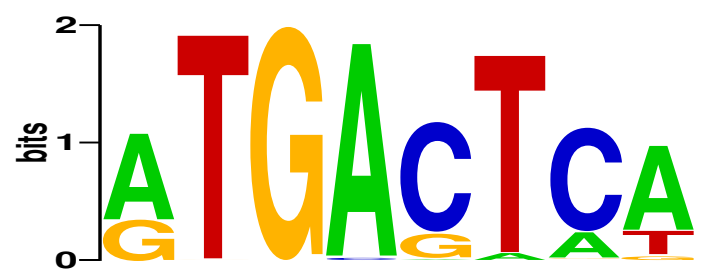	0	Yes
14	GCN4_SM	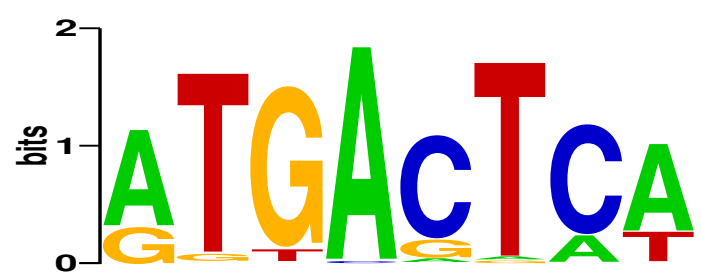	0	Yes
15	GCN4_YPD	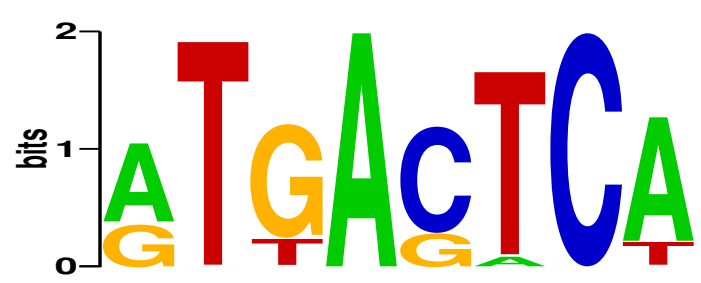	0	Yes
16	HAP1_YPD	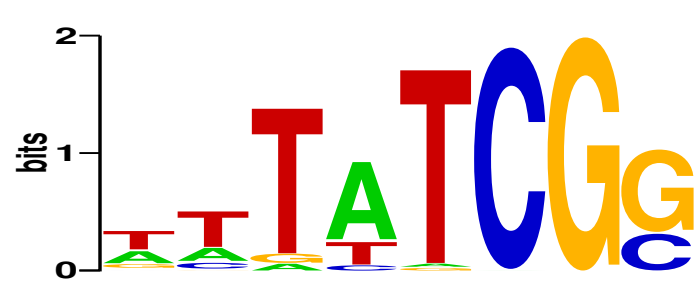	0	Yes
17	HAP4_YPD	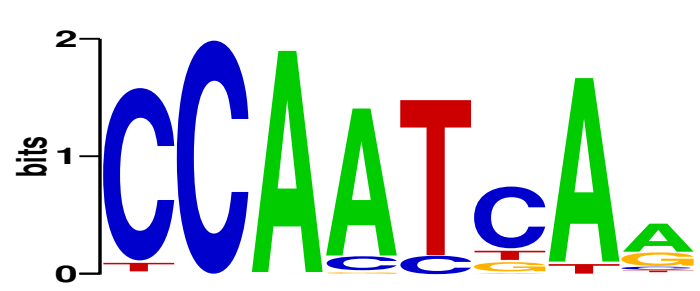	0	Yes
18	HSF1_H2O2Lo	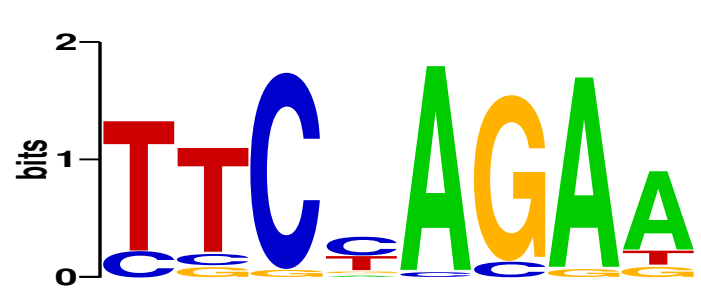	0	Yes
19	INO2_YPD	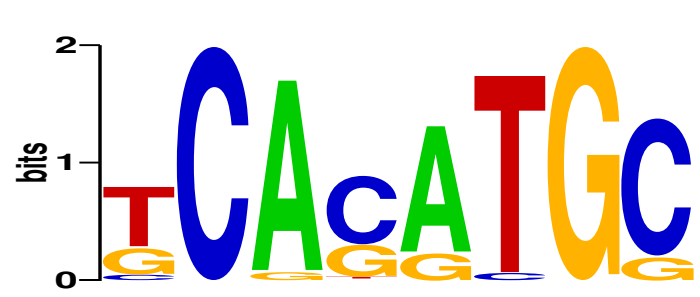	0	Yes
20	INO4_YPD	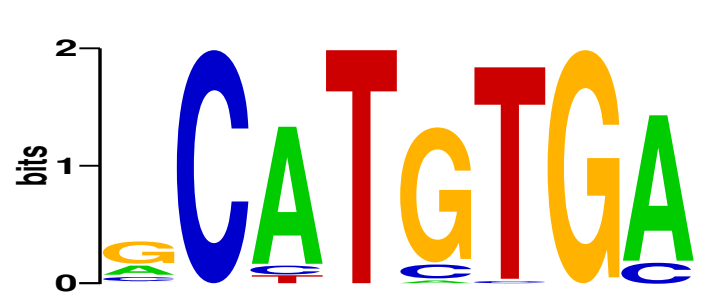	0	Yes

No.	Sequence-set	Motif	p-value	Match to literature
21	MBP1_H2O2Hi	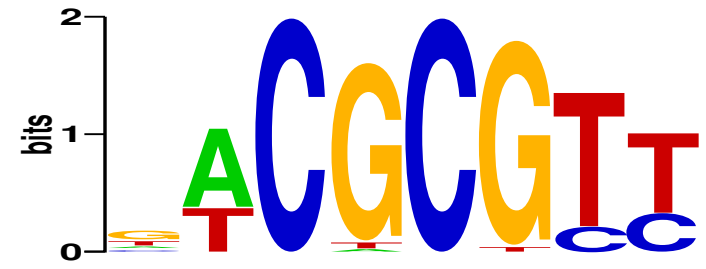	0	Yes
22	MBP1_H2O2Lo	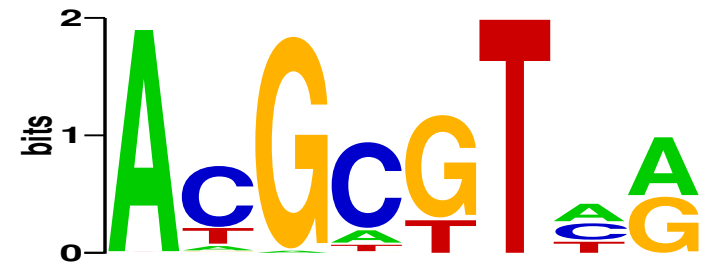	0	Yes
23	MBP1_YPD	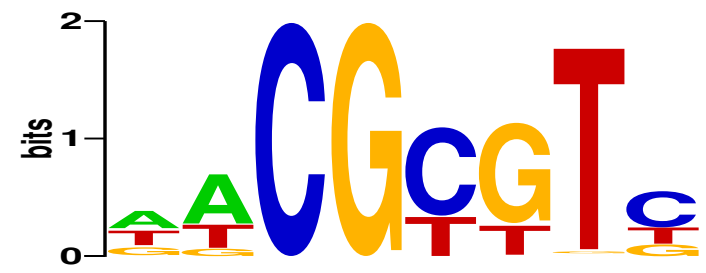	0	Yes
24	NRG1_H2O2Hi	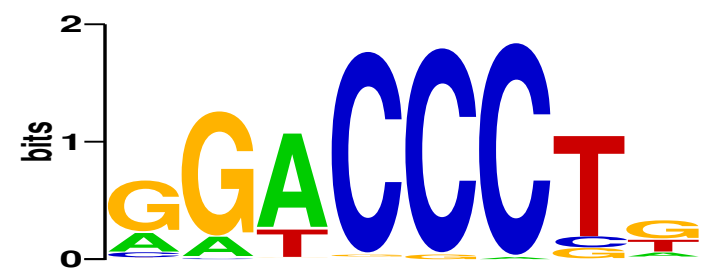	0	Yes
25	PDR1_YPD	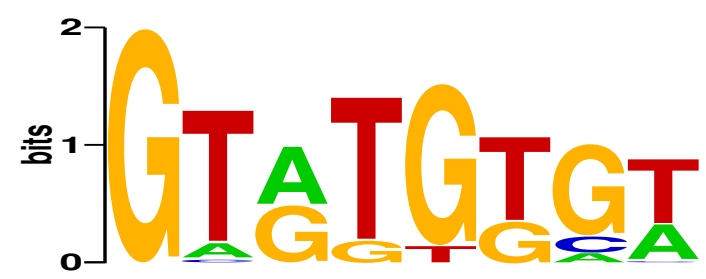	0	No*
26	RAP1_YPD	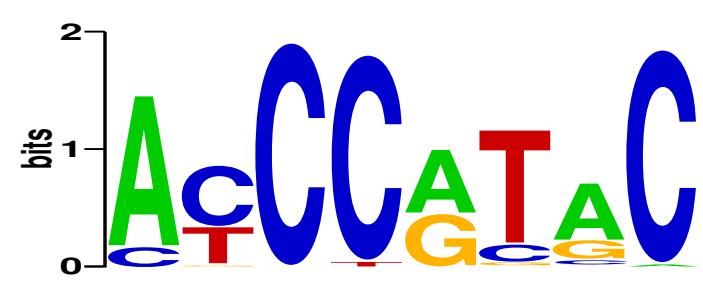	0	Yes
27	RCS1_H2O2Hi	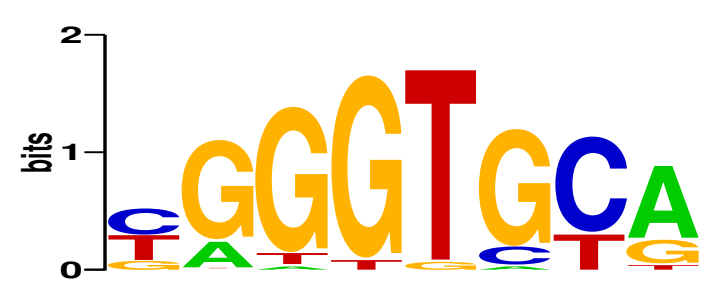	0	Yes
28	RCS1_H2O2Lo	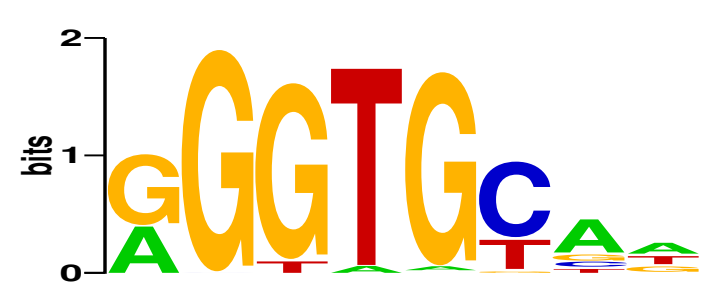	0	Yes
29	REB1_H2O2Hi	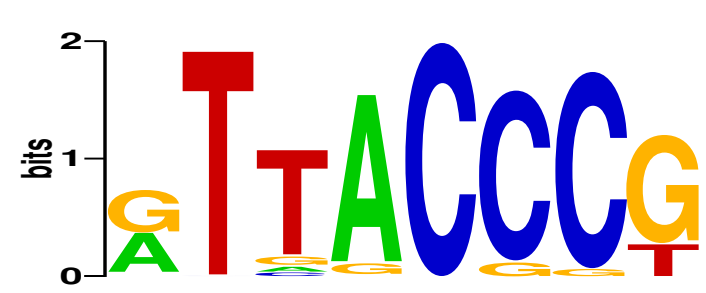	0	Yes
30	REB1_H2O2Lo	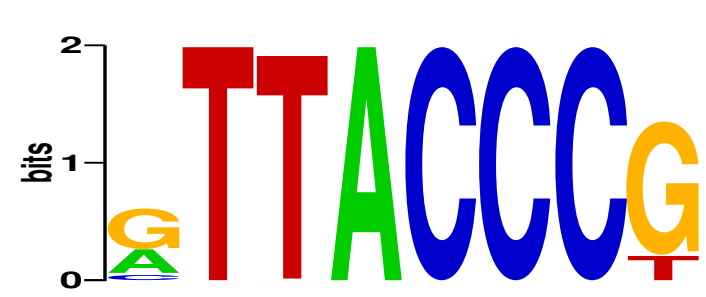	0	Yes

No.	Sequence-set	Motif	p-value	Match to literature
31	REB1_YPD	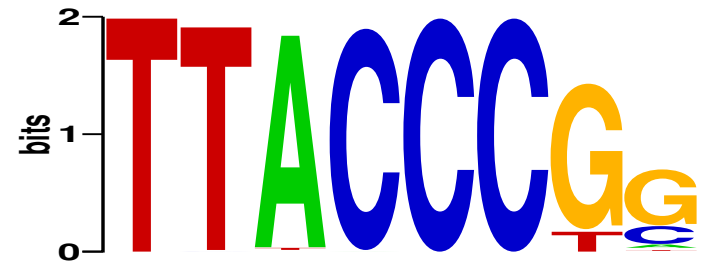	0	Yes
32	RPN4_H2O2Lo	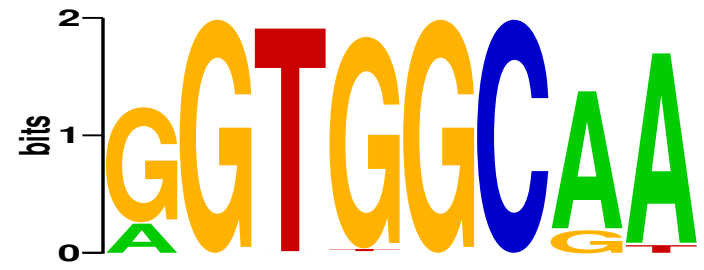	0	Yes
33	SKN7_H2O2Hi	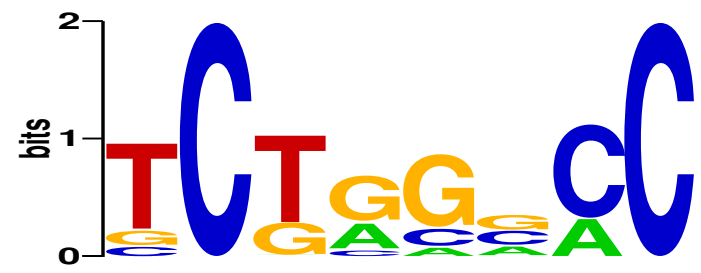	0	Yes
34	SKN7_H2O2Lo	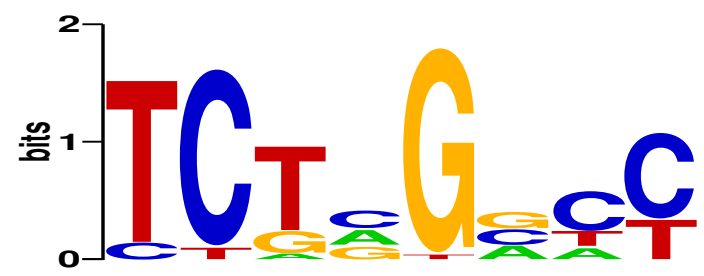	0	Yes
35	SKN7_YPD	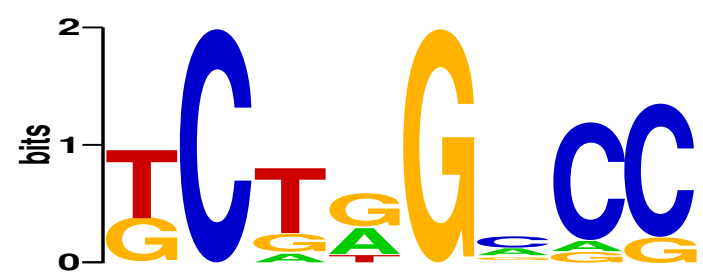	0	Yes
36	SUM1_YPD	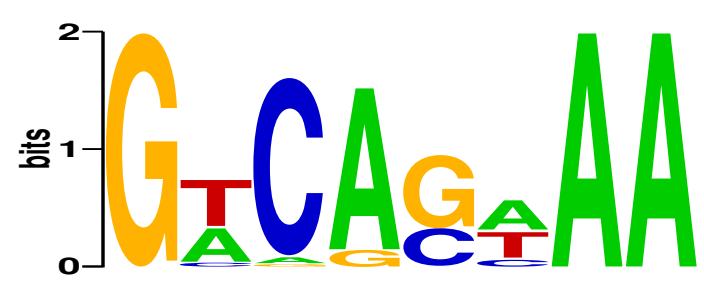	0	Yes
37	SWI4_YPD	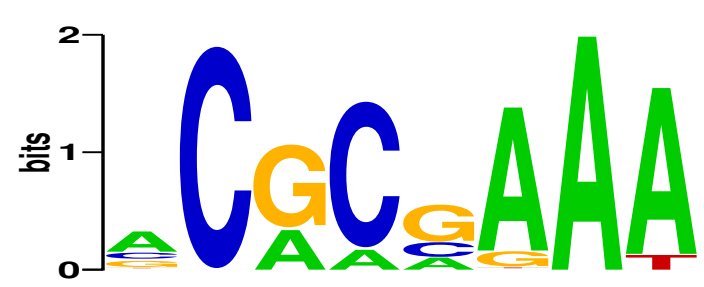	0	Yes
38	SWI6_YPD	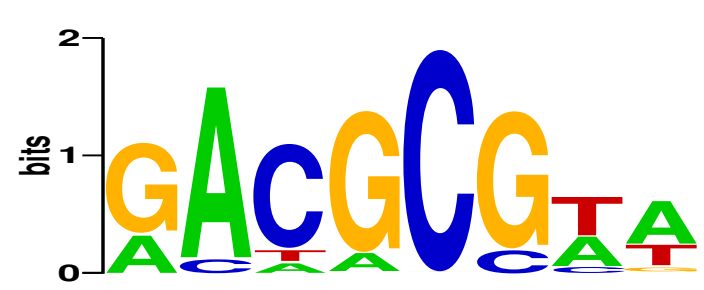	0	Yes
39	TYE7_YPD	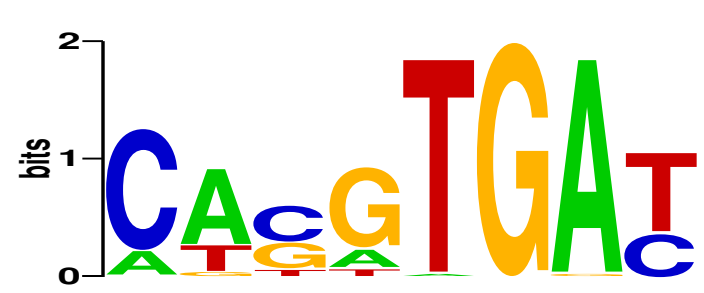	0	Yes
40	UME6_H2O2Hi	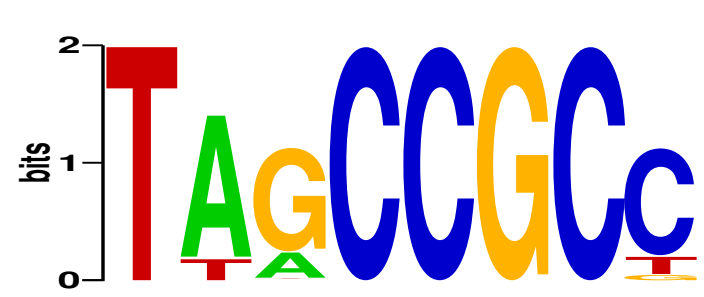	0	Yes

No.	Sequence-set	Motif	*p*-value	Match to literature
41	UME6_YPD	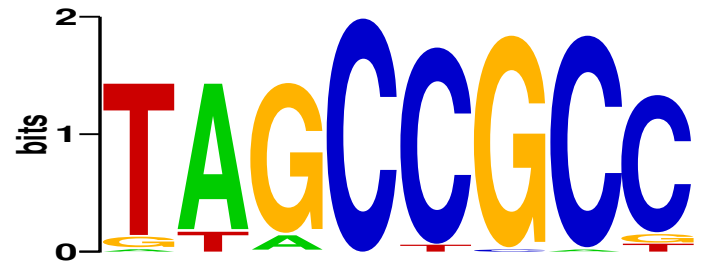	0	Yes
42	YAP7_H2O2Hi	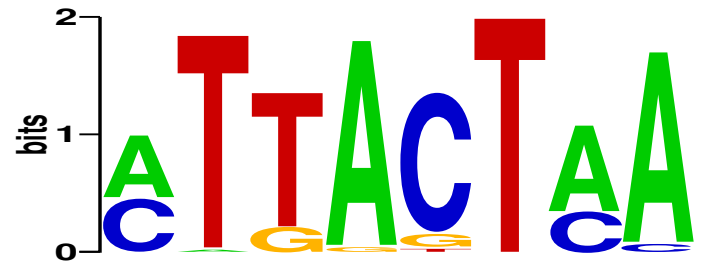	0	Yes
43	YAP7_H2O2Lo	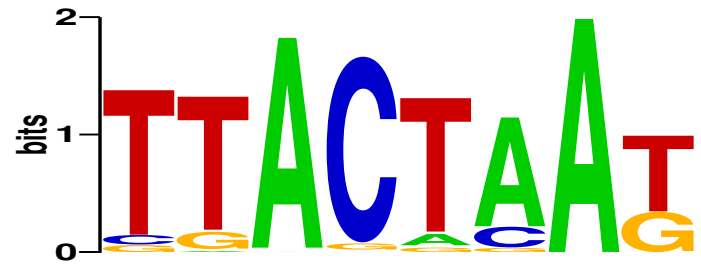	0	Yes
44	STE12_BUT14	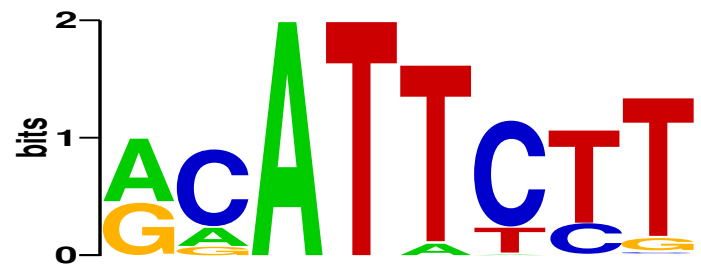	2.8866e-15	No**
45	MCM1_Alpha	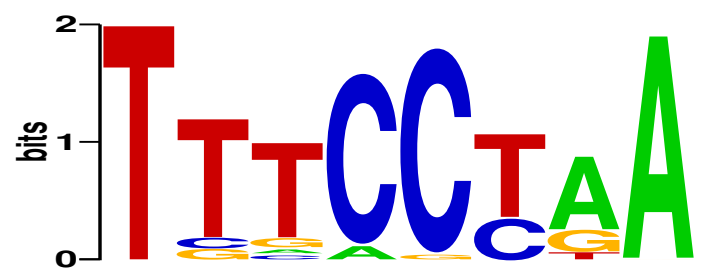	9.881e-15	Yes
46	STE12_Alpha	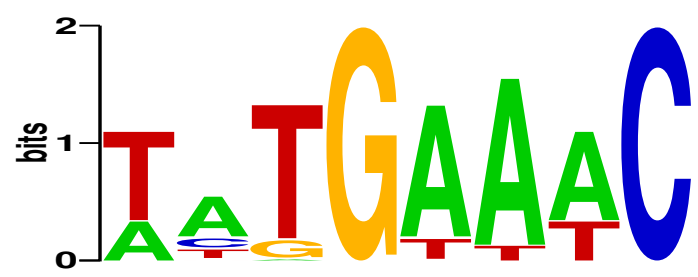	2.8977e-14	Yes
47	HSF1_H2O2Hi	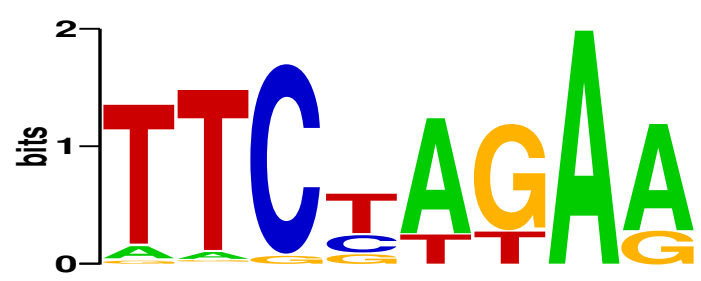	1.5909e-13	Yes
48	RTG3_H2O2Hi	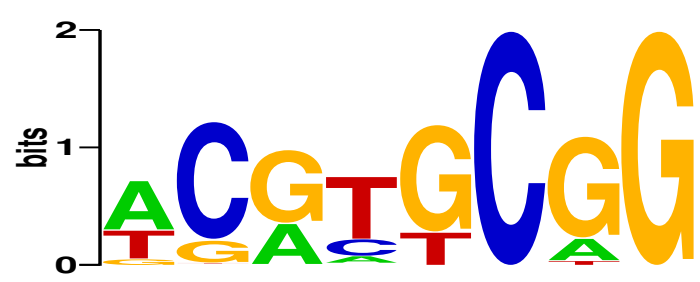	1.5569e-12	No
49	GAL4_YPD	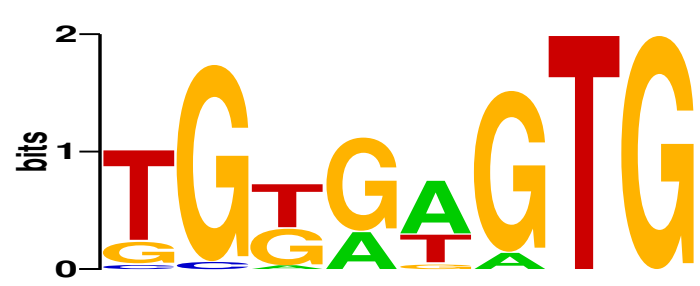	3.7348e-12	No*
50	PHO4_Pi-	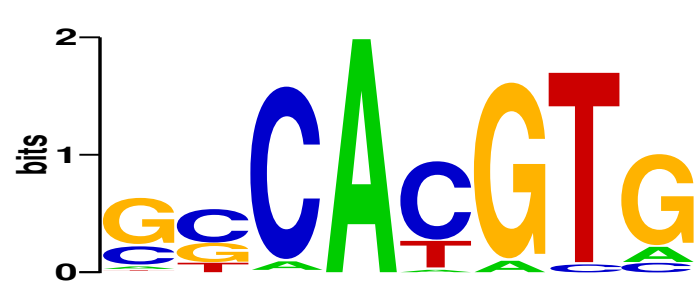	4.6896e-12	Yes

No.	Sequence-set	Motif	p-value	Match to literature
51	RPH1_H2O2Hi	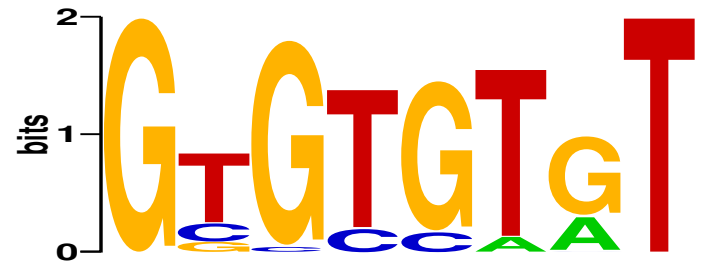	1.7833e-11	No*
52	ASH1_BUT14	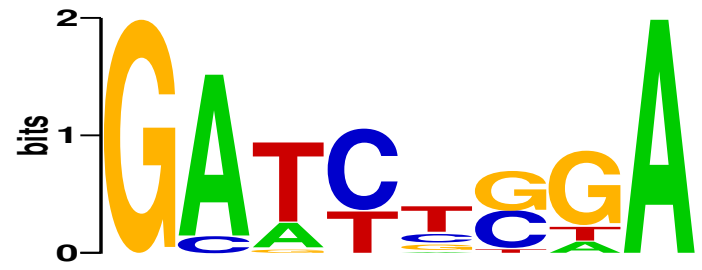	3.3913e-11	No
53	YAP1_H2O2Lo	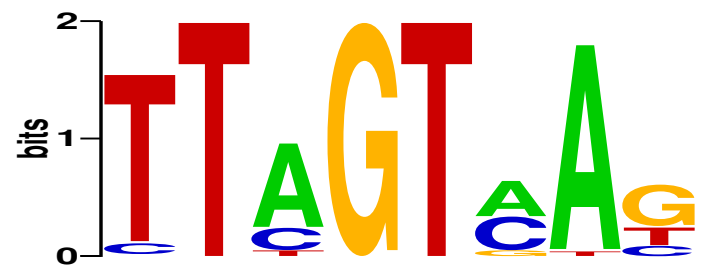	8.5543e-11	Yes
54	CIN5_H2O2Lo	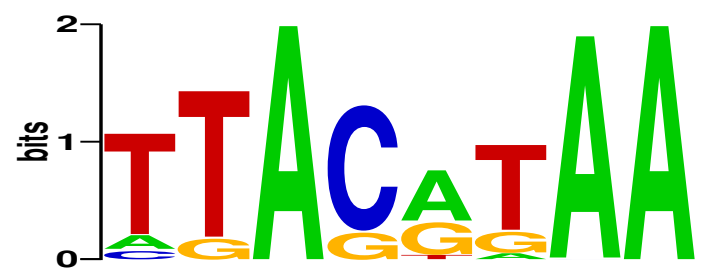	1.0909e-10	Yes
55	SKO1_YPD	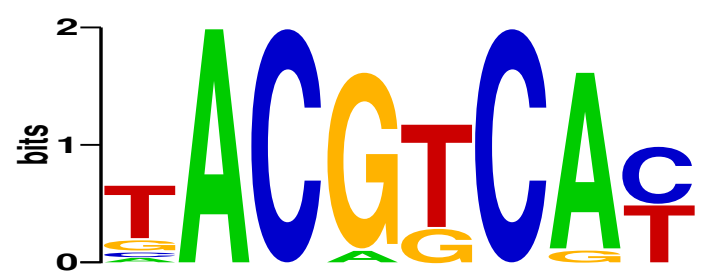	1.6359e-10	Yes
56	SMP1_YPD	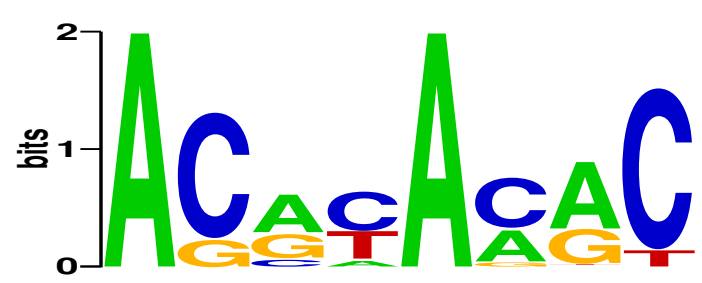	8.1032e-10	No*
57	BAS1_SM	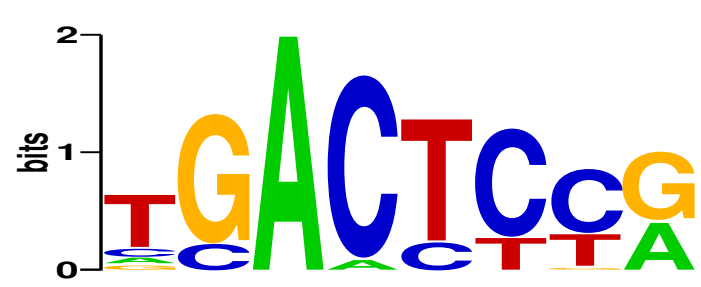	8.4863e-10	Yes
58	YAP5_YPD	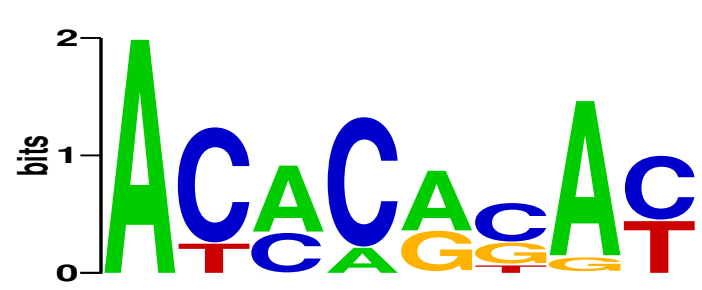	1.561e-09	No*
59	SWI5_YPD	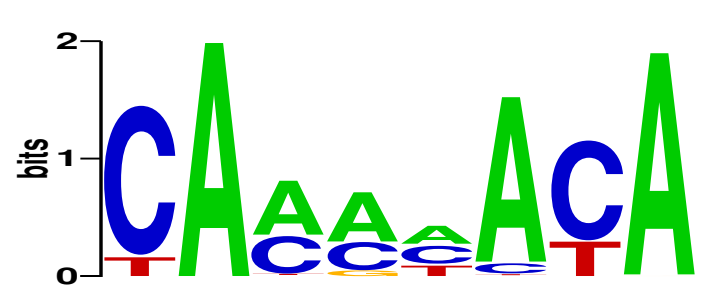	1.8089e-09	No*
60	NRG1_YPD	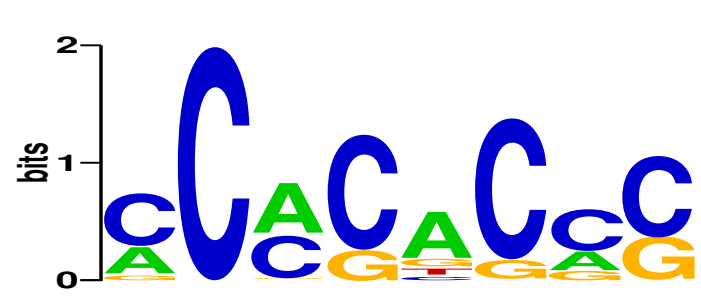	4.9181e-09	No

No.	Sequence-set	Motif	*p*-value	Match to literature
61	CAD1_YPD	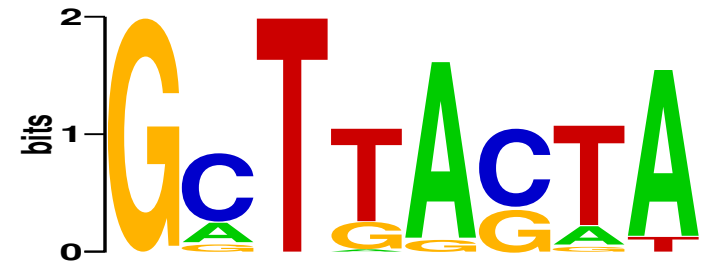	7.8107e-09	Yes
62	LEU3_SM	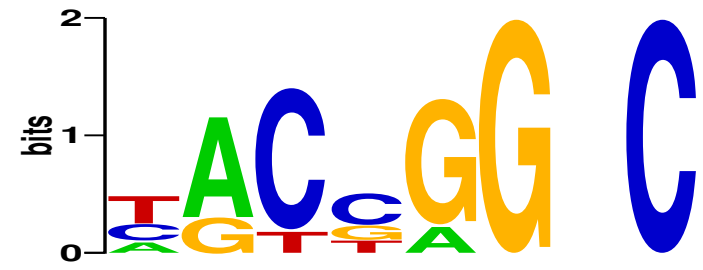	1.009e-08	Yes
63	SUT1_YPD	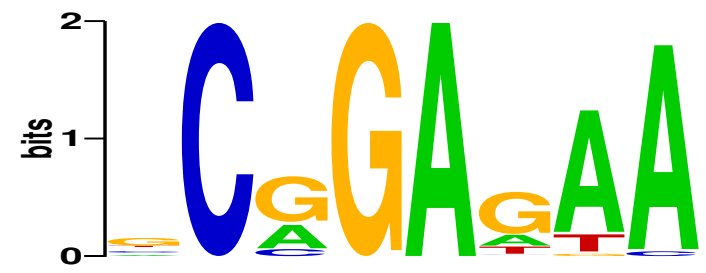	1.4191e-08	No
64	GLN3_YPD	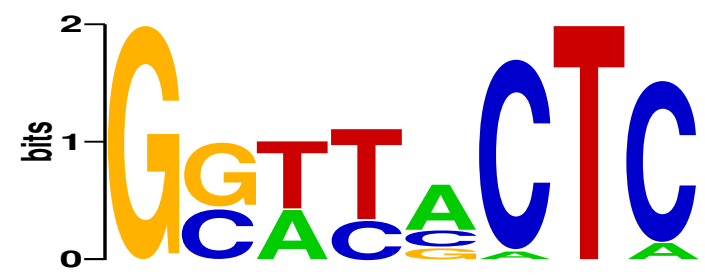	5.9425e-08	No
65	GLN3_RAPA	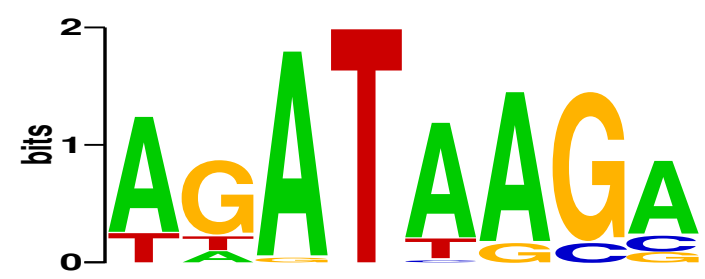	6.5648e-08	Yes
66	XBP1_H2O2Lo	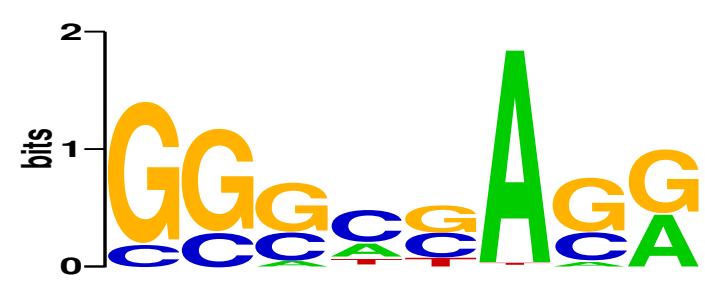	9.1032e-08	No
67	STB5_YPD	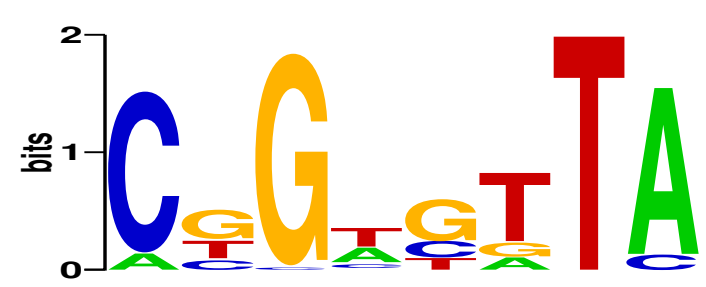	2.7928e-07	Yes
68	ARR1_YPD	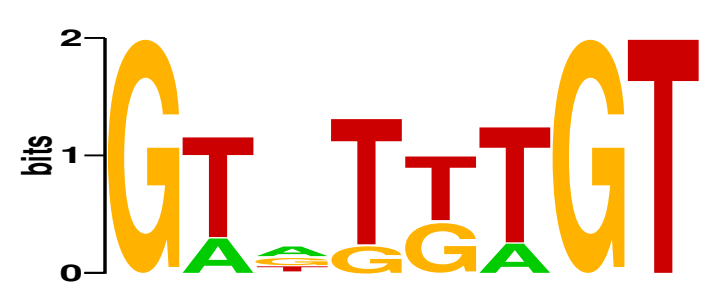	2.9071e-07	No*
69	STE12_YPD	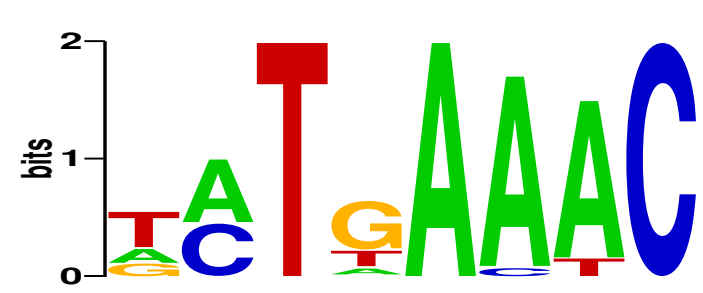	3.1672e-07	Yes
70	MET31_SM	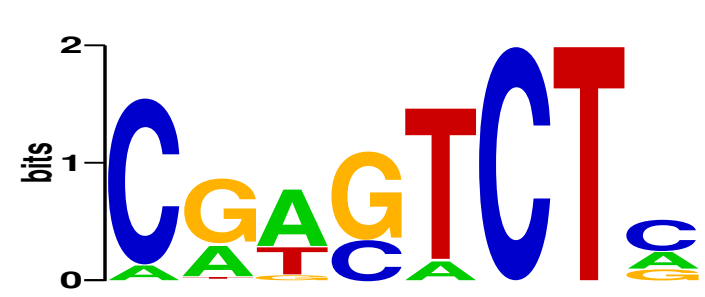	1.8285e-06	No

No.	Sequence-set	Motif	p-value	Match to literature
71	YAP1_YPD	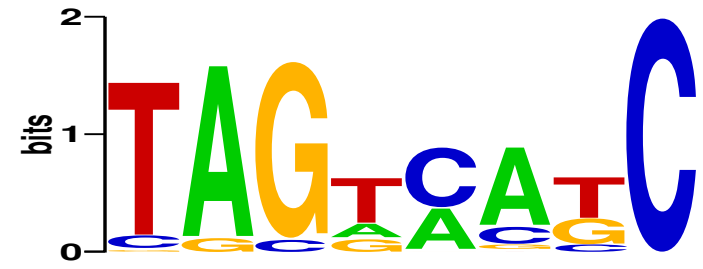	3.3232e-06	Yes
72	MET32_SM	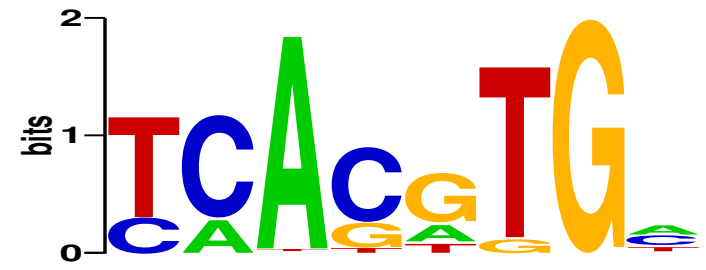	5.5417e-06	No
73	YAP6_H2O2Lo	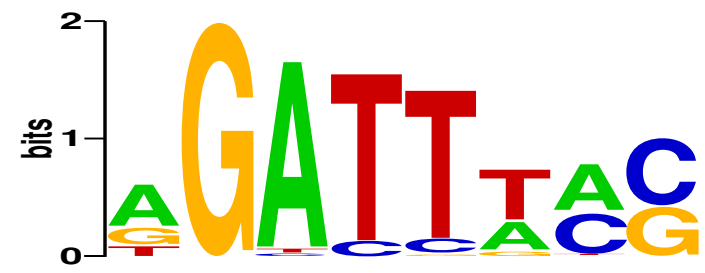	5.5572e-06	No
74	DAL82_YPD	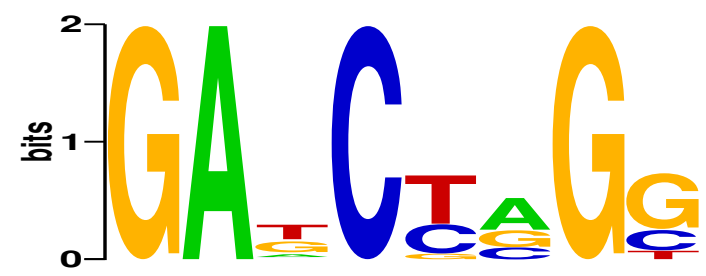	7.7969e-06	No
75	HAC1_YPD	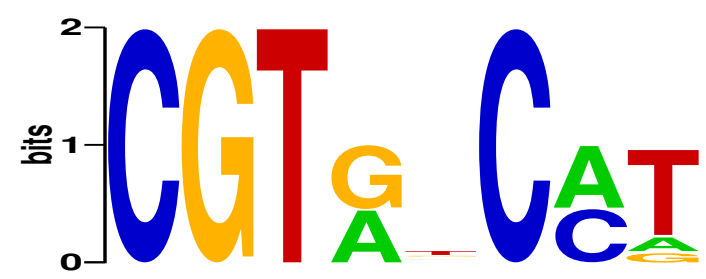	9.5379e-06	Yes
76	PUT3_YPD	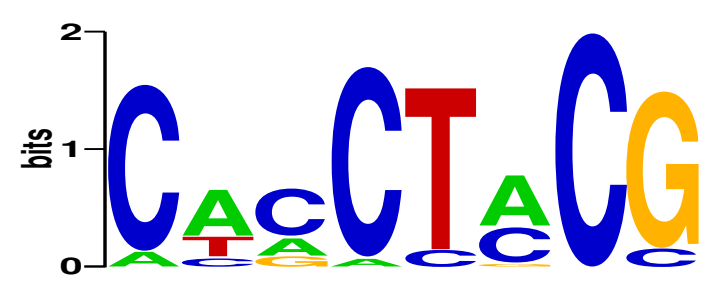	1.176e-05	No
77	MET32_YPD	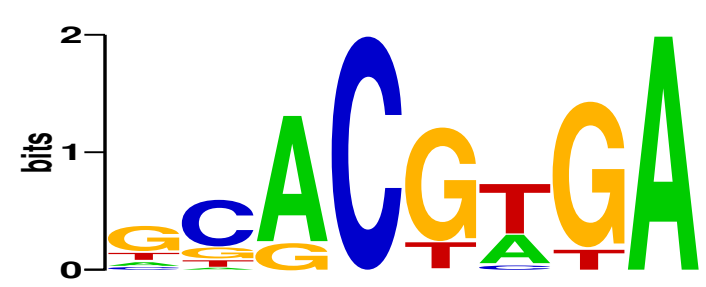	1.4597e-05	No
78	ROX1_H2O2Hi	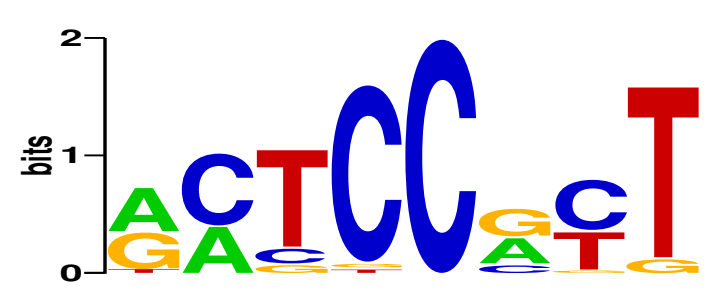	1.5344e-05	No
79	STE12_BUT90	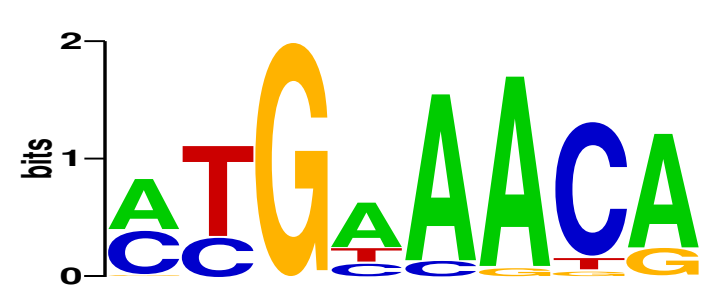	1.5651e-05	Yes
80	GLN3_SM	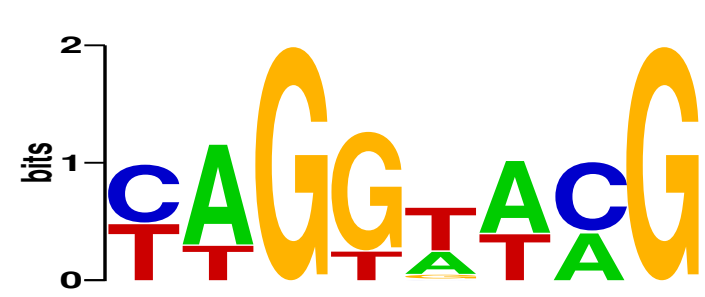	1.5837e-05	No

No.	Sequence-set	Motif	p-value	Match to literature
81	FKH2_H2O2Lo	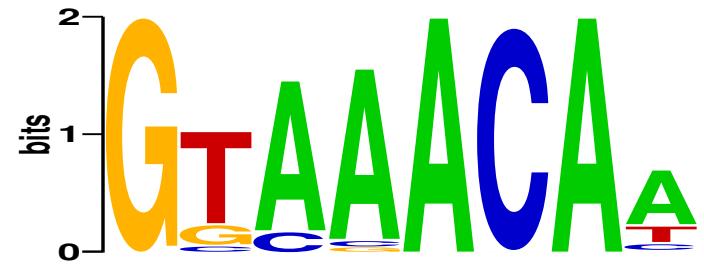	2.9307e-05	Yes
82	HAP5_SM	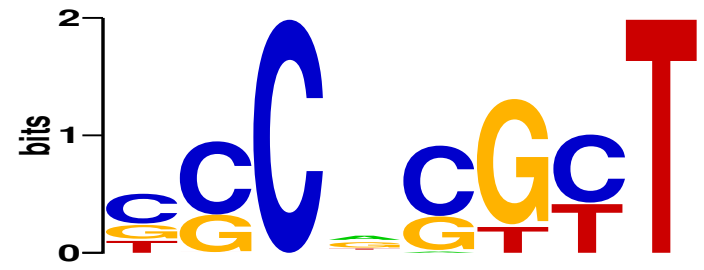	3.894e-05	No
83	CIN5_H2O2Hi	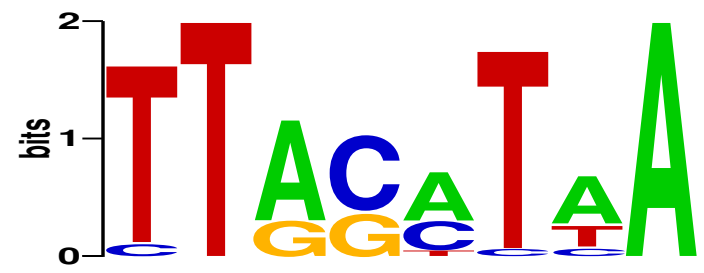	4.7852e-05	Yes
84	MSN2_H2O2Lo	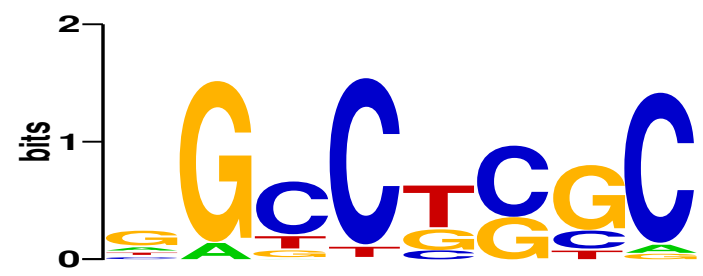	5.6921e-05	No
85	MSN2_Acid	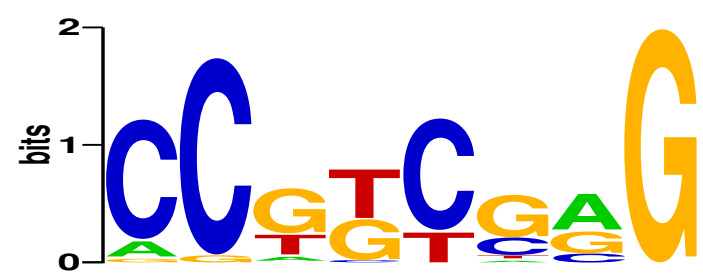	6.071e-05	No
86	MAC1_YPD	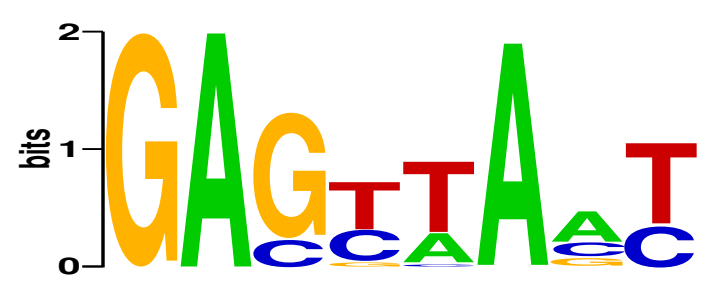	9.3389e-05	Yes
87	TEC1_Alpha	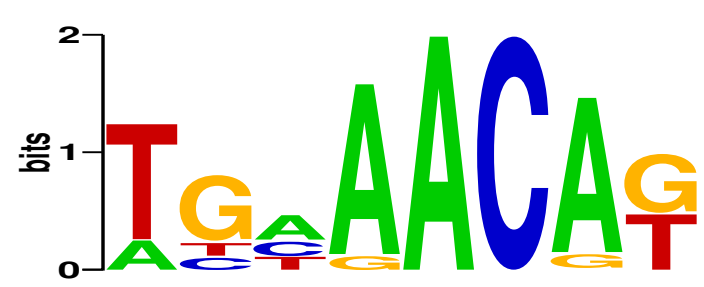	0.00015144	No**
88	TEC1_BUT14	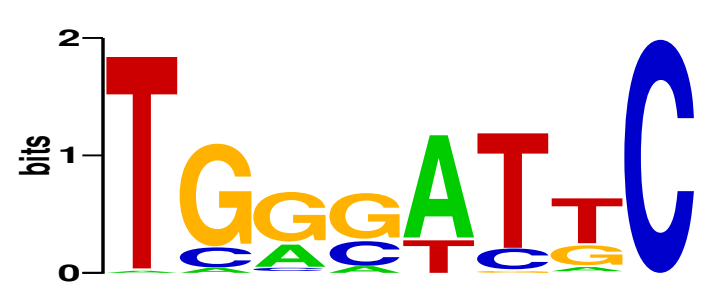	0.00015453	No
89	RPH1_YPD	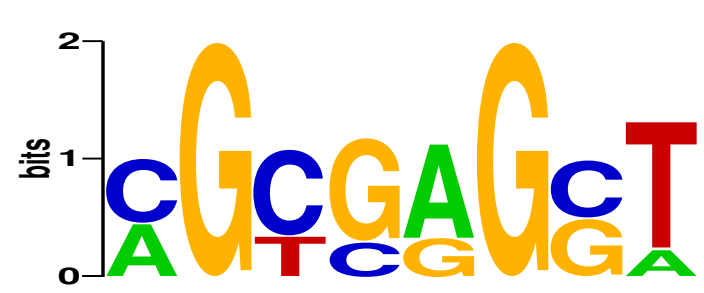	0.00019026	No
90	GCR1_YPD	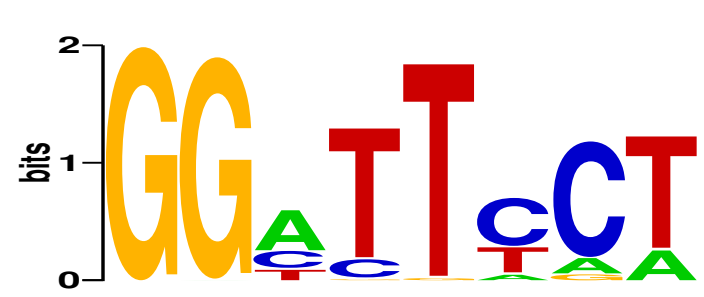	0.00022014	Yes

No.	Sequence-set	Motif	p-value	Match to literature
91	LEU3_YPD	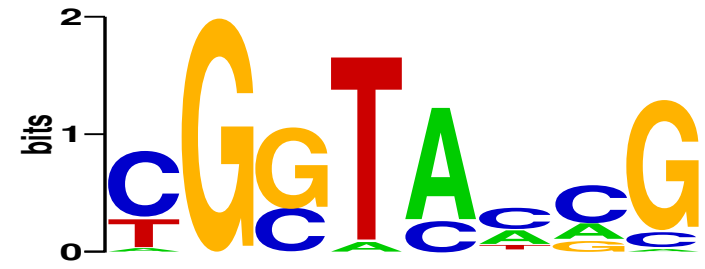	0.00022692	Yes
92	HAP3_YPD	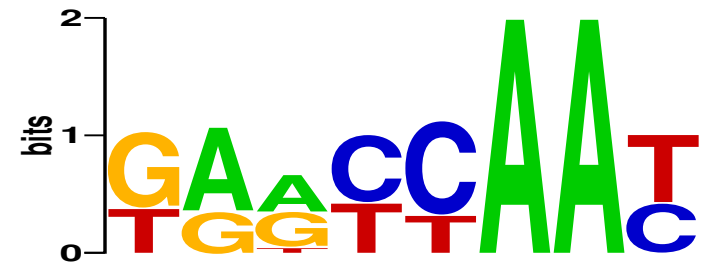	0.00024498	Yes
93	DAL82_SM	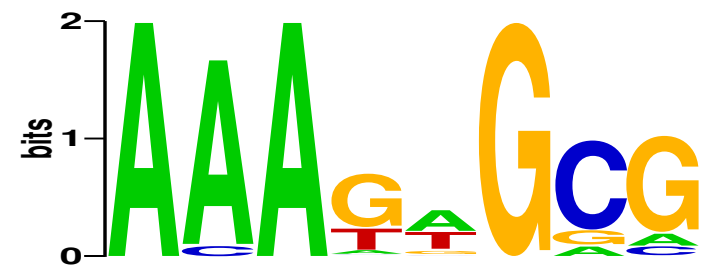	0.00043324	Yes
94	DAL82_RAPA	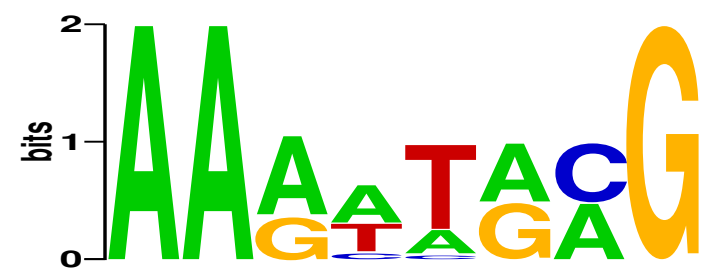	0.00052712	Yes
95	RTG3_RAPA	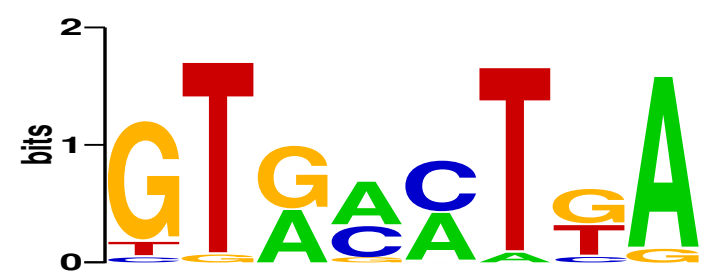	0.00059191	No
96	MSN2_H2O2Hi	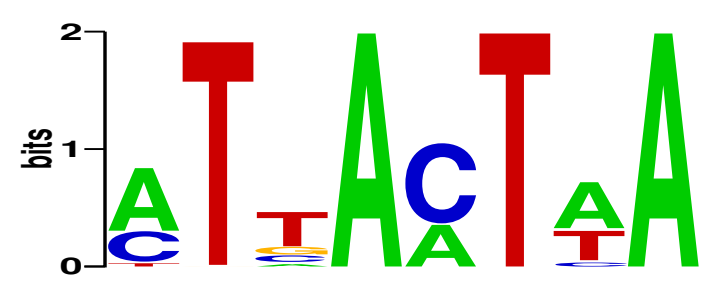	0.00067082	No
97	GZF3_RAPA	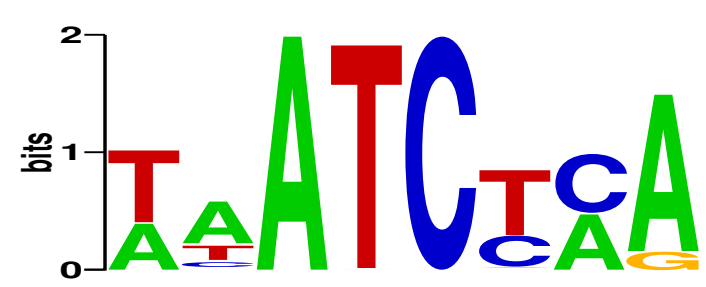	0.00071649	No
98	MSN4_YPD	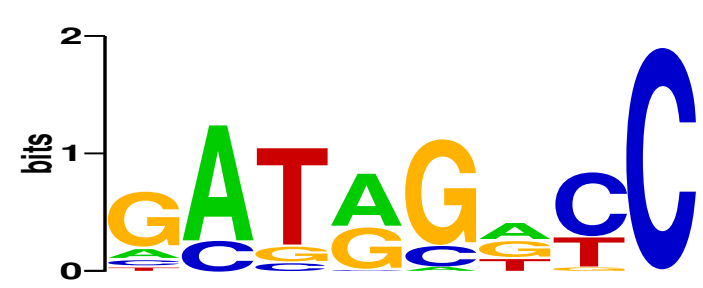	0.00098603	No
99	NRG1_H2O2Lo	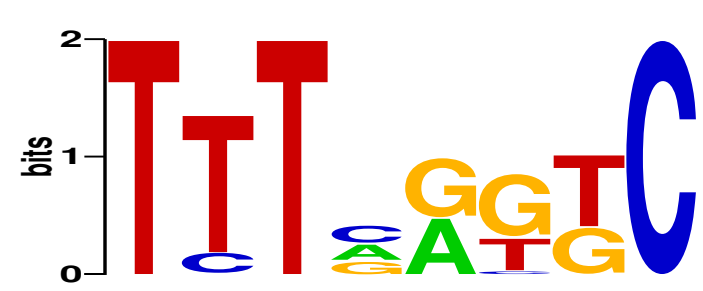	0.0010865	No
100	STP1_YPD	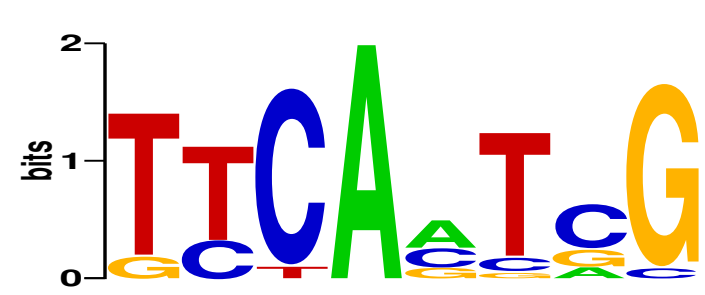	0.0012159	No

No.	Sequence-set	Motif	p-value	Match to literature
101	YAP6_H2O2Hi	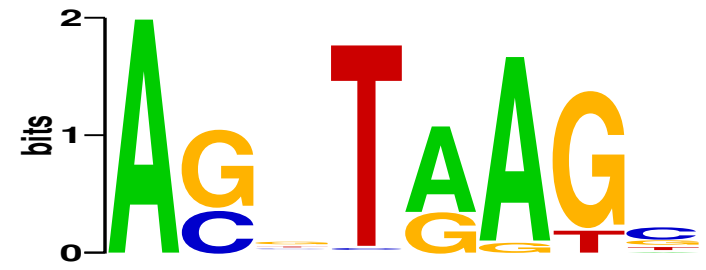	0.0012971	No
102	GAT1_RAPA	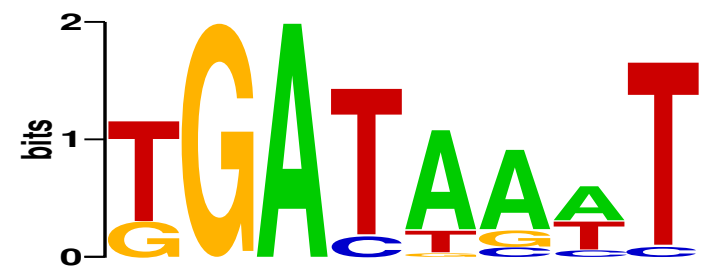	0.0014653	Yes
103	RTG1_SM	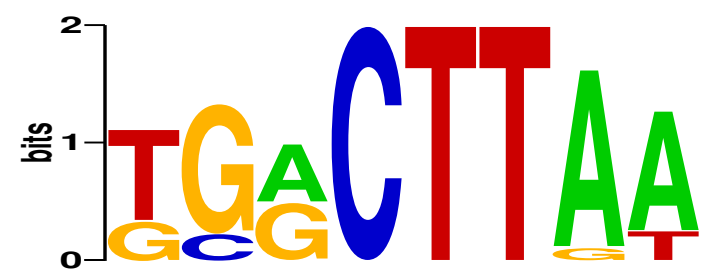	0.0014687	No
104	RPH1_SM	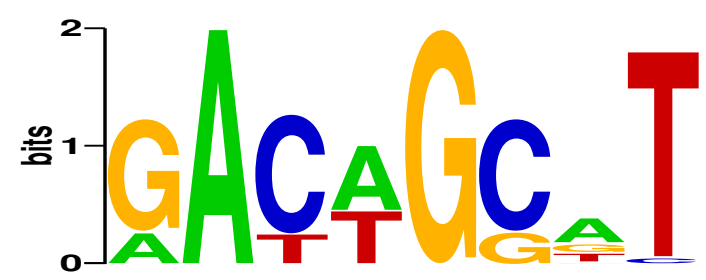	0.0015045	No
105	ADR1_YPD	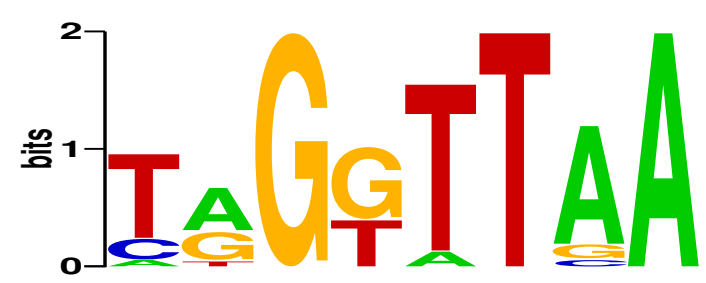	0.0015052	No
106	UGA3_SM	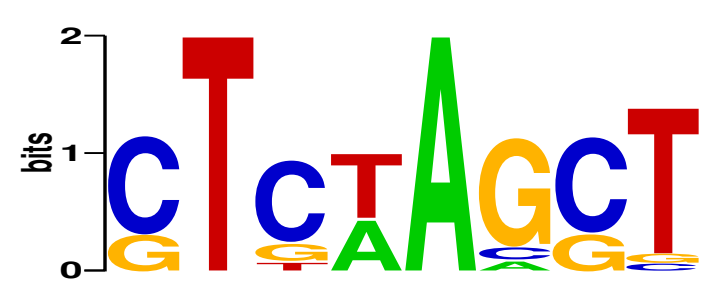	0.0015247	No
107	RTG3_YPD	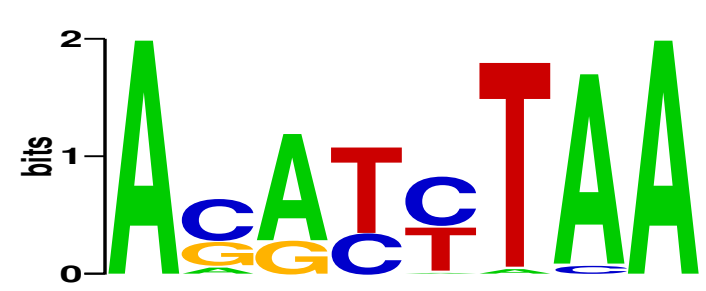	0.0015802	No
108	HAP2_YPD	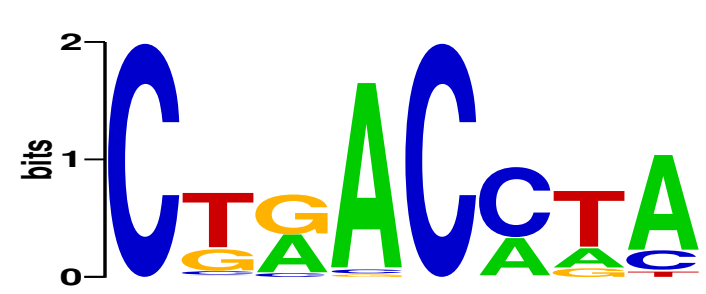	0.0026357	No
109	YOX1_YPD	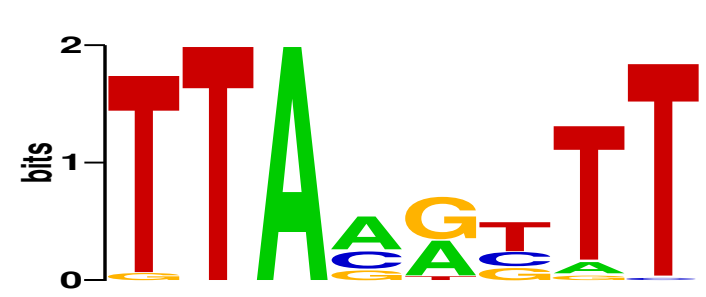	0.0028295	No
110	RPN4_YPD	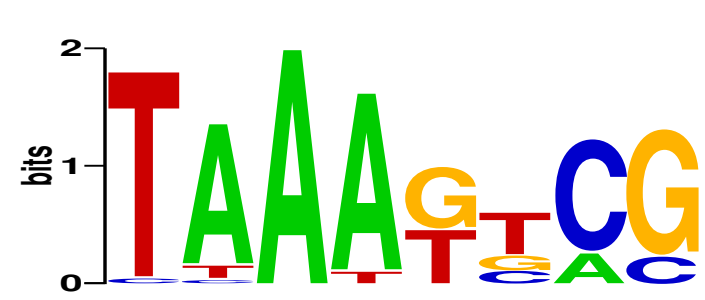	0.0028328	No

No.	Sequence-set	Motif	p-value	Match to literature
111	CAD1_SM	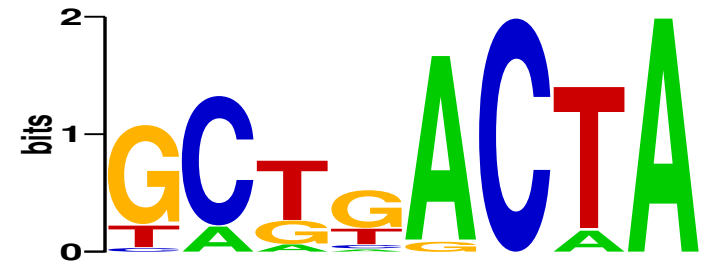	0.0030512	Yes
112	MET31_YPD	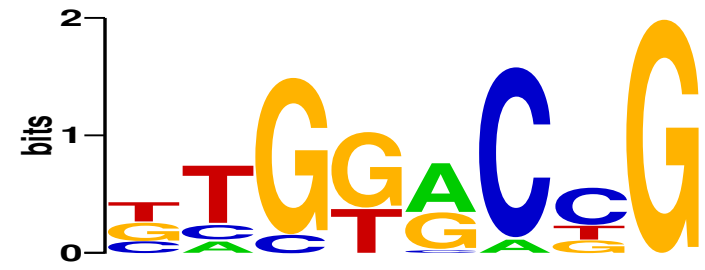	0.004237	No
113	HAP2_RAPA	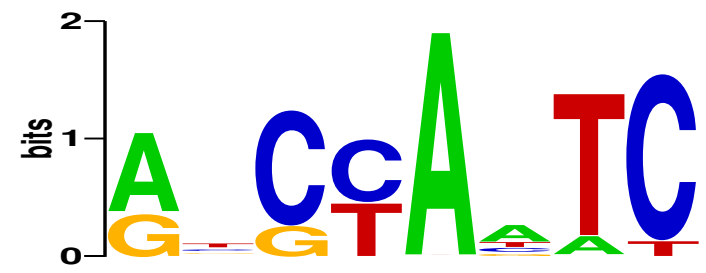	0.0052141	Yes
114	MSN4_H2O2Hi	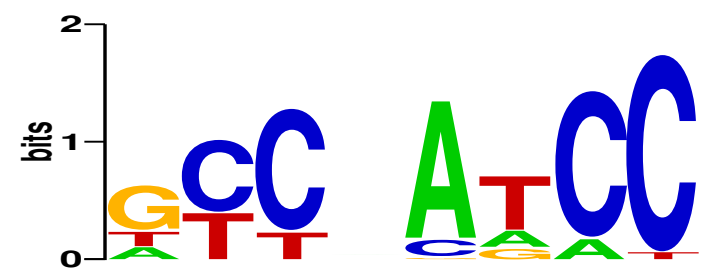	0.0055192	No
115	TEC1_YPD	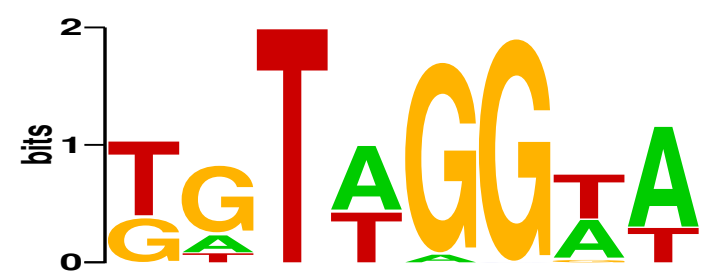	0.005915	No
116	DAL80_RAPA	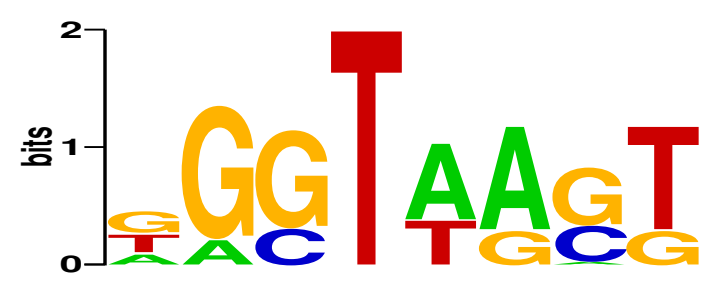	0.0060038	No
117	MOT3_YPD	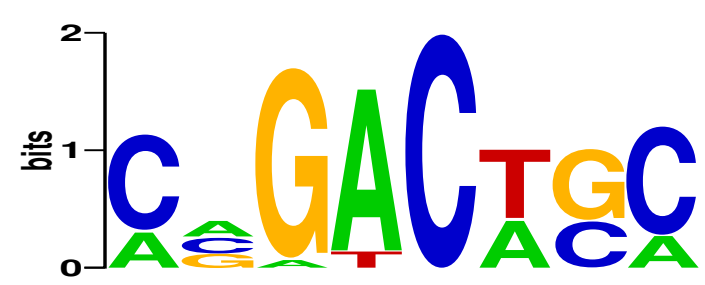	0.0063403	No
118	MSN4_H2O2Lo	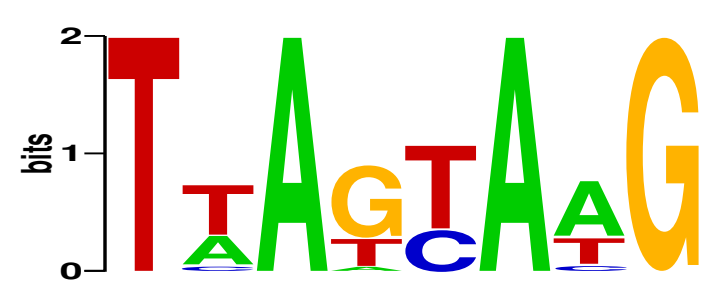	0.0078024	No
119	GAL4_RAFF	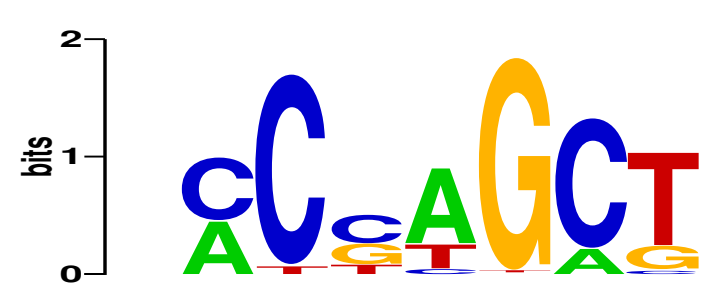	0.010915	No
120	UGA3_RAPA	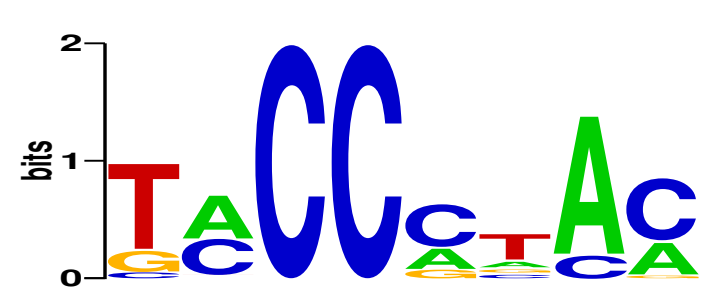	0.012632	No

No.	Sequence-set	Motif	p-value	Match to literature
121	RTG3_SM	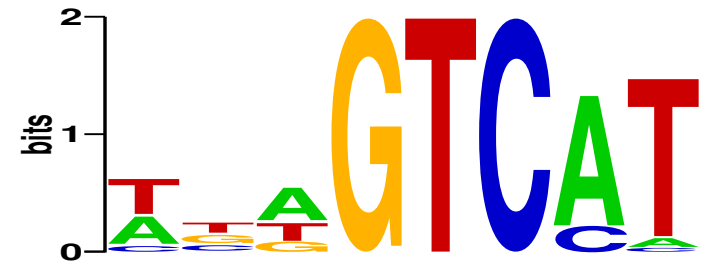	0.014349	No
122	AZF1_YPD	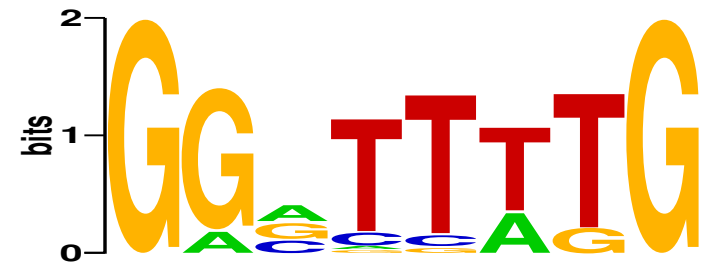	0.016029	No
123	MCM1_YPD	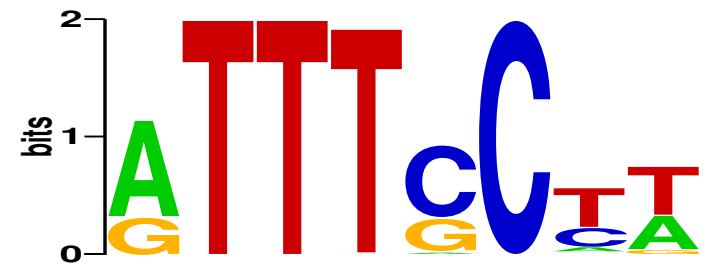	0.018747	Yes
124	ROX1_YPD	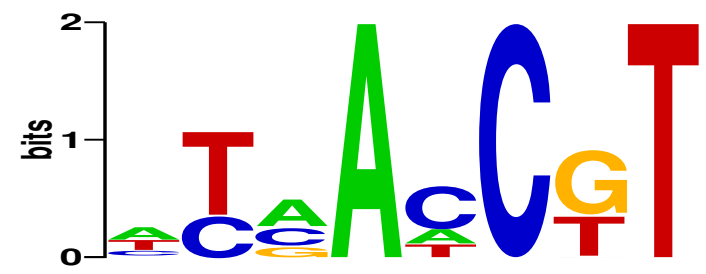	0.023021	No
125	RLM1_YPD	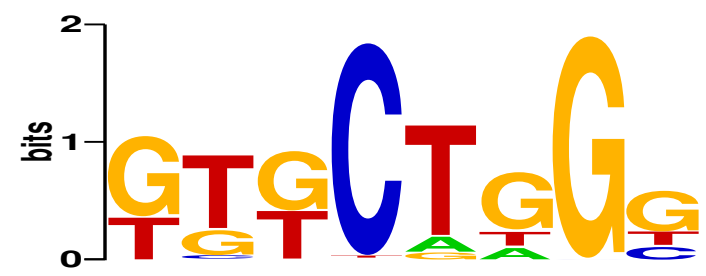	0.023389	No
126	ROX1_H2O2Lo	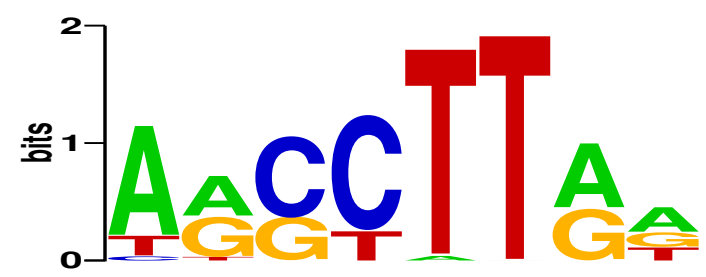	0.024433	No
127	MOT3_SM	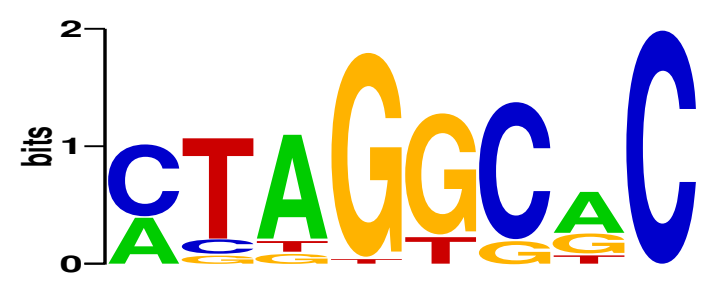	0.024816	No
128	MAC1_H2O2Hi	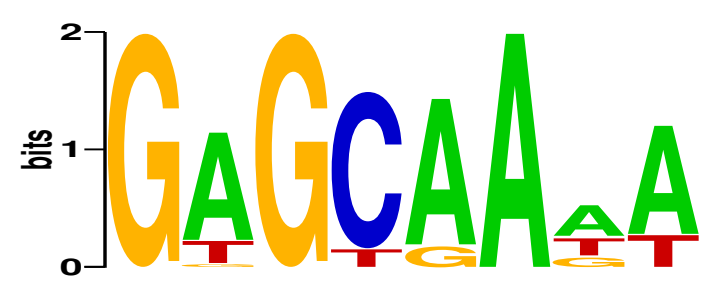	0.025741	Yes
129	AFT2_YPD	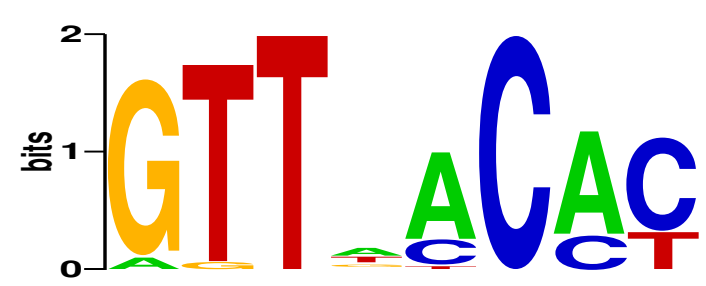	0.026932	No
130	YAP6_YPD	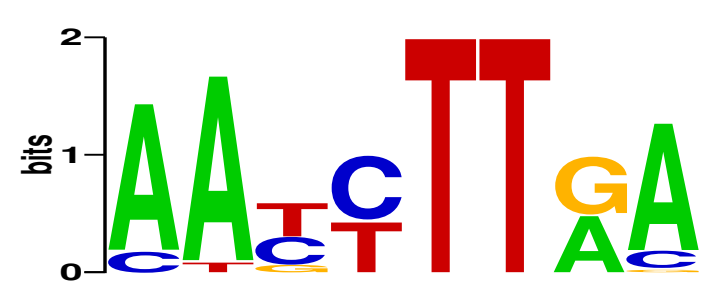	0.026955	No

No.	Sequence-set	Motif	p-value	Match to literature
131	DAL81_YPD	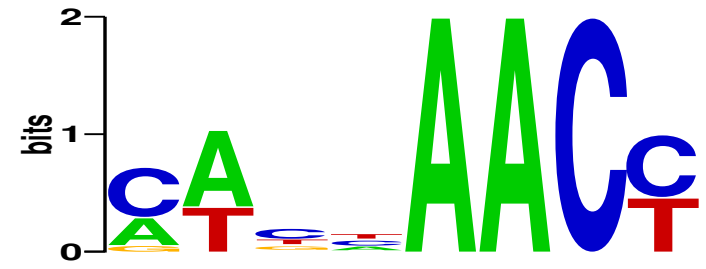	0.02843	No
132	YAP3_YPD	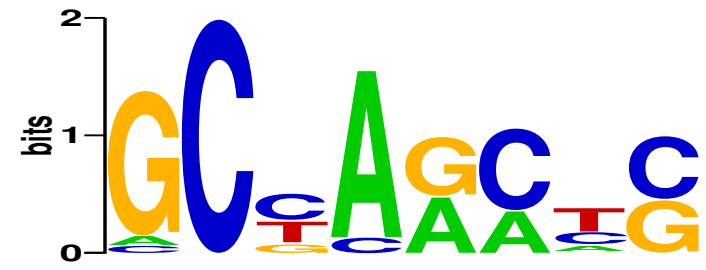	0.03141	No
133	PHO4_YPD	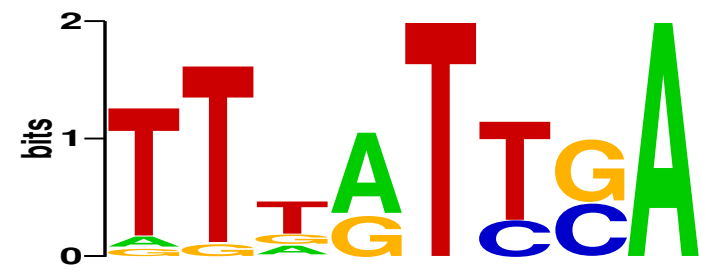	0.049087	No
134	RCS1_SM	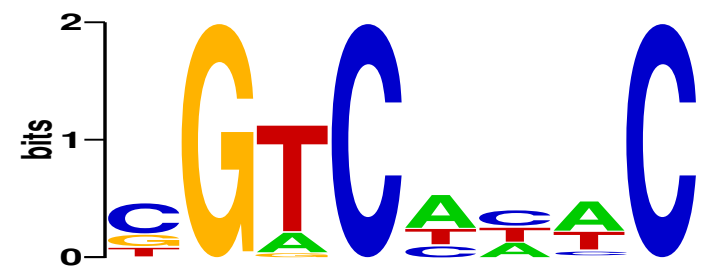	0.060689	No
135	RIM101_H2O2Hi	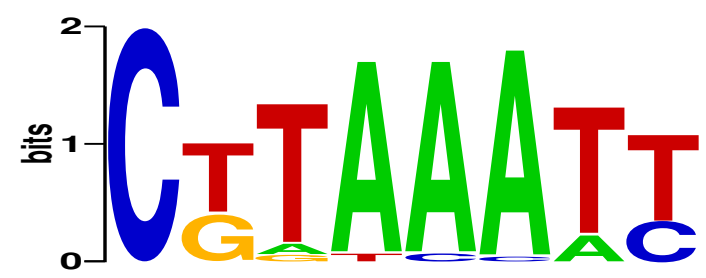	0.076127	No
136	PDR3_YPD	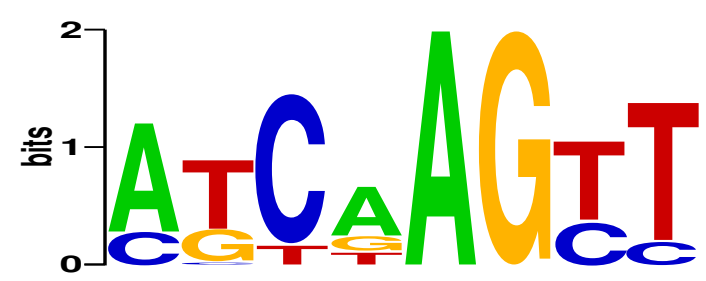	0.077626	No
137	GAL4_GAL	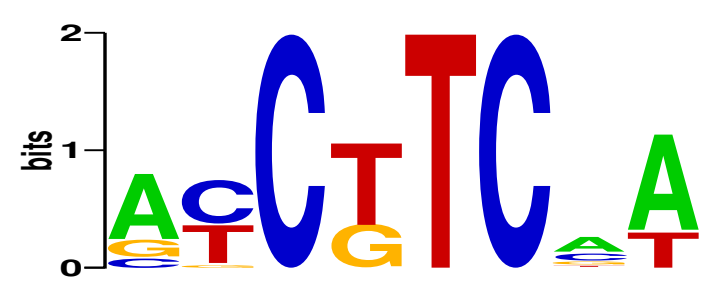	0.079778	No
138	RTG1_RAPA	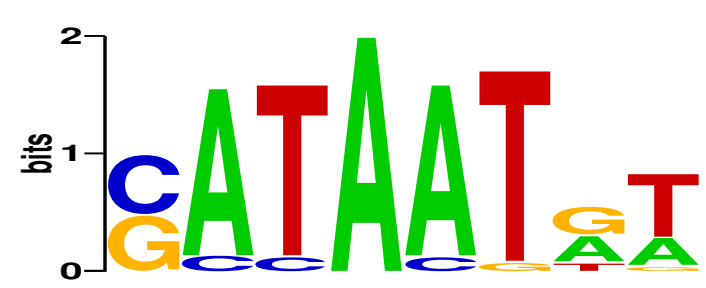	0.081778	No
139	HAP4_H2O2Lo	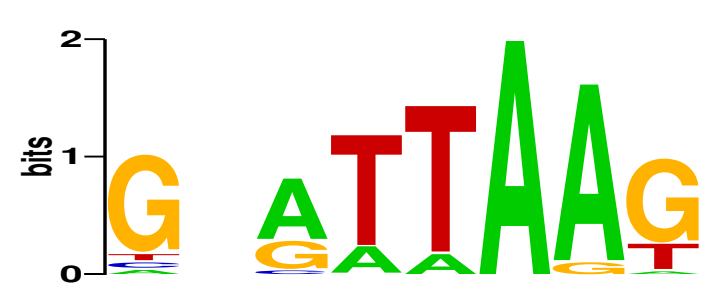	0.083529	No
140	MSN2_RAPA	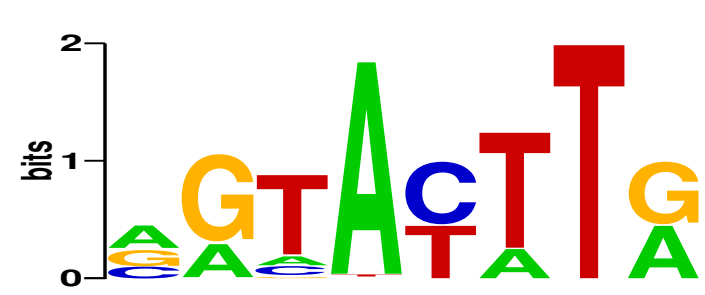	0.084886	No

No.	Sequence-set	Motif	p-value	Match to literature
141	GZF3_H2O2Hi	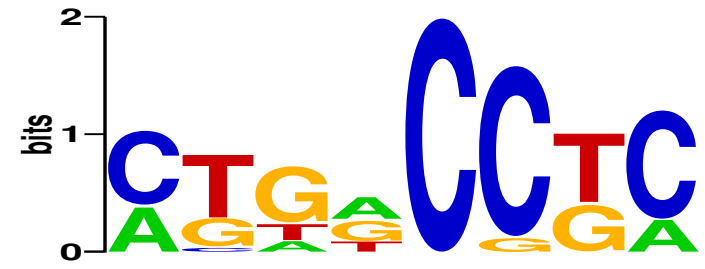	0.11514	No
142	RCS1_YPD	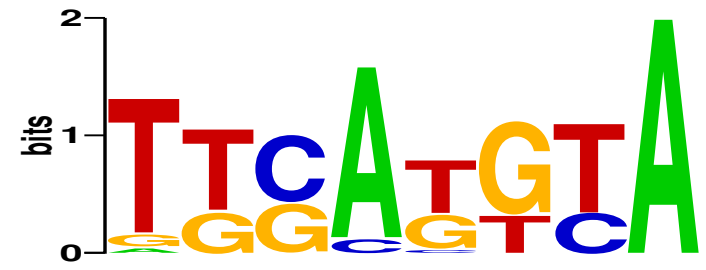	0.11816	No
143	GAT1_SM	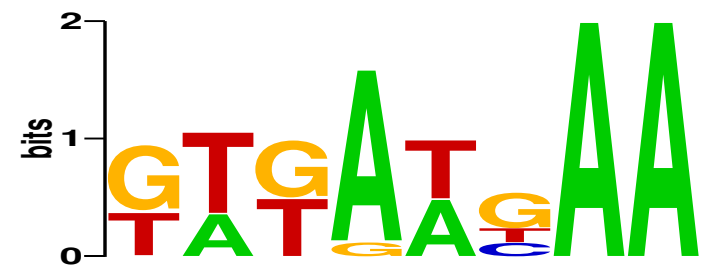	0.13297	No
144	STP1_SM	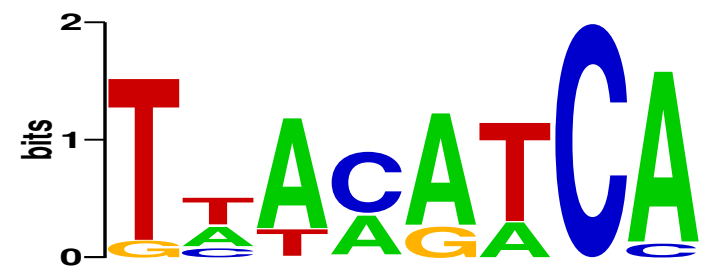	0.13453	No
145	PUT3_SM	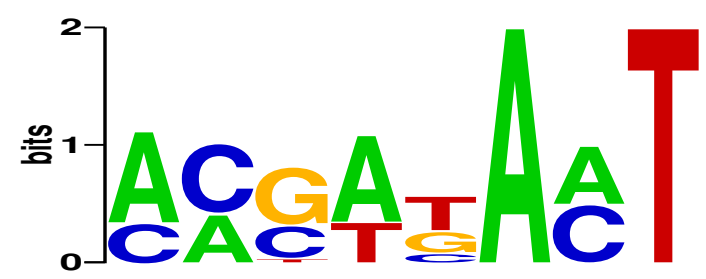	0.13613	No
146	DAL80_YPD	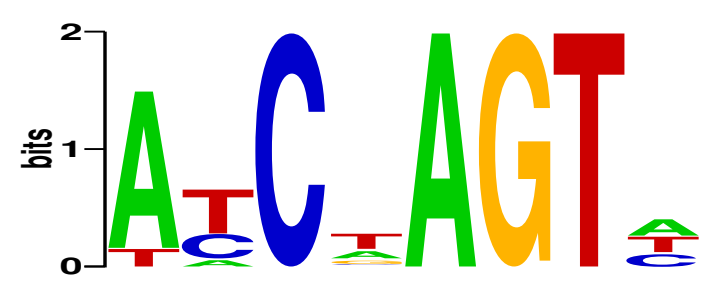	0.17524	No
147	ADR1_HEAT	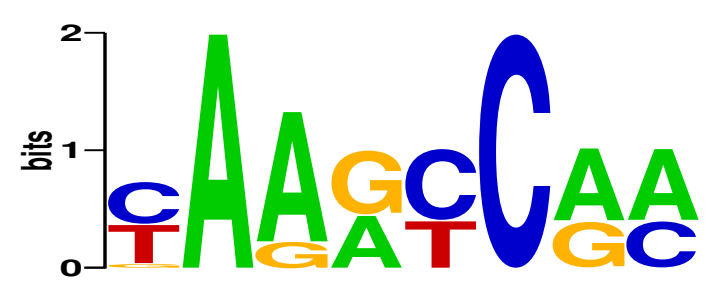	0.20828	No
148	ZAP1_YPD	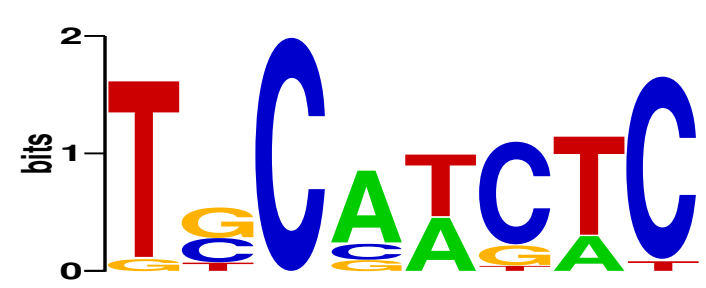	0.21862	No
149	MSN4_Acid	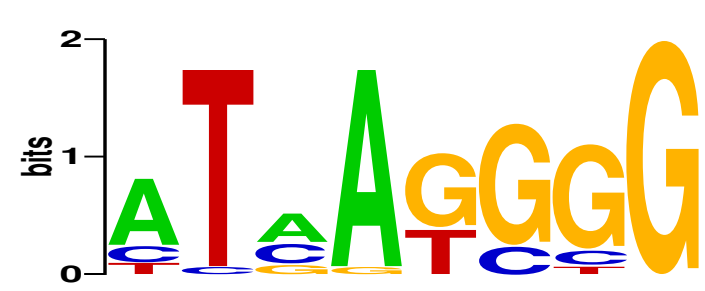	0.22329	Yes
150	YHP1_YPD	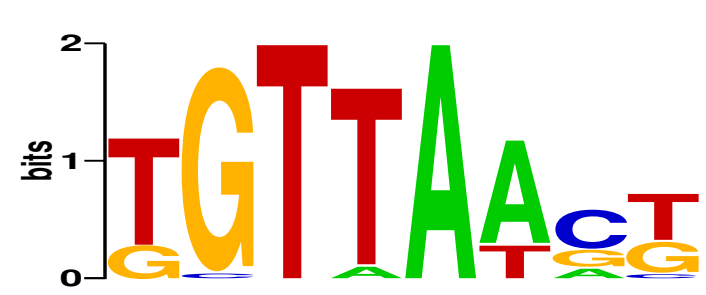	0.24397	No

No.	Sequence-set	Motif	p-value	Match to literature
151	SIP4_SM	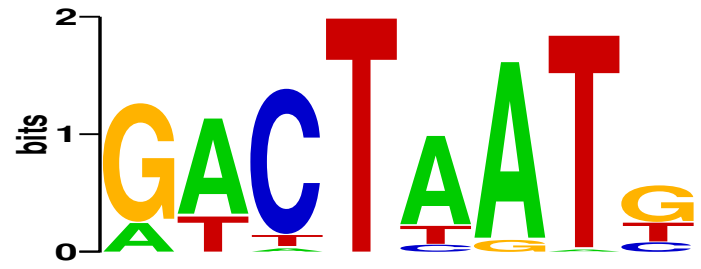	0.26483	No
152	SIP4_YPD	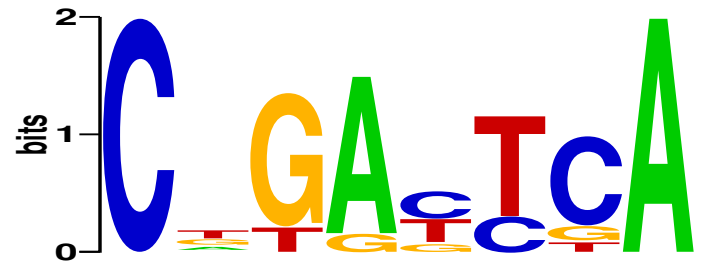	0.26673	No
153	HAP5_YPD	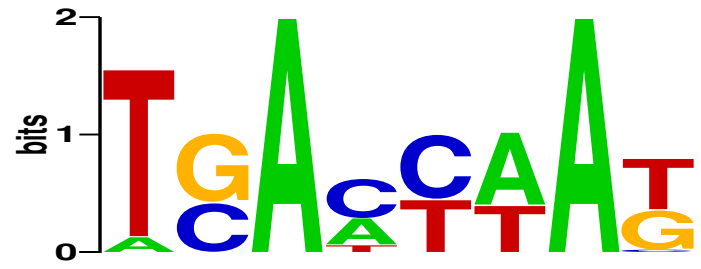	0.2957	No
154	PDR1_H2O2Lo	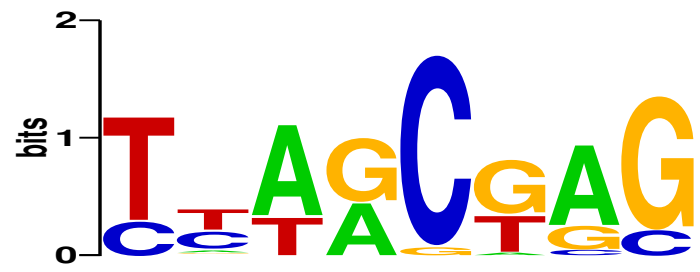	0.37959	No
155	YAP5_H2O2Hi	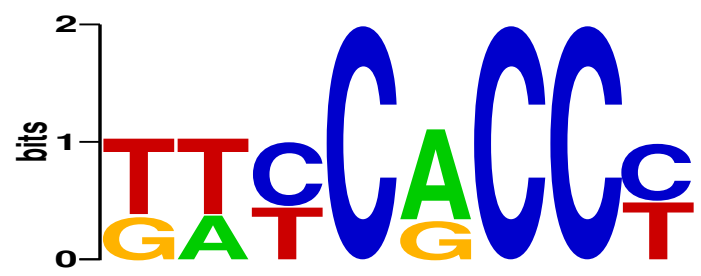	0.41041	No
156	MSN4_RAPA	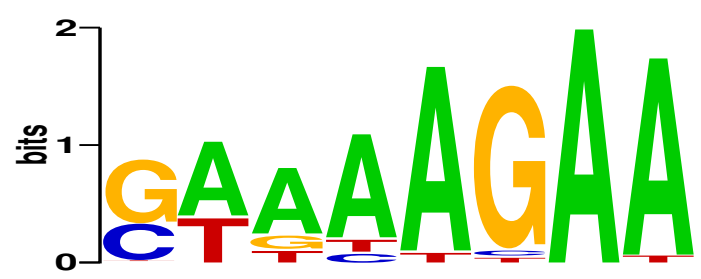	0.66843	No

Figure S2. Motifs learned by PRIORITY-$\mathcal{DN}$ on the 156 sequence-sets with known motifs. The motifs are ranked according to their p-values. The p-values are computed from the normal distribution of scores learned on random sequence-sets with the same cardinality.

*We disregard motifs that resemble the yeast repeat `TGTGTGTG` or `CACACACA` when we compute the precision-recall curve in Figure S3. Although they have a low p-value, they are not likely to be true motifs. See text for more details.

**As mentioned in the text, the motif learned from Ste12_BUT14 matches the Tec1 motif, while the motif learned from Tec1_Alpha matches the Ste12 motif.

#The logos of the motifs were generated using the web based program called WebLogo by Crooks *et al.* (2004).
