# Supplementary material for: A Nucleosome-Guided Map of Transcription Factor Binding Sites in Yeast
Source: PLoS Comput Biol. 2007 Nov 9;3(11):e215. doi: 10.1371/journal.pcbi.0030215 (PMC2065891; doi:10.1371/journal.pcbi.0030215)
Supplement: Supplementary file 4 [file pcbi.0030215.sg003.pdf]

(**A**) Precision-recall curve on sequence-sets with known motifs

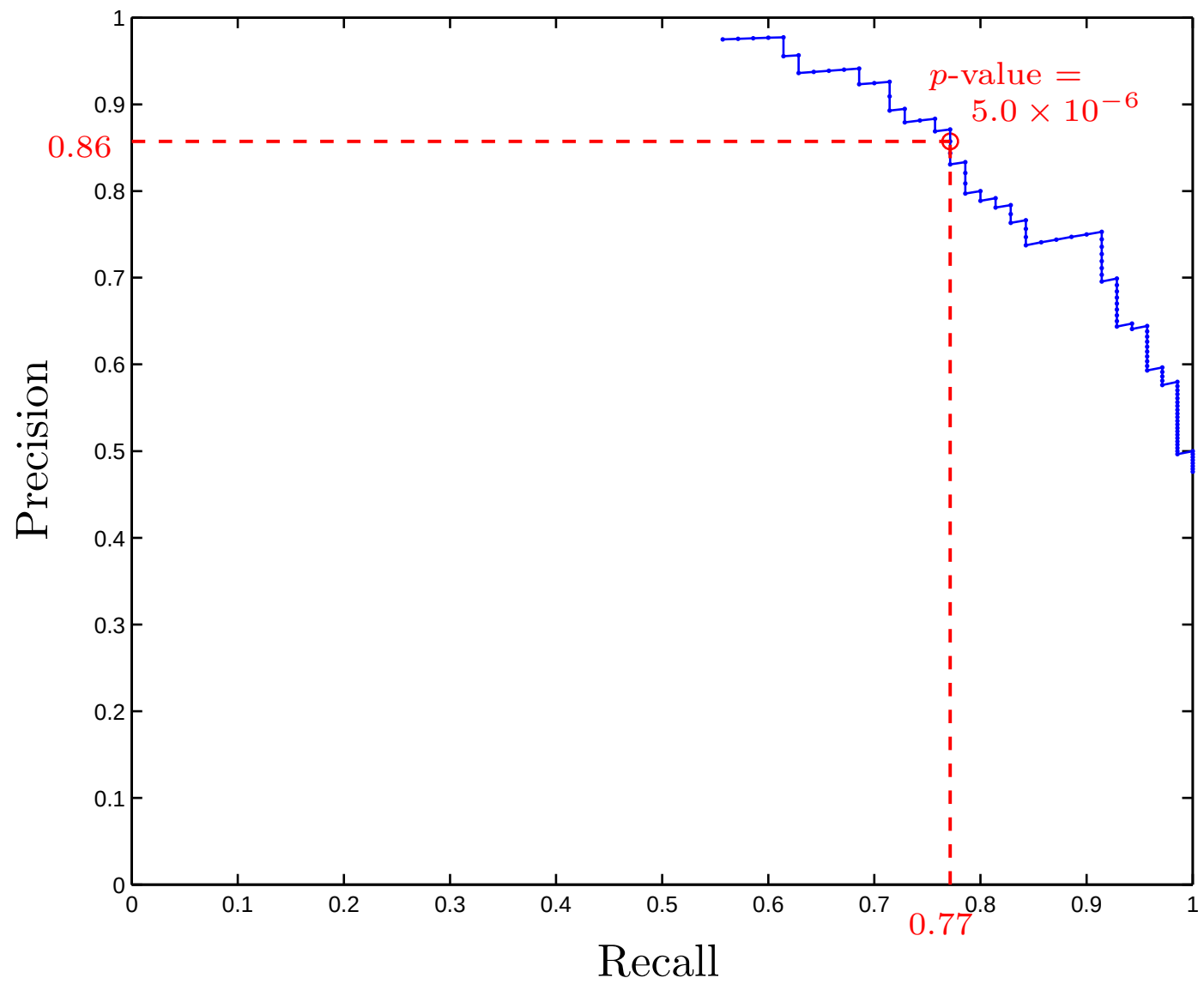

(**B**) Receiver operating characteristic curve on sequence-sets with known motifs

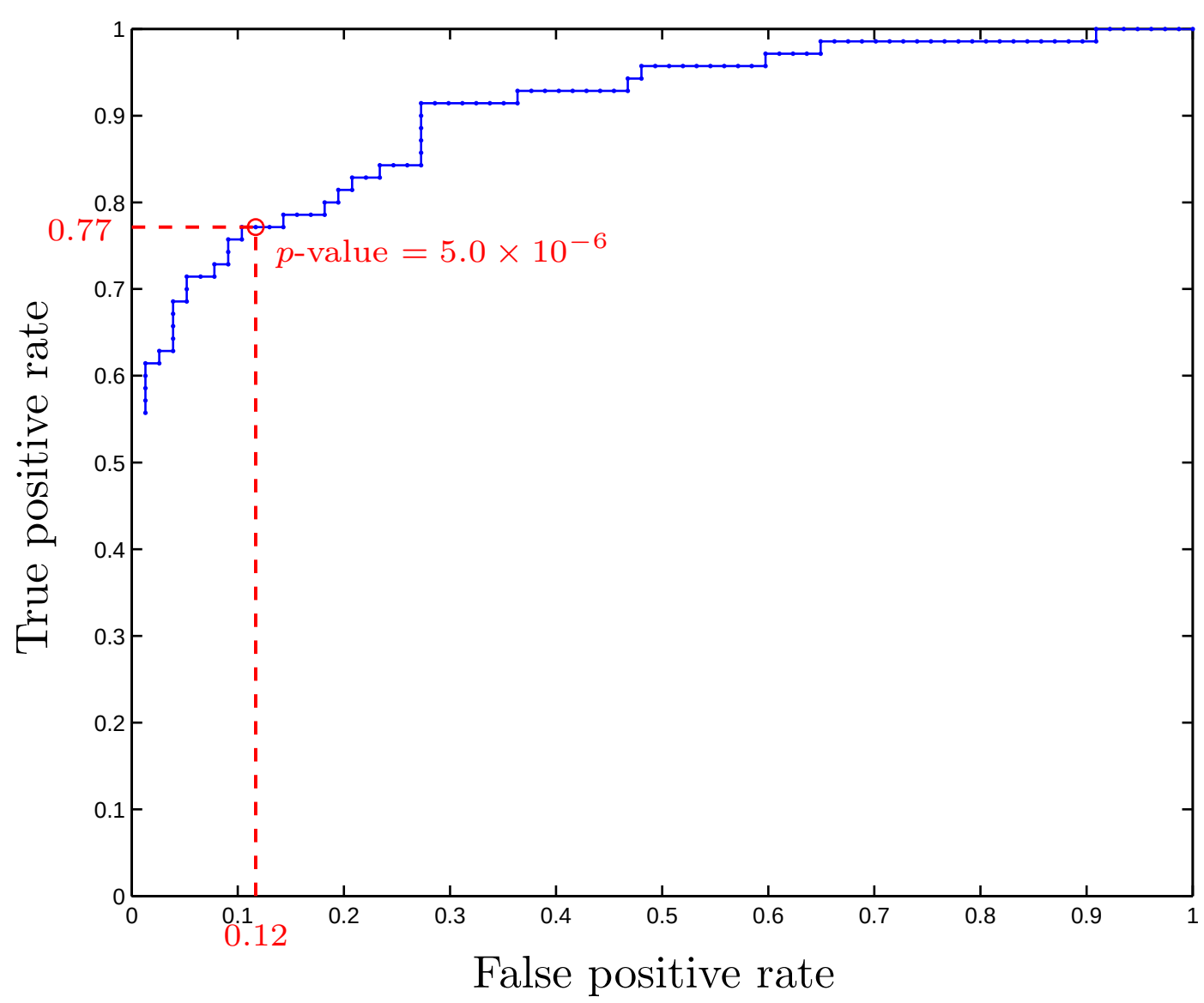

Figure S3. Use of p-values to detect significant motifs. We compute p-values for each motif learned from the 156 sequence-sets with known motifs (see Figure S2). After removing nine motifs resembling the poly(`GT`) tracts, we are left with 70 that match the literature (which we call true positives) and 77 that do not match the literature (which we call false positives). To find out how well the p-value differentiates between the true and the false positives, we plot the (**A**) precision-recall curve and (**B**) receiver operating characteristic curve. We can thus find a p-value cut-off which yields a low false discovery rate and use it to predict novel motifs with high confidence. As an example, both figures show an operating point of p-value 5.0×10^{-6}, where the false discovery rate is less than 15%. This is the operating point mentioned in the text.
