# Supplementary material for: A Nucleosome-Guided Map of Transcription Factor Binding Sites in Yeast
Source: PLoS Comput Biol. 2007 Nov 9;3(11):e215. doi: 10.1371/journal.pcbi.0030215 (PMC2065891; doi:10.1371/journal.pcbi.0030215)
Supplement: Supplementary file 5 [file pcbi.0030215.sg004.pdf]

No.	Sequence-set	Motif#	p-value
1	FHL1_RAPA	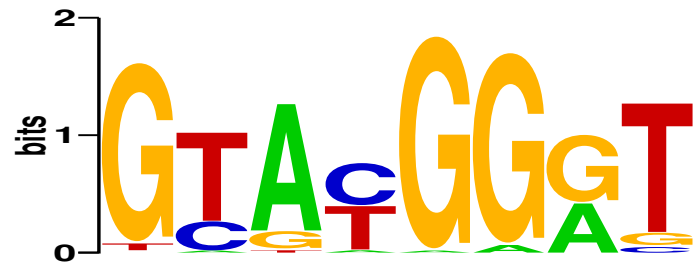	0
2	FHL1_SM	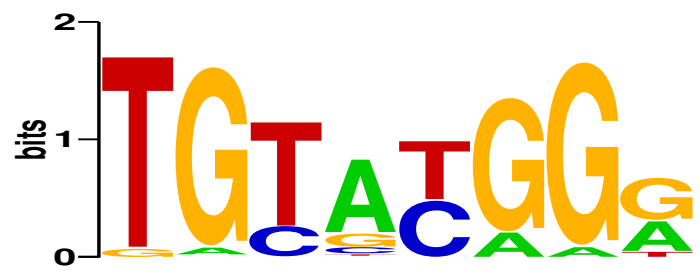	0
3	FHL1_YPD	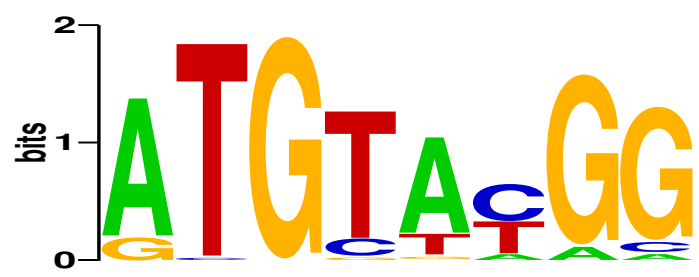	0
4	IME4_YPD	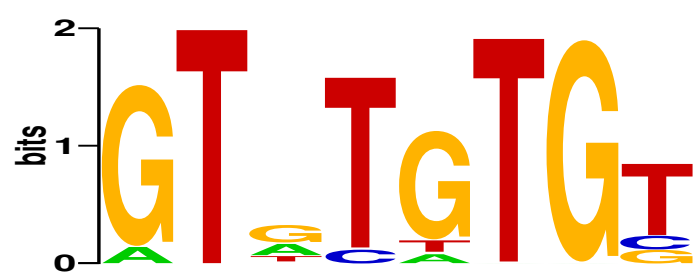	0*
5	MAL33_H2O2Hi	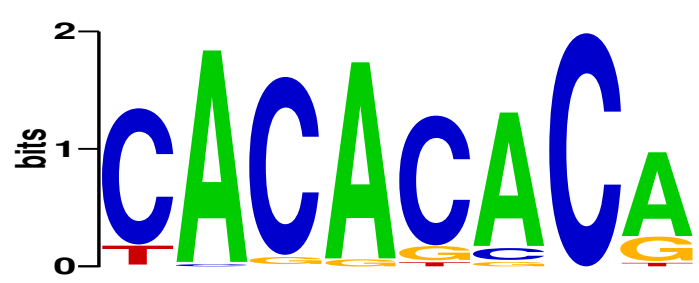	0*
6	SFP1_SM	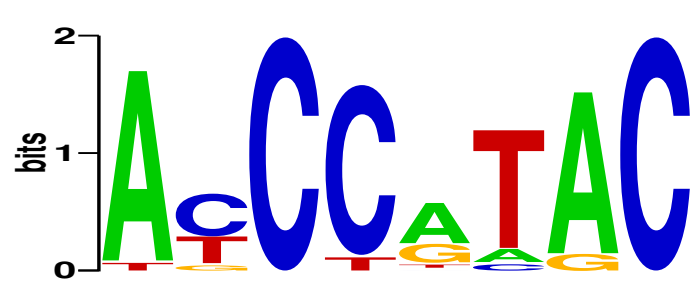	0
7	SNT2_YPD	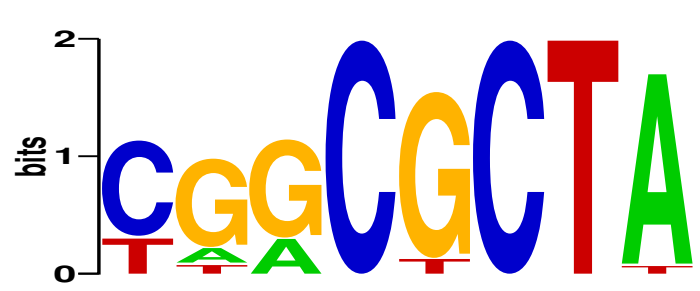	0
8	YDR026c_YPD	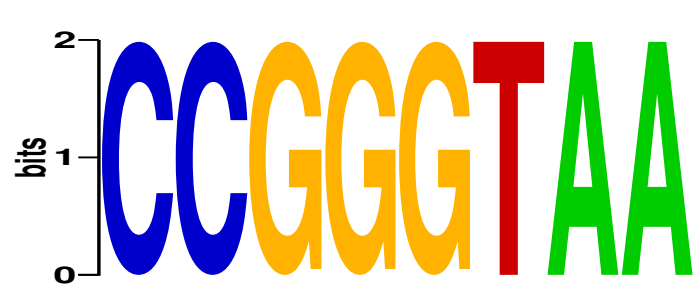	0
9	STB1_YPD	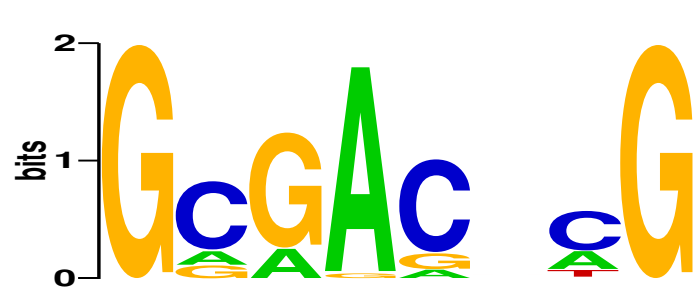	2.0475e-11
10	GAT3_YPD	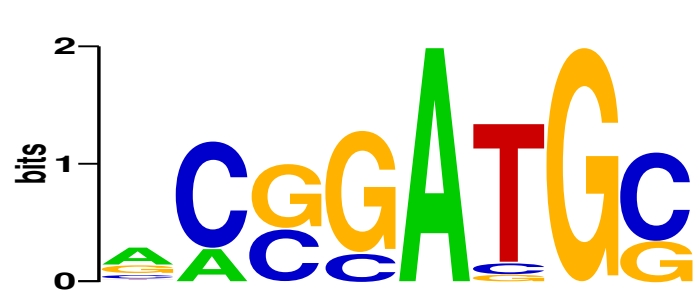	9.6814e-10

No.	Sequence-set	Motif	*p*-value
11	DIG1_Alpha	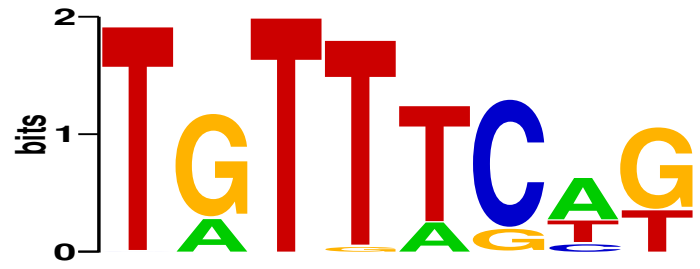	7.9782e-09
12	YJL206C_YPD	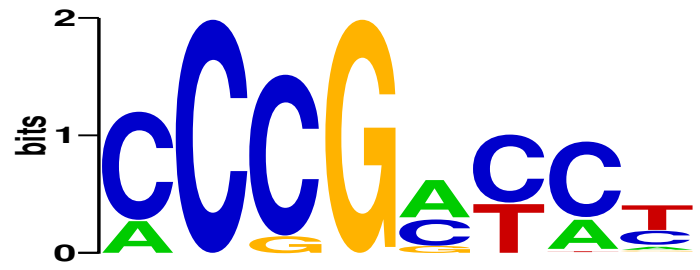	9.9985e-09
13	DIG1_BUT90	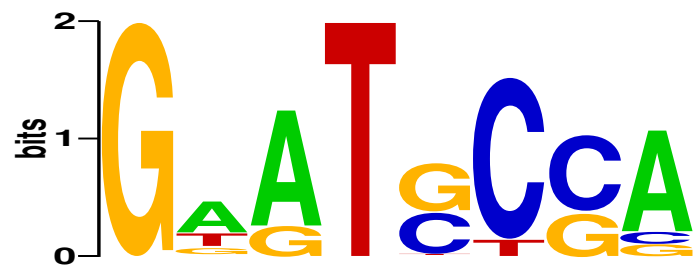	2.6087e-07
14	SOK2_BUT14	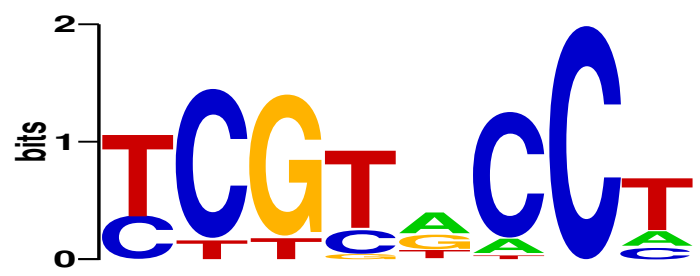	2.8795e-07
15	PHD1_BUT90	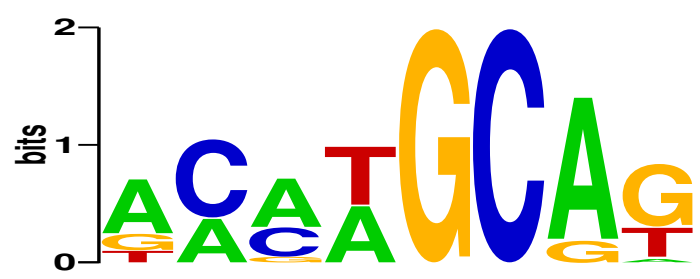	2.5892e-06
16	RFX1_YPD	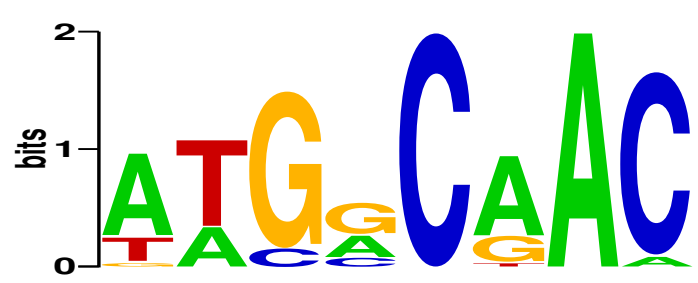	2.8015e-06
17	CST6_YPD	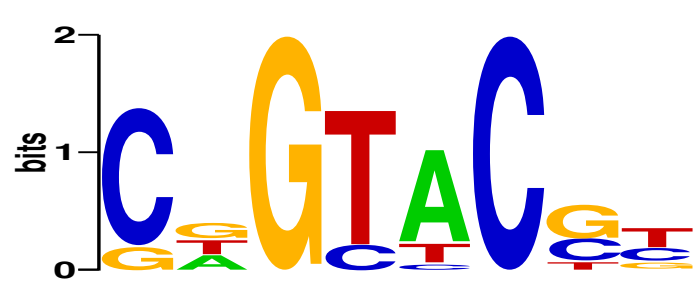	6.2154e-06
18	ARG81_SM	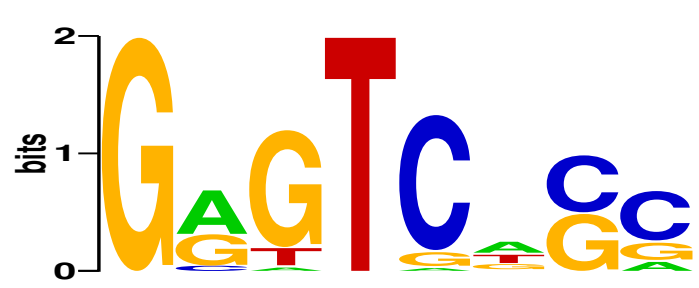	1.5214e-05
19	DIG1_BUT14	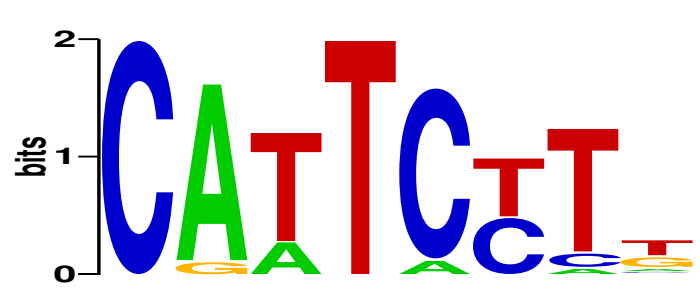	1.7175e-05
20	PHD1_YPD	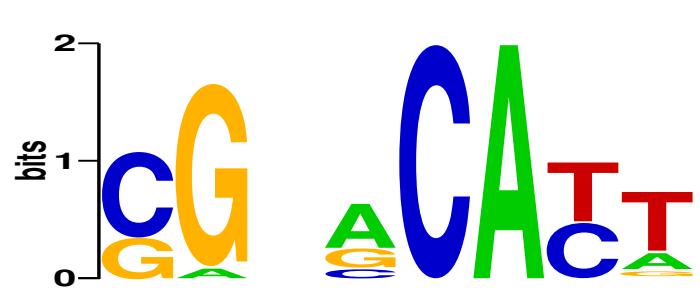	2.2941e-05

No.	Sequence-set	Motif	*p*-value
21	DAT1_YPD	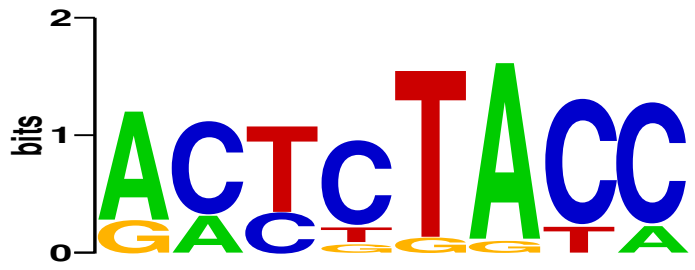	2.7442e-05
22	RDS1_H2O2Hi	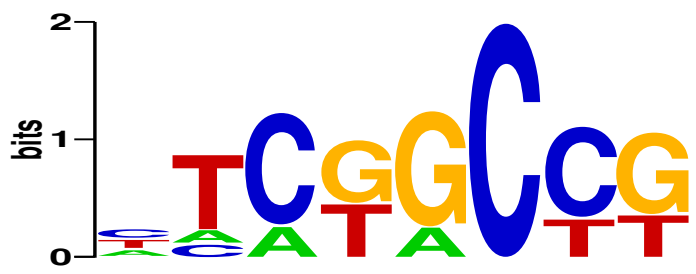	2.8499e-05
23	RME1_YPD	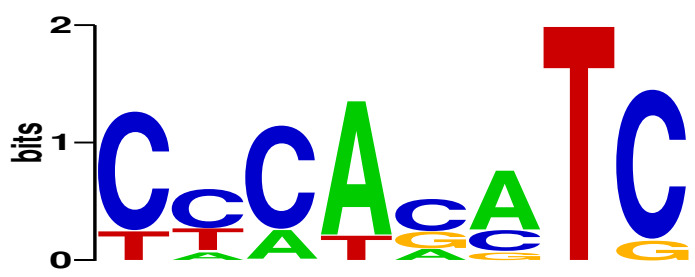	3.3738e-05
24	GCR2_YPD	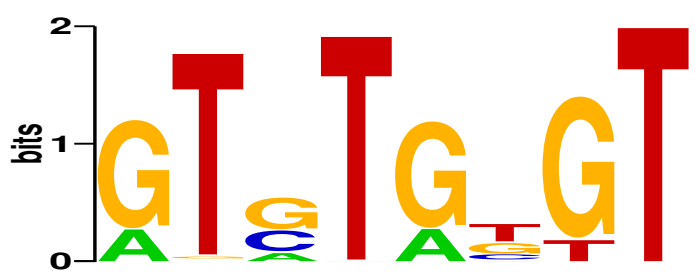	5.3452e-05*
25	YJL206C_H2O2Hi	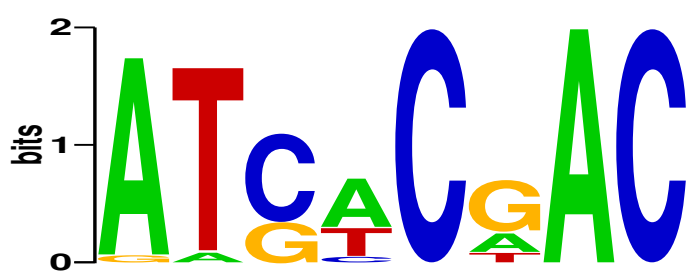	5.4521e-05
26	PHO2_H2O2Hi	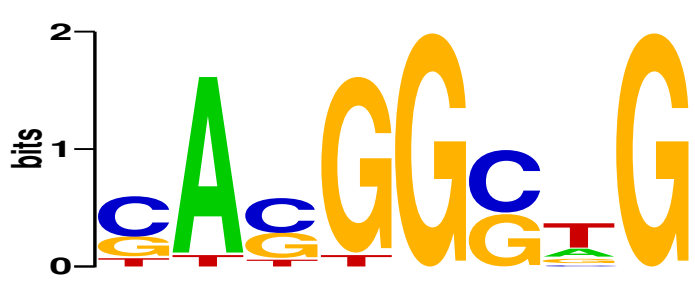	6.4884e-05
27	CHA4_SM	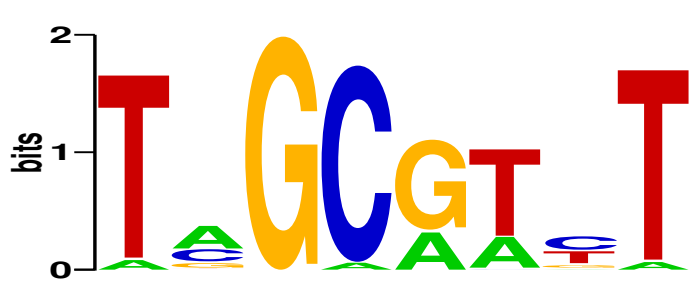	0.00017939
28	MET4_SM	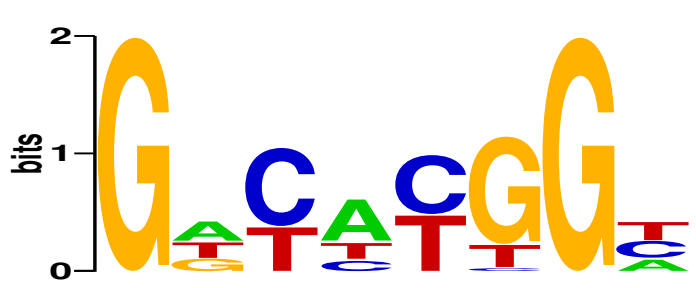	0.00019246
29	GTS1_YPD	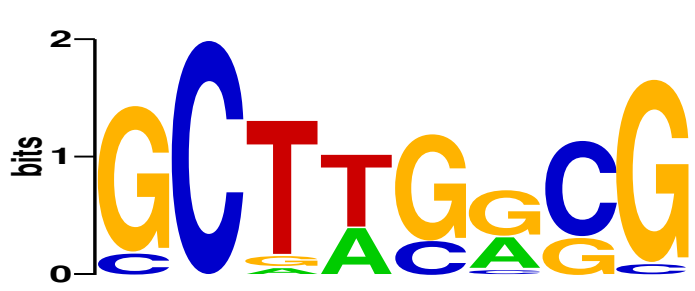	0.00020434
30	NDD1_YPD	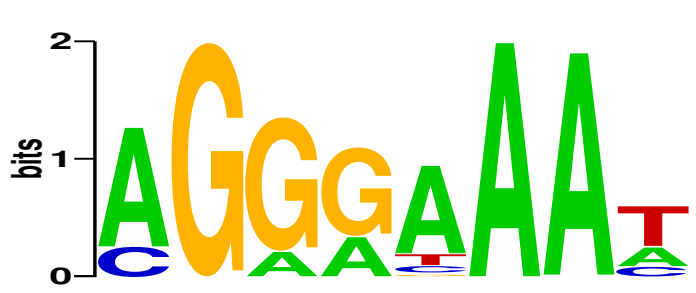	0.00034416

No.	Sequence-set	Motif	p-value
31	SFP1_H2O2Lo	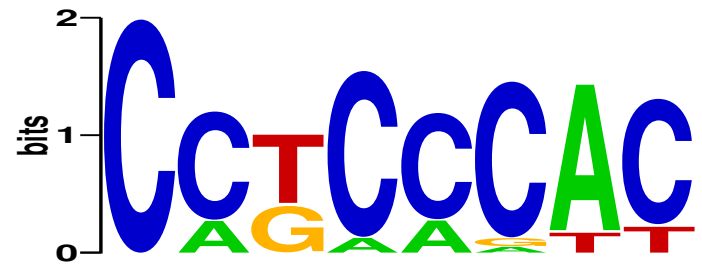	0.00045344
32	KSS1_YPD	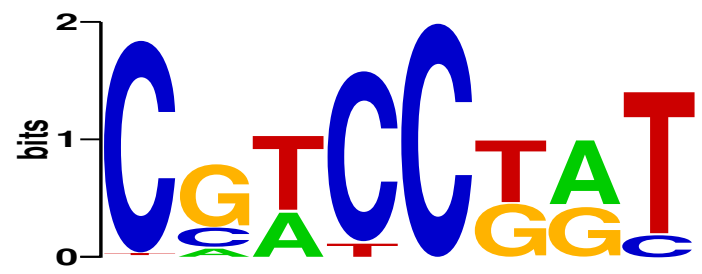	0.00050743
33	ARO80_SM	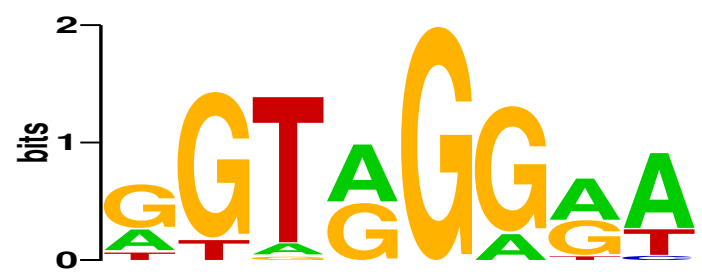	0.00086676
34	SRD1_YPD	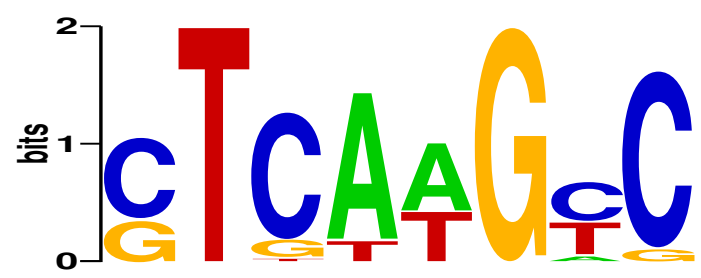	0.0013084
35	PDC2_YPD	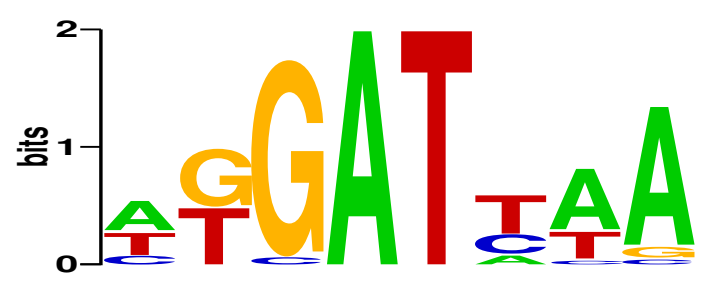	0.0013111
36	STB4_YPD	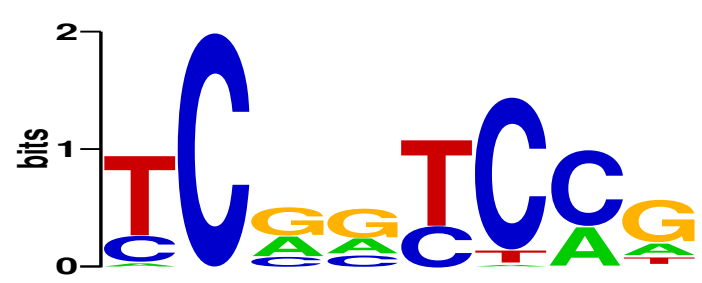	0.0022358
37	STP4_YPD	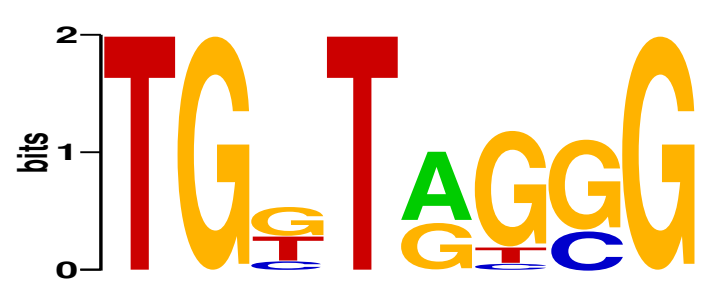	0.0024053
38	PIP2_YPD	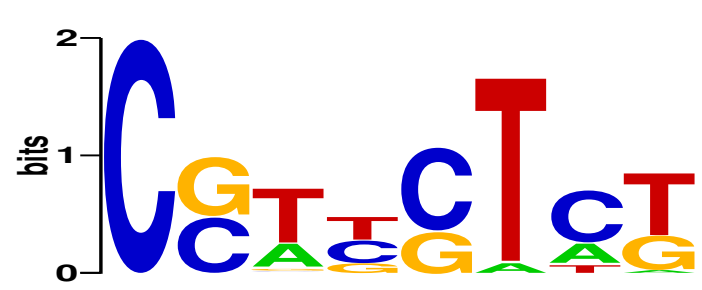	0.0025423
39	UME1_H2O2Hi	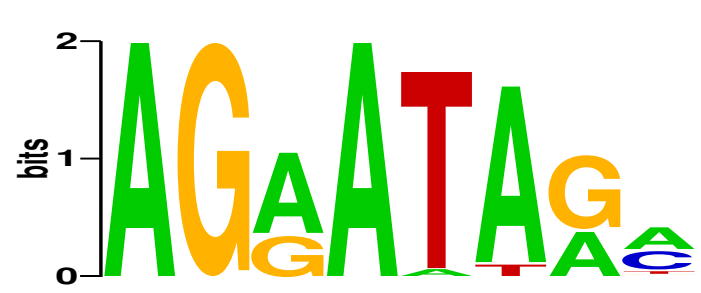	0.0028082
40	ARO80_YPD	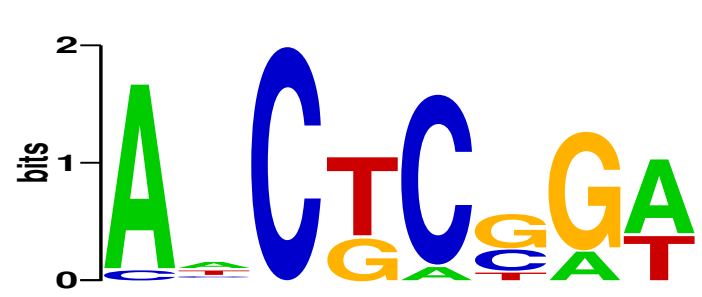	0.0034618

No.	Sequence-set	Motif	*p*-value
41	PHO2_SM	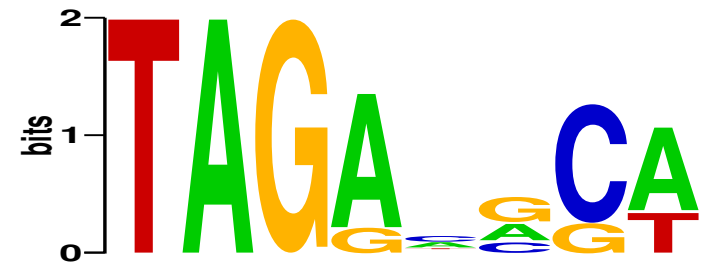	0.0036568
42	MIG2_YPD	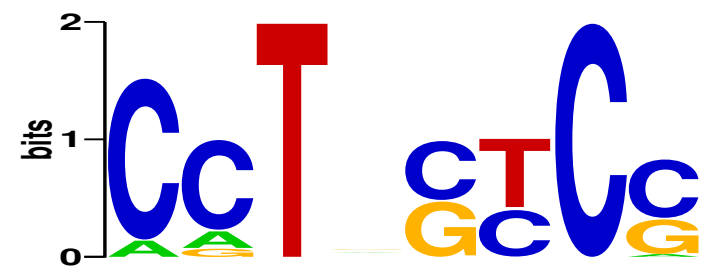	0.0039971
43	CUP9_YPD	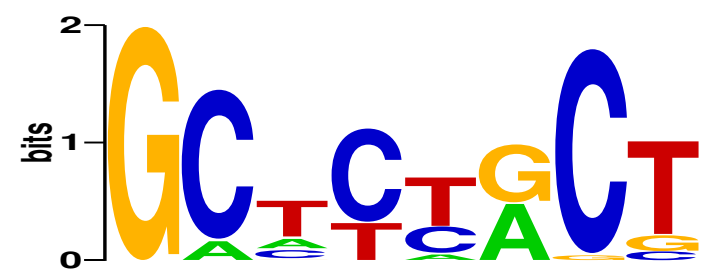	0.0040281
44	ASK10_YPD	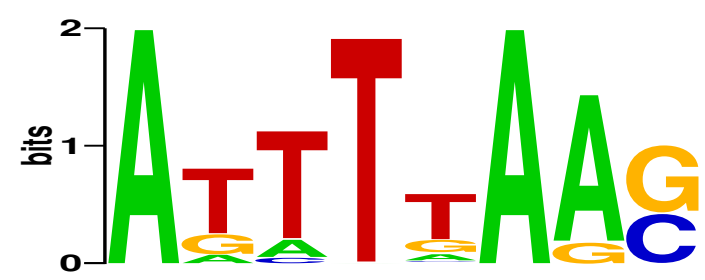	0.0046682
45	MIG1_YPD	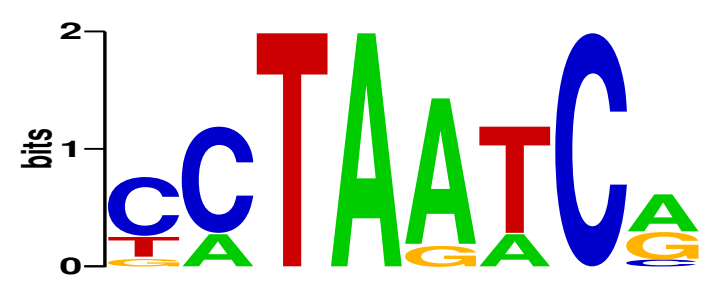	0.0048861
46	FZF1_YPD	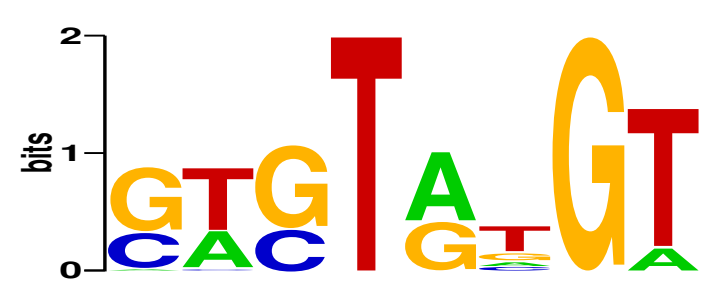	0.0052229*
47	OAF1_YPD	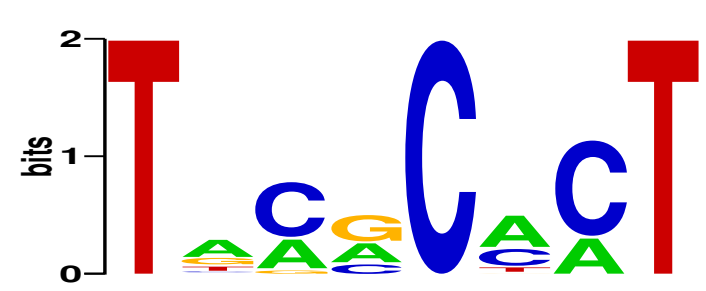	0.0053257
48	IME4_H2O2Hi	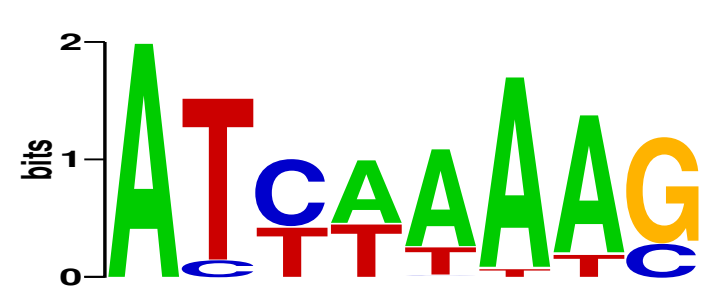	0.0070898
49	YER130C_YPD	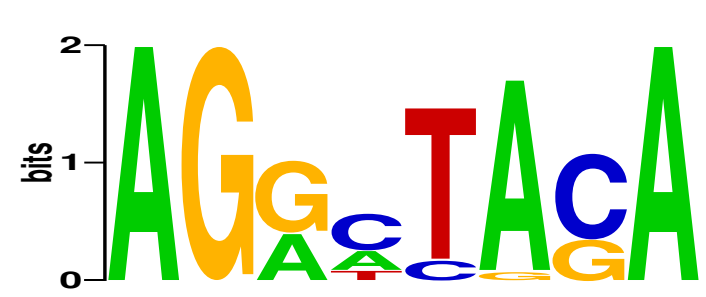	0.0074195
50	MET18_YPD	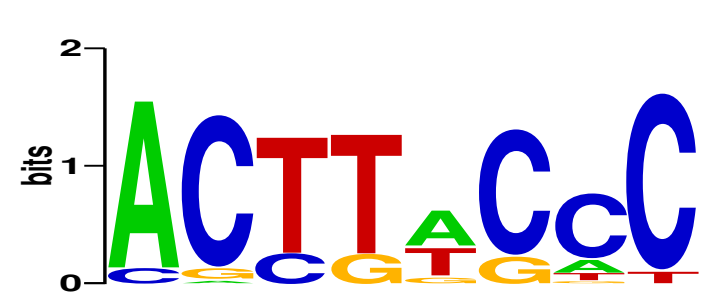	0.0097728

No.	Sequence-set	Motif	p-value
51	ARG80_YPD	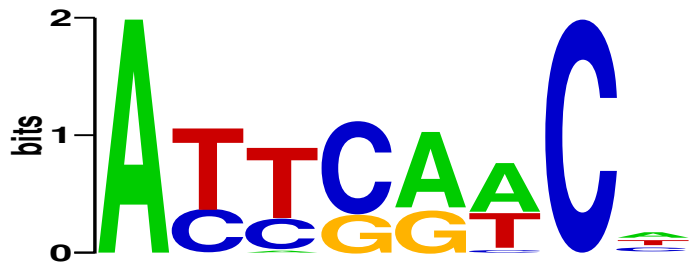	0.010513
52	IXR1_YPD	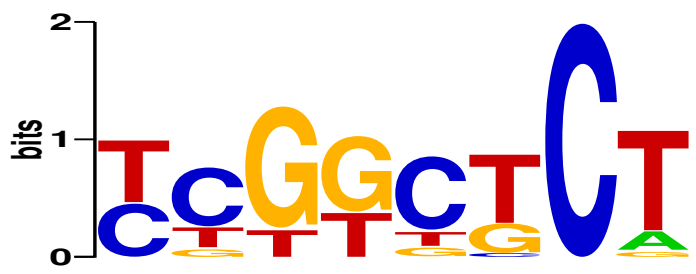	0.011083
53	UME1_YPD	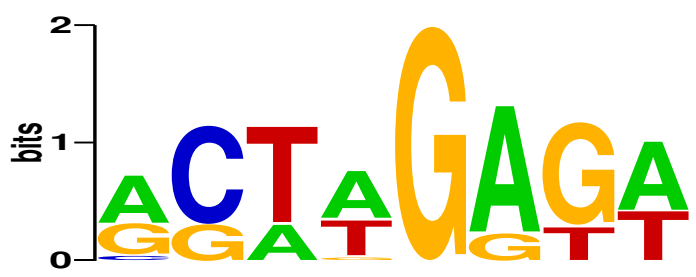	0.011408
54	HAL9_YPD	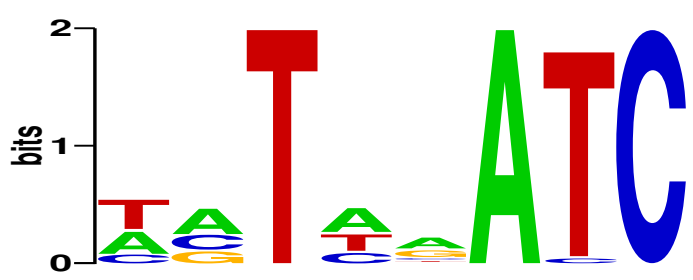	0.014647
55	UPC2_YPD	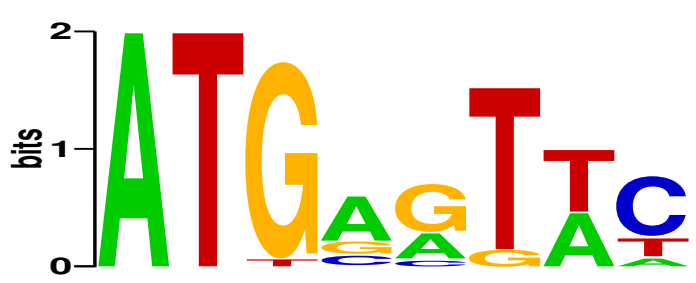	0.015817
56	HIR3_YPD	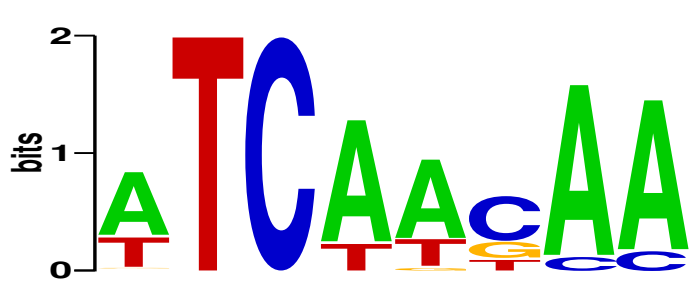	0.018132
57	PHO2_YPD	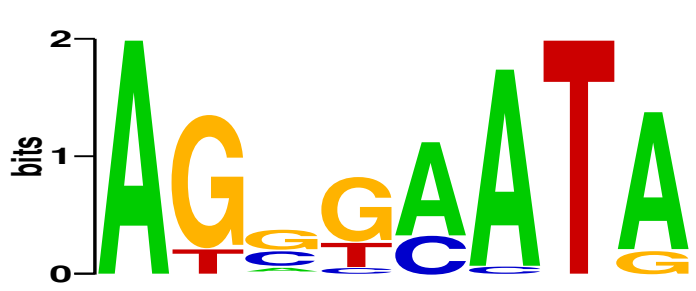	0.01841
58	SPT2_YPD	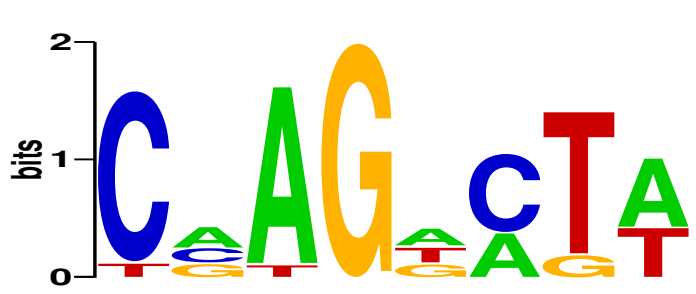	0.019389
59	MSS11_BUT14	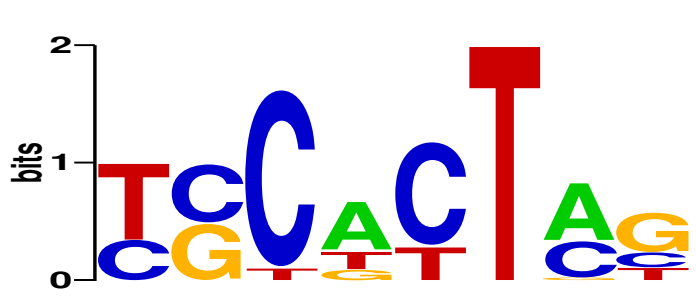	0.020365
60	DIG1_YPD	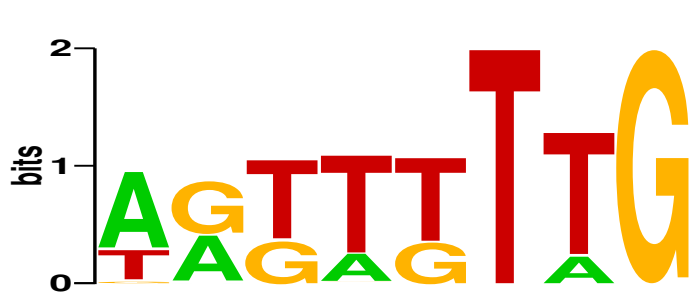	0.023323

No.	Sequence-set	Motif	p-value
61	MGA1_YPD	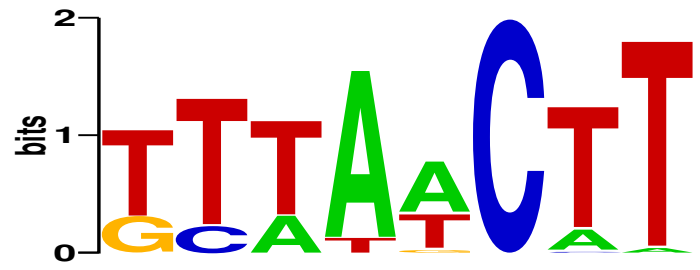	0.025555
62	THI2_Thi-	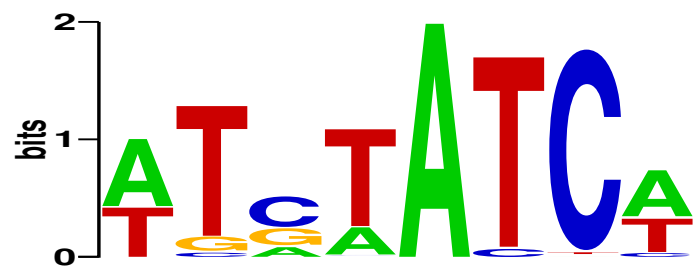	0.037419
63	HIR1_YPD	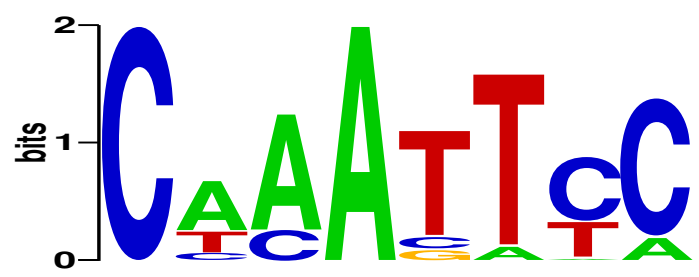	0.038401
64	YML081W_YPD	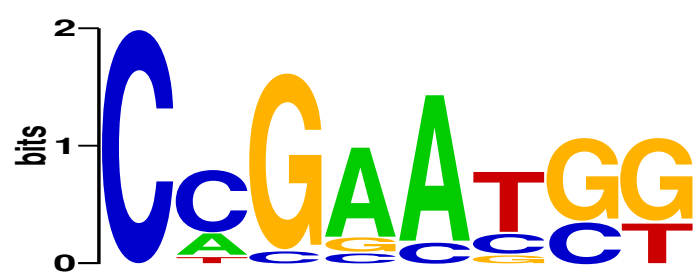	0.056814
65	NDT80_YPD	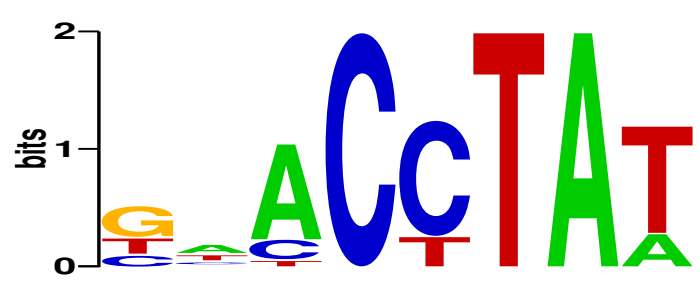	0.057773
66	WTM1_YPD	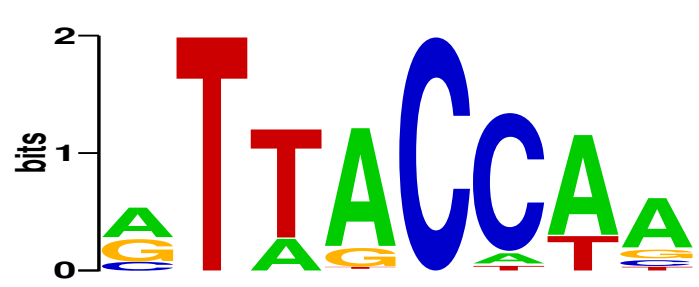	0.062581
67	ARG81_YPD	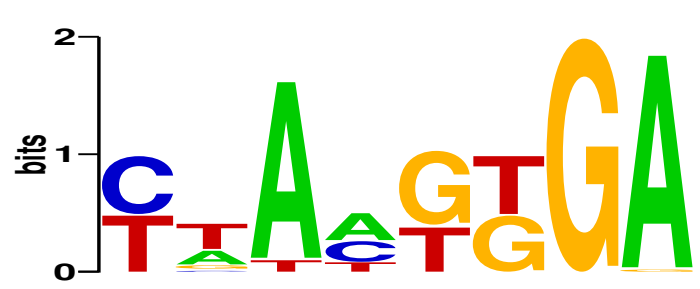	0.072655
68	YRR1_YPD	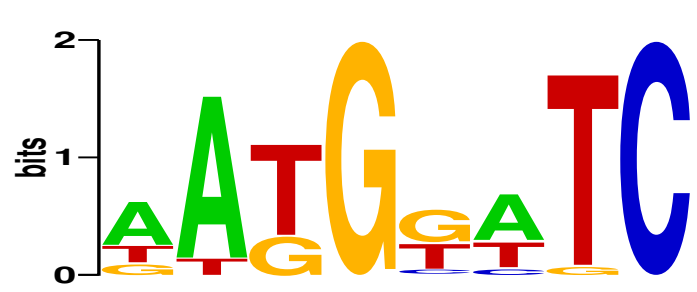	0.076884
69	WAR1_YPD	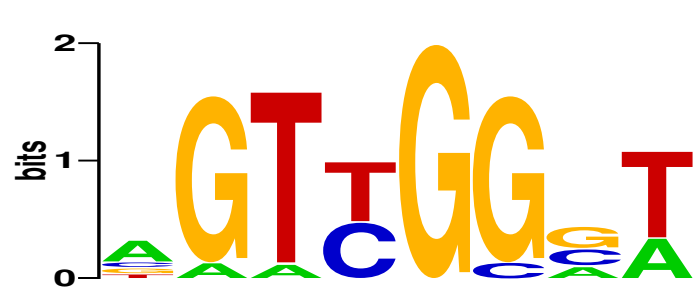	0.09921
70	GCR2_SM	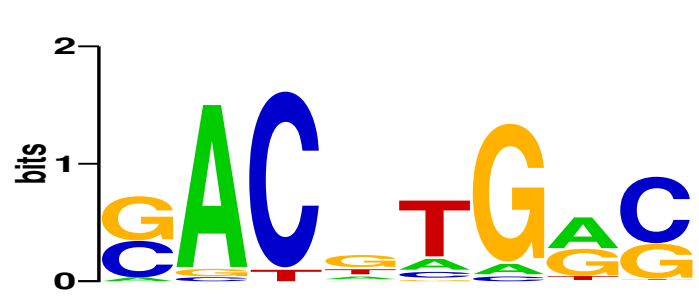	0.10588

No.	Sequence-set	Motif	p-value
71	YFL044C_YPD	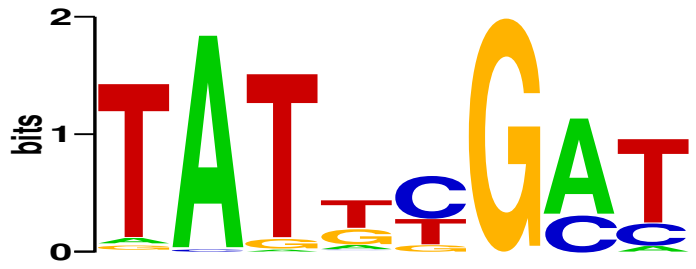	0.1105
72	SIG1_H2O2Hi	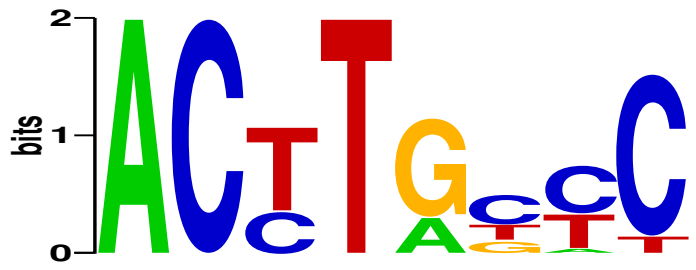	0.1285
73	MET28_YPD	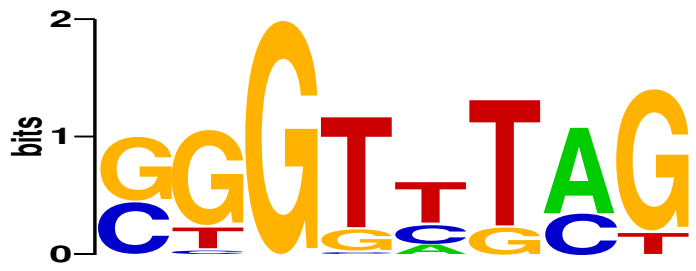	0.13216
74	HMS2_YPD	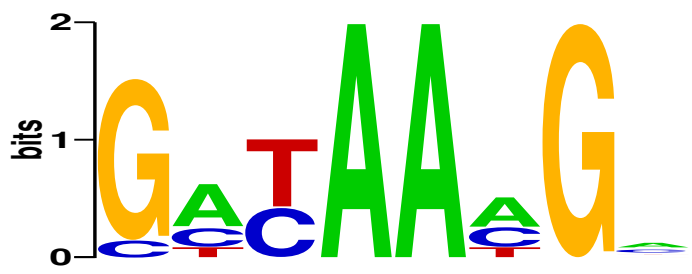	0.13899
75	STB2_YPD	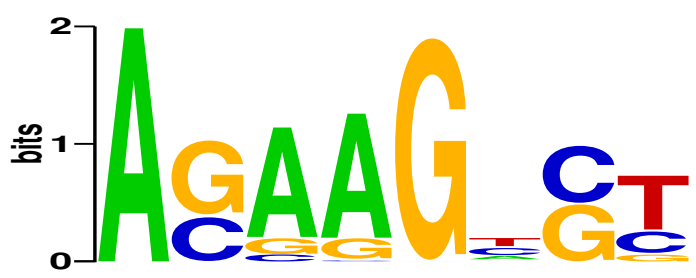	0.17031
76	RLR1_YPD	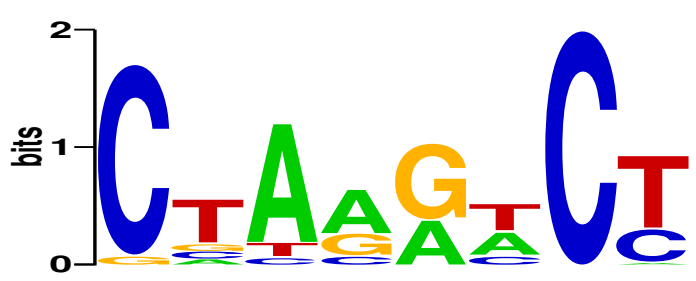	0.19307
77	STP2_YPD	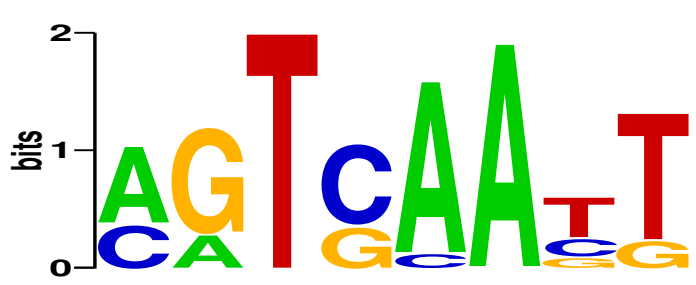	0.20643
78	PPR1_YPD	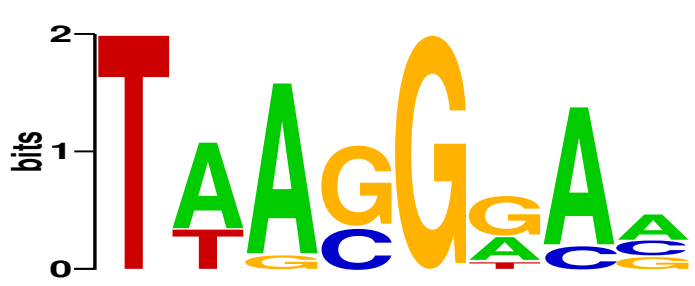	0.27217
79	SPT23_YPD	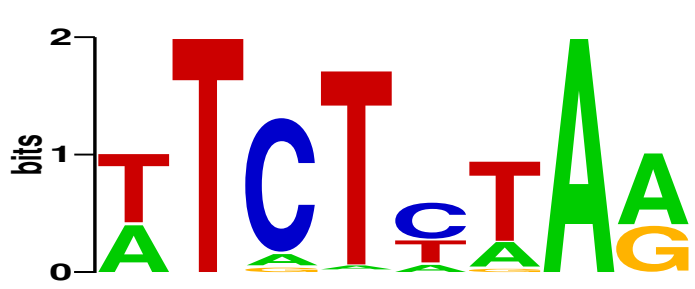	0.27626
80	HOG1_YPD	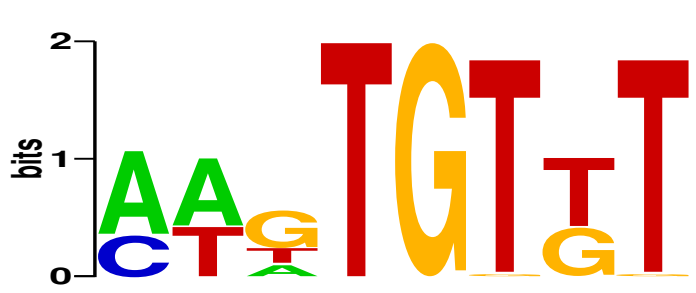	0.33553

No.	Sequence-set	Motif	p-value
81	HMS1_YPD	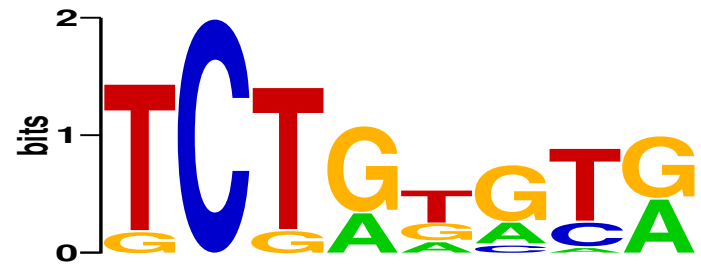	0.33582
82	HIR2_YPD	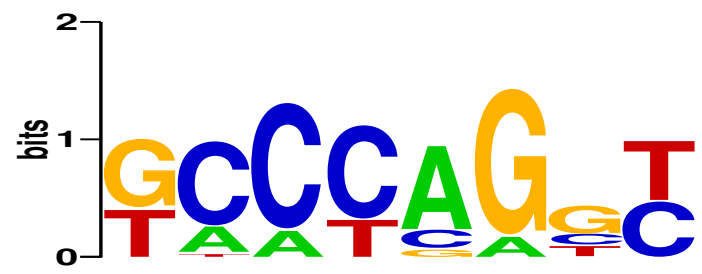	0.49247

Figure S4. Novel motifs learned by PRIORITY-$\mathcal{DN}$ on 82 sequence-sets. The motifs are ranked according to their p-values. The p-values are computed from the normal distribution of scores learned on random sequence-sets with the same cardinality.

[*]When we look for motifs with high significance, *i.e.* those having a p-value less than some cutoff, we disregard motifs that resemble the yeast repeat `TGTGTGTG` or `CACACACA`. See text for more details.

[#]The logos of the motifs were generated using the web based program called WebLogo by Crooks *et al.* (2004).
