# Supplementary material for: A Nucleosome-Guided Map of Transcription Factor Binding Sites in Yeast
Source: PLoS Comput Biol. 2007 Nov 9;3(11):e215. doi: 10.1371/journal.pcbi.0030215 (PMC2065891; doi:10.1371/journal.pcbi.0030215)
Supplement: Supplementary file 6 [file pcbi.0030215.st001.pdf]

Table S1. Comparison of priority using various positional priors with state-of-the-art motif discovery programs. A '1' in a cell represents represents a match to the literature consensus of the top-scoring motif learned by the respective program. A '0' represents no match. The programs being compared, along with their shorthand designations, are

- $\mathcal{U}$: PRIORITY-$\mathcal{U}$, *i.e.* Gibbs sampling with a uniform positional prior
- $\mathcal{N}$: PRIORITY-$\mathcal{N}$, *i.e.* Gibbs sampling with prior $\mathcal{N}$, calculated based on the $\mathcal{S}_{\mathcal{N}}$ score from the computational predictions of Segal *et al.*
- $\mathcal{DN}$: PRIORITY-$\mathcal{DN}$, *i.e.* Gibbs sampling with prior $\mathcal{DN}$, calculated based on the $\mathcal{S}_{\mathcal{DN}}$ score from the computational predictions of Segal *et al.*
- $\mathcal{DN}'$: PRIORITY-$\mathcal{DN}'$, *i.e.* Gibbs sampling with prior $\mathcal{DN}'$, calculated based on the $\mathcal{S}_{\mathcal{DN}}$ score from the *in vivo* data of Lee *et al.*
- $\mathcal{D}$: PRIORITY-$\mathcal{D}$, *i.e.* Gibbs sampling with a simplification of prior $\mathcal{DN}$, calculated in the absence of nucleosome occupancy data as described in the Discussion
- A : AlignACE
- M : MEME
- MD : MDscan
- M_c : MEME_c, *i.e.* MEME using conservation information
- C : CONVERGE, a conservation based method of Harbison *et al.*
- K : the conservation based method of Kellis *et al.*

No.	Sequence-set	$\mathcal{U}$	$\mathcal{N}$	$\mathcal{DN}$	$\mathcal{DN}'$	$\mathcal{D}$	A	M	MD	M_c	C	K
1	ABF1_YPD	1	1	1	1	1	1	1	0	1	1	1
2	ACE2_YPD	0	0	0	0	0	0	0	0	0	0	1
3	ADR1_HEAT	0	0	0	0	0	0	0	0	0	0	0
4	ADR1_YPD	0	0	0	0	0	0	0	0	0	0	0
5	AFT2_H2O2Hi	0	1	1	0	0	0	0	0	0	0	0
6	AFT2_H2O2Lo	1	1	1	1	1	0	1	1	1	1	0
7	AFT2_YPD	0	0	0	0	0	0	0	0	0	0	0
8	ARR1_YPD	0	0	0	0	0	0	0	0	0	0	0
9	ASH1_BUT14	0	0	0	0	0	0	0	0	0	0	0
10	AZF1_YPD	0	0	0	0	0	1	1	0	1	1	0
11	BAS1_SM	1	1	1	1	1	0	1	0	1	1	1
12	BAS1_YPD	1	1	1	1	1	0	0	1	1	1	1
13	CAD1_SM	1	1	1	0	1	0	0	1	1	0	0
14	CAD1_YPD	1	1	1	1	1	0	1	0	1	1	0
15	CBF1_SM	1	1	1	1	1	0	0	1	0	1	1

No.	Sequence-set	$\mathcal{U}$	$\mathcal{N}$	$\mathcal{DN}$	$\mathcal{DN}'$	$\mathcal{D}$	A	M	MD	M_c	C	K
16	CBF1_YPD	1	1	1	1	1	1	1	1	1	1	0
17	CIN5_H2O2Hi	0	0	1	1	0	0	0	0	0	1	0
18	CIN5_H2O2Lo	1	0	1	1	1	0	0	0	0	1	1
19	CIN5_YPD	0	0	1	1	1	0	0	1	0	0	0
20	DAL80_RAPA	0	0	0	0	0	0	0	0	0	0	0
21	DAL80_YPD	0	0	0	0	0	0	0	0	0	0	0
22	DAL81_RAPA	0	0	0	0	0	0	0	0	0	0	0
23	DAL81_YPD	0	0	0	0	0	0	0	0	0	0	0
24	DAL82_RAPA	0	0	1	0	0	0	0	0	0	1	0
25	DAL82_SM	0	1	1	0	0	0	0	0	1	1	0
26	DAL82_YPD	0	0	0	0	0	0	0	1	0	0	1
27	FKH1_YPD	1	1	1	1	1	1	1	1	1	1	1
28	FKH2_H2O2Hi	1	1	1	1	1	1	0	1	1	1	1
29	FKH2_H2O2Lo	1	1	1	1	1	1	1	0	1	1	0
30	FKH2_YPD	1	1	1	1	1	1	0	1	1	1	1
31	GAL4_GAL	0	0	0	0	0	0	0	0	0	0	1
32	GAL4_RAFF	0	0	0	0	0	0	0	0	0	0	1
33	GAL4_YPD	0	0	0	0	0	0	0	0	1	0	1
34	GAT1_RAPA	0	0	1	1	0	0	0	0	1	0	1
35	GAT1_SM	0	0	0	1	0	0	0	0	0	1	0
36	GCN4_RAPA	1	1	1	1	1	0	1	1	1	1	1
37	GCN4_SM	1	1	1	1	1	0	0	1	1	1	1
38	GCN4_YPD	1	1	1	1	1	0	1	1	1	1	1
39	GCR1_YPD	0	0	1	0	0	0	1	1	0	0	0
40	GLN3_RAPA	1	1	1	1	1	0	1	1	1	1	1
41	GLN3_SM	0	0	0	0	0	0	0	0	0	0	0
42	GLN3_YPD	0	0	0	0	0	1	0	1	0	0	0
43	GZF3_H2O2Hi	0	0	0	0	0	0	0	0	0	0	0
44	GZF3_RAPA	0	0	0	0	0	0	0	0	0	0	0
45	HAC1_YPD	1	1	1	1	1	0	1	1	1	0	0
46	HAP1_YPD	0	0	1	1	1	0	0	0	0	1	1
47	HAP2_RAPA	0	0	1	1	1	0	0	0	0	0	0
48	HAP2_YPD	0	0	0	0	0	0	0	0	0	0	1
49	HAP3_YPD	0	1	1	1	0	0	0	0	0	0	0

No.	Sequence-set	$\mathcal{U}$	$\mathcal{N}$	$\mathcal{DN}$	$\mathcal{DN}'$	$\mathcal{D}$	A	M	MD	M_c	C	K
50	HAP4_H2O2Lo	0	0	0	0	0	0	0	0	0	0	0
51	HAP4_YPD	1	1	1	1	1	0	1	0	1	1	0
52	HAP5_SM	0	0	0	0	0	0	0	0	0	0	0
53	HAP5_YPD	0	0	0	0	0	0	0	0	0	0	0
54	HSF1_H2O2Hi	0	0	1	1	1	0	0	1	1	1	1
55	HSF1_H2O2Lo	1	0	1	1	1	0	1	1	1	1	1
56	INO2_YPD	1	1	1	1	1	0	1	1	1	1	1
57	INO4_YPD	1	1	1	1	1	0	1	1	1	1	1
58	LEU3_SM	1	1	1	1	1	0	1	1	1	1	1
59	LEU3_YPD	0	1	1	1	1	0	0	1	0	0	1
60	MAC1_H2O2Hi	0	0	1	0	0	0	0	1	0	0	0
61	MAC1_YPD	0	0	1	1	0	0	0	0	0	0	0
62	MBP1_H2O2Hi	1	1	1	1	1	0	1	1	1	1	1
63	MBP1_H2O2Lo	1	1	1	1	1	0	1	1	1	1	1
64	MBP1_YPD	0	1	1	1	1	0	0	1	0	1	1
65	MCM1_Alpha	0	1	1	1	1	1	1	1	1	1	0
66	MCM1_YPD	0	1	1	1	1	0	1	1	1	1	0
67	MET31_SM	0	0	0	0	0	0	0	0	0	0	0
68	MET31_YPD	0	0	0	0	0	0	0	0	0	0	0
69	MET32_SM	0	0	0	0	0	0	0	0	0	0	0
70	MET32_YPD	0	0	0	0	0	0	0	0	0	0	0
71	MOT3_SM	0	0	0	0	0	0	0	0	0	0	0
72	MOT3_YPD	0	0	0	0	0	0	0	0	0	0	0
73	MSN2_Acid	0	0	0	0	0	0	0	0	0	0	0
74	MSN2_H2O2Hi	0	0	0	0	1	0	0	1	0	0	0
75	MSN2_H2O2Lo	0	0	0	0	0	0	0	0	0	0	0
76	MSN2_RAPA	0	0	0	0	0	0	0	0	0	0	0
77	MSN4_Acid	0	1	1	0	0	0	0	0	0	0	0
78	MSN4_H2O2Hi	0	0	0	0	0	0	0	0	0	0	0
79	MSN4_H2O2Lo	0	0	0	0	0	0	0	0	0	0	0
80	MSN4_RAPA	0	0	0	0	0	0	0	0	0	0	0
81	MSN4_YPD	0	0	0	0	0	0	0	0	0	0	0
82	NRG1_H2O2Hi	1	1	1	1	1	0	0	1	1	1	0
83	NRG1_H2O2Lo	0	0	0	0	0	0	0	0	0	0	0

No.	Sequence-set	$\mathcal{U}$	$\mathcal{N}$	$\mathcal{DN}$	$\mathcal{DN}'$	$\mathcal{D}$	A	M	MD	M_c	C	K
84	NRG1_YPD	0	0	0	0	0	0	0	0	0	0	0
85	PDR1_H2O2Lo	0	0	0	0	0	0	0	0	0	0	0
86	PDR1_YPD	0	0	0	0	0	0	0	0	0	0	0
87	PDR3_YPD	0	0	0	0	0	0	0	0	0	0	0
88	PHO4_Pi-	1	1	1	1	1	0	1	1	1	1	1
89	PHO4_YPD	0	0	0	0	0	0	0	0	0	0	0
90	PUT3_SM	0	0	0	0	0	0	0	0	0	0	0
91	PUT3_YPD	0	0	0	0	0	0	1	0	1	0	0
92	RAP1_YPD	1	1	1	1	1	1	1	1	1	1	1
93	RCS1_H2O2Hi	1	1	1	1	1	0	1	1	1	1	0
94	RCS1_H2O2Lo	1	1	1	1	1	0	0	0	0	1	1
95	RCS1_SM	0	0	0	0	0	0	0	0	0	0	0
96	RCS1_YPD	0	0	0	0	0	0	0	0	0	0	0
97	REB1_H2O2Hi	1	1	1	1	1	1	1	1	1	1	1
98	REB1_H2O2Lo	1	1	1	1	1	0	0	0	0	1	1
99	REB1_YPD	1	1	1	1	1	1	1	1	1	1	1
100	RIM101_H2O2Hi	0	0	0	0	0	0	0	0	0	0	0
101	RLM1_YPD	0	0	0	0	0	0	0	0	0	0	0
102	ROX1_H2O2Hi	0	0	0	0	0	0	0	0	0	0	0
103	ROX1_H2O2Lo	0	0	0	0	0	0	0	0	0	0	0
104	ROX1_YPD	0	0	0	0	0	0	0	0	0	0	0
105	RPH1_H2O2Hi	0	0	0	0	0	0	0	0	0	0	0
106	RPH1_SM	0	0	0	0	0	0	0	0	0	0	0
107	RPH1_YPD	0	0	0	0	0	0	0	0	0	0	0
108	RPN4_H2O2Lo	1	1	1	1	1	1	1	1	1	1	1
109	RPN4_YPD	0	0	0	0	0	0	0	0	0	0	0
110	RTG1_RAPA	0	0	0	0	0	0	0	0	0	0	0
111	RTG1_SM	0	0	0	0	0	0	0	0	0	0	0
112	RTG3_H2O2Hi	0	0	0	0	0	0	0	0	0	0	0
113	RTG3_RAPA	0	0	0	0	0	0	0	0	0	0	0
114	RTG3_SM	0	0	0	0	0	0	0	0	0	0	0
115	RTG3_YPD	0	0	0	0	0	0	0	0	0	0	0
116	SIP4_SM	0	0	0	0	0	0	0	0	0	0	0
117	SIP4_YPD	0	0	0	0	0	0	0	0	0	0	0

No.	Sequence-set	$\mathcal{U}$	$\mathcal{N}$	$\mathcal{DN}$	$\mathcal{DN}'$	$\mathcal{D}$	A	M	MD	M_c	C	K
118	SKN7_H2O2Hi	0	0	1	1	0	0	0	1	0	0	0
119	SKN7_H2O2Lo	0	0	1	1	1	0	0	1	0	0	0
120	SKN7_YPD	1	0	1	1	1	0	0	1	1	0	0
121	SKO1_YPD	0	0	1	1	0	0	0	0	0	1	0
122	SMP1_YPD	0	0	0	0	0	0	0	0	0	0	0
123	STB5_YPD	0	1	1	1	1	0	0	1	1	1	1
124	STE12_Alpha	1	0	1	1	1	0	1	1	0	0	1
125	STE12_BUT14	0	0	0*	0	0	0	0	1	0	0	1
126	STE12_BUT90	0	0	1	1	1	0	0	1	0	1	1
127	STE12_YPD	1	0	1	1	1	0	0	1	1	0	1
128	STP1_SM	0	0	0	0	0	0	0	1	0	0	0
129	STP1_YPD	0	0	0	0	0	0	0	0	0	0	0
130	SUM1_YPD	1	1	1	1	1	1	0	1	1	1	0
131	SUT1_YPD	0	0	0	0	0	0	0	0	0	0	0
132	SWI4_YPD	0	1	1	1	1	0	0	0	0	1	1
133	SWI5_YPD	0	0	0	0	0	0	0	0	0	0	1
134	SWI6_YPD	1	1	1	1	1	0	0	0	1	0	0
135	TEC1_Alpha	0	0	0**	0	0	0	0	0	0	0	0
136	TEC1_BUT14	0	0	0	1	0	0	0	1	0	0	0
137	TEC1_YPD	0	0	0	1	1	0	0	0	0	1	1
138	TYE7_YPD	1	1	1	1	1	0	1	1	0	1	1
139	UGA3_RAPA	0	0	0	0	0	0	0	0	0	0	1
140	UGA3_SM	0	0	0	0	0	0	0	0	0	0	0
141	UME6_H2O2Hi	1	1	1	1	1	1	1	1	1	1	1
142	UME6_YPD	1	1	1	1	1	1	1	1	1	1	1
143	XBP1_H2O2Lo	0	0	0	0	0	0	0	0	0	0	0
144	YAP1_H2O2Lo	1	1	1	1	1	0	0	1	1	1	0
145	YAP1_YPD	1	1	1	1	1	0	0	0	0	1	1
146	YAP3_YPD	0	0	0	0	0	0	0	0	0	0	0
147	YAP5_H2O2Hi	0	0	0	0	0	0	0	0	0	0	0
148	YAP5_YPD	0	0	0	0	0	0	0	0	0	0	0
149	YAP6_H2O2Hi	0	0	0	0	0	0	0	0	0	0	0
150	YAP6_H2O2Lo	0	0	0	0	0	0	0	0	0	0	0
151	YAP6_YPD	0	0	0	0	0	0	0	0	0	0	0

No.	Sequence-set	$\mathcal{U}$	$\mathcal{N}$	$\mathcal{DN}$	$\mathcal{DN}'$	$\mathcal{D}$	A	M	MD	M_c	C	K
152	YAP7_H2O2Hi	1	1	1	1	1	0	1	1	1	1	0
153	YAP7_H2O2Lo	1	1	1	1	1	0	0	1	1	1	1
154	YHP1_YPD	0	0	0	0	0	0	0	0	0	0	0
155	YOX1_YPD	0	0	0	0	0	0	0	0	0	0	0
156	ZAP1_YPD	0	0	0	0	0	0	1	0	1	1	0
Total		46	51	70	66	60	16	35	54	49	56	50

*In the paper we show that Ste12 in BUT14 condition is most likely to be a part of a transcriptional complex in the filamentation pathway. Tec1, which is also part of this complex in this condition, is believed to make DNA contact at its consensus motif of `CATTCy`. PRIORITY-$\mathcal{DN}$ finds this motif, correctly detecting the indirect binding of Ste12 to DNA. But since this table is generated with the criterion that the learned motif should match the motif of the profiled TF, which is `ATGAAAC` for Ste12, there is a zero in this cell. Although not shown in this table, programs $\mathcal{DN}'$, $\mathcal{D}$, and C also find the Tec1 motif in this sequence-set.

**In the paper we show that Tec1 in Alpha condition is most likely to be a part of a transcriptional complex in the mating pathway. Ste12, which is also part of this complex in this condition, is believed to make DNA contact at its consensus motif of `ATGAAAC`. PRIORITY-$\mathcal{DN}$ finds this motif, correctly detecting the indirect binding of Tec1 to DNA. But since this table is generated with the criterion that the learned motif should match the motif of the profiled TF, which is `CATTCy` for Tec1, there is a zero in this cell. Although not shown in this table, programs MD, $\mathcal{D}$, M_c, C, and K also find the Ste12 motif in this sequence-set.
